# Investigation on damage evolution mechanism of various FRP strengthened concrete subjected to chemical-freeze-thaw coupling erosion

**DOI:** 10.1371/journal.pone.0303645

**Published:** 2024-05-21

**Authors:** Wei Li, Dayang Wang, Wenyuan Xu, Yongcheng Ji

**Affiliations:** School of Civil Engineering and Transportation, Northeast Forestry University, Harbin, 150040, China; Universiti Teknologi Malaysia, MALAYSIA

## Abstract

The corrosion resistance of FRP-reinforced ordinary concrete members under the combined action of harsh environments (i.e., alkaline or acidic solutions, salt solutions) and freeze-thaw cycles is still unclear. To study the mechanical and apparent deterioration of carbon/basalt/glass/aramid fiber cloth reinforced concrete under chemical and freeze-thaw coupling. Plain concrete blocks and FRP-bonded concrete blocks were fabricated. The tensile properties of the FRP sheet and epoxy resin sheet before and after chemical freezing, the compressive strength of the FRP reinforced test block, and the bending capacity of the prismatic test block pasted with FRP on the prefabricated crack side were tested. The deterioration mechanism of the test block was analyzed through the change of surface photos. Based on the experimental data, the Lam-Teng constitutive model of concrete reinforced by alkali-freeze coupling FRP is modified. The results indicate that, in terms of apparent properties, with the increase in the duration of chemical freeze-thaw erosion, the surface of epoxy resin sheets exhibits an increase in pores, along with the emergence of small cracks and wrinkles. The texture of FRP sheets becomes blurred, and cracks and wrinkles appear on the surface. In terms of failure modes, as the number of chemical coupling erosion cycles increases, the location of failure in epoxy resin sheets becomes uncertain, and the failure plane tilts towards the direction of the applied load. The failure mode of FRP sheets remains unchanged. However, the bonding strength between FRP sheets and concrete decreases, resulting in a weakened reinforcement effect. In terms of mechanical properties, FRP sheets undergo the most severe degradation in the coupled environment of acid freeze-thaw cycles. Among them, GFRP experiences the largest degradation in tensile strength, reaching up to 30.17%. In terms of tensile performance, the sheets rank from highest to lowest as follows: CFRP, BFRP, AFRP, and GFRP.As the duration of chemical freeze-coupled erosion increases, the loss rate of compressive strength for specimens bonded with CFRP is the smallest (9.62% in salt freeze-thaw environment), while the loss rate of bearing capacity is higher for specimens reinforced with GFRP (33.8% in acid freeze-thaw environment). In contrast, the loss rate of bearing capacity is lower for specimens reinforced with CFRP (13.6% in salt freeze-thaw environment), but still higher for specimens reinforced with GFRP (25.8% in acid freeze-thaw environment).

## Introduction

In 1981, the Swedish engineer Meier proposed the concept of fiber-reinforced materials (FRP) and reinforced it with adhesive CFRP for the first time on Ebach Bridge. Since then, fiber-reinforced materials have been developed with many different types of FRP materials and have been widely used in other fields [1]. It can be roughly divided into three categories: carbon fiber CFRP, polymer fiber including aramid fiber (AFRP), and inorganic fiber including glass fiber (GFRP) [2,3], which has a good repair and protection effect on concrete structures [4]. Many existing buildings in the construction industry are faced with the dilemma of maintenance and reconstruction [5]. Compared with other repair methods, FRP materials have been widely studied because of their characteristics, such as corrosion resistance, high strength, and light unit weight [6], especially their corrosion resistance in harsh environments. At the same time, compared with other materials, FRP materials require less material for the substructure with the same strength due to their lightweight, which can effectively reduce carbon emissions [7]. Although FRP materials have been studied for decades, from birth to now, and much progress has been made, many problems still need to be solved. For example, corrosion in harsh environments (i.e., water, alkaline or acidic solutions, salt solutions) results in severe damage to degradation. The relative slip of concrete and FRP fiber cloth leads to problems such as interface debonding, which leads to the failure of the whole structure [8–14]. After erosion, the strength, stiffness, and other properties of the concrete structure reinforced with FRP fiber cloth will decrease [15]. Zhou et al. studied the bonding ability of concrete bond samples strengthened by CFRP after a sulfate attack. The experiment showed that sulfate attack seriously eroded the bonding property of the interface between CFRP and concrete, and the bonding property decreased under the circulation of high temperature and high humidity of sulfate solution [16]. In winter, civil structures exposed to Marine environments or deicing salts are usually affected by the saline-alkali environment, and salt erosion causes the intermolecular chemical bonds within the matrix and fiber-matrix interface to break or generate strain [17]. At the same time, the mechanical degradation of polymer resins commonly used to bond FRP materials will produce undesirable effects, such as plasticization and hydrolysis under humidity conditions [18]. The freeze-thaw cycle and its related coupling effects will deteriorate FRP composites [19]. FRP materials will become brittle under freezing conditions, absorb water, and expand during freezing, resulting in micro-cracks, which are especially prone to occur at the interface between FRP materials and substrates [20]. In addition, the high pressure in concrete leads to the growth of ice crystals that would be generated during repeated freeze-thaw cycles and produce micro-cracks, thus reducing the bond strength between FRP and concrete [21]. Therefore, the increase in the bearing capacity of FRP-reinforced specimens after the freeze-thaw cycle is lower than that of FRP-reinforced specimens at room temperature [22]. FRP generally exhibits high specific strength, light weight, and high modulus, making it suitable for concrete reinforcement. Additionally, CFRP boasts excellent corrosion resistance. GFRP offers good design flexibility and has a linear expansion coefficient close to that of concrete. AFRP, on the other hand, exhibits superior fatigue resistance. BFRP falls between CFRP and GFRP in terms of strength, but it outperforms CFRP in ductility and significantly exceeds the other three reinforcement materials in high-temperature resistance. Therefore, selecting the appropriate type of FRP for a specific environment can maximize its characteristics and performance.

In addition, the failure mode of the specimen will also change after the freeze-thaw cycle. The cohesive fracture of concrete without environmental action changes to the cohesive interface failure after multiple cycles, and the deterioration of FRP and coagulation interface is relatively more severe under the freeze-thaw cycle [23]. The salt-freeze-thaw coupling test shows that the strength and ductility of specimens restrained by CFRP and GFRP significantly decrease, but the stiffness is unaffected. Compared with specimens subjected to room temperature and dry and wet cycling, the failure mode of specimens subjected to freeze-thaw action is more disastrous [24]. Masmoudi et al. [25] studied the freeze-thaw cycling effects of concrete reinforced with glass fiber reinforced pipes and mixed with decommissioned wind turbine fiber materials. The confinement provided by the glass fiber reinforced pipes significantly improved the compressive strength of all samples and had a positive impact on the freeze-thaw durability of enclosed samples. Zou et al. [26] studied the durability of CFRP and GFRP bonded pre-cracked concrete beams under three unfavorable conditions: freeze-thaw cycling, sulfate attack, and coupled salt-freeze erosion. After 400 hours of exposure to these three adverse conditions, the strength loss of CFRP-bonded concrete flexural specimens was 42.0%, 14.6%, and 66.3%, respectively. Similarly, the strength loss of GFRP-bonded concrete flexural specimens was 52.3%, 21.9%, and 67%, respectively. Maras et al. [27] studied the performance of reinforced concrete beam column connections reinforced with CFRP, GFRP, and AFRP under load. Compared with the control specimens, the ultimate load increased by a maximum of 12.5% after CFRP reinforcement. CFRP composite materials can be a suitable alternative to significantly improve structural performance. A study was conducted on the axial compression test of a new type of FRP made outer tube, steel made inner tube composition, and a space filled concrete mixed column. It was found that GFRP tubes enhance structural behavior by providing constraints on concrete and additional shear resistance [28].Yan et al. [29]studied the compressive performance of FRP wrapped 3D printed ultra-high performance concrete components, and the reinforcement of FRP significantly improved the strength and deformation capacity of ultra-high performance concrete components. Kantarci et al. [30] studied the bending behavior of reinforced concrete beams reinforced with aluminum honeycomb sandwich panels and CFRP with different cross-sectional configurations (i.e. supports and intermediate parts). The reinforced concrete beams reinforced with 15 mm thick aluminum honeycomb sandwich panels and CFRP showed higher bending resistance and stronger ductility than similar products.

In an acidic environment and seawater environment, it is found that the bond durability of FRP and concrete and steel structure shows a downward trend, and the alkaline environment with a pH value of 12 has the most significant influence on the bond of FRP and steel structure. Although the steel structure will not suffer more corrosion damage in the alkaline environment, the alkaline environment dramatically affects the interface between FRP and steel structure, resulting in a considerable decline in bond strength [31]. In addition, the corrosive environment not only reduces the ultimate bearing capacity of the beam but also changes the failure form of the beam, and the bond between concrete and FRP materials will degrade in the corrosive environment [32]. Moreover, the stripping of the loading end will change the tensile force distribution along the bonding interface into an uneven nonlinear distribution, which significantly increases the length of the tensile force transferred from the plate to the concrete specimen [33].

In summary, there is still a lack of research on the performance of FRP-reinforced concrete members under the coupled effects of harsh chemical environments and freeze-thaw cycles. The durability performance is still unclear and further exploration is urgently needed. Although efforts have been made to improve the effectiveness of glass fiber-reinforced materials, their applications still need to be expanded to solve simple structural problems. This may be related to the need for design specifications, the wide variation in material properties of FRP materials, and the limited knowledge of engineers about the application and structure of FRP materials [34]. From the perspective of chemical-freeze-thaw coupling erosion, this paper tries to analyze the durability influencing factors of concrete reinforced with fiber cloth in practical engineering and study the mechanical and apparent deterioration laws of concrete reinforced with carbon/basalt/glass/aramid fiber cloth under chemical and freeze-thaw coupling.

## Experimental programs

### Specimen and erosion environment

This experiment used concrete with a design strength of 30 MPa. Its composition materials were water, cement, fine aggregate, and coarse aggregate, with a ratio of 0.54:1:1.64:3.02. The cement is Swan P.O42.5 ordinary Portland cement produced by Jilin Yatai Cement Co., LTD. The fine aggregate is medium sand with a fineness modulus 2.4, taken from local river sand. The coarse aggregate is gravel. The 5-10mm particle size accounted for 30%, and 10-20mm accounted for 70%.

**Fig 1:**
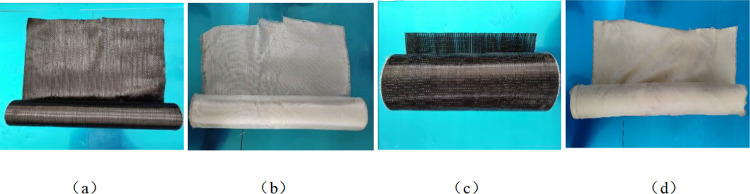
Four kinds of fiber composites:(a) CFRP, (b) GFRP, (c) BFRP, (d) AFRP.

**Fig 2:**
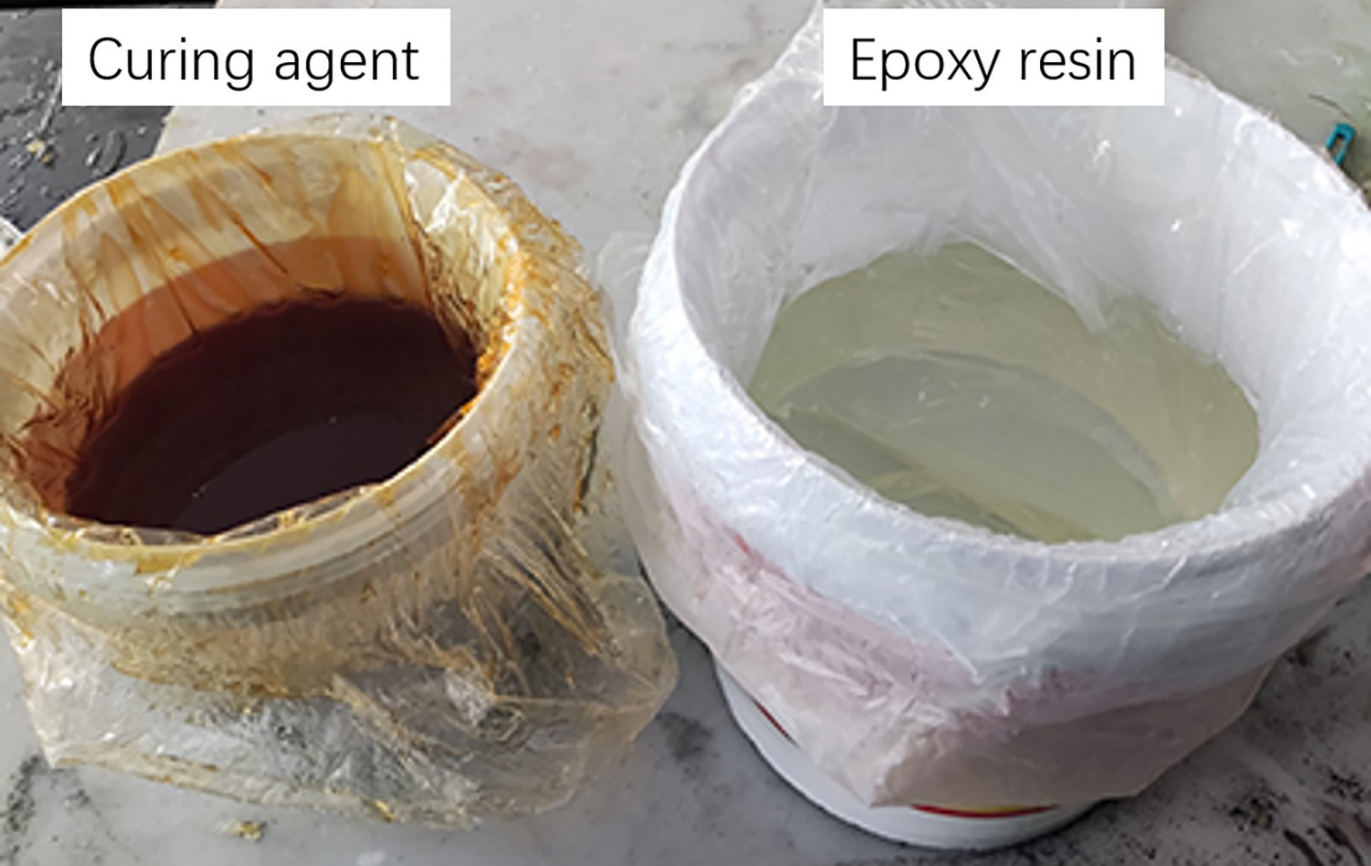
Epoxy resin glue.

Four representative fiber-reinforced composites (FRP) were selected for concrete reinforcement, namely carbon fiber composite (CFRP), basalt fiber composite (BFRP), glass fiber composite (GFRP), and aramid fiber composite (AFRP), as shown in Fig 1. Fiber composite material is bonded to concrete by epoxy resin adhesive. The adhesive is divided into A and B components, including curing agent and epoxy resin (Fig 2). After seven days, the curing degree of epoxy resin is 95%. The epoxy resin and CFRP manufacturer is Shanghai Jingdong Construction Technology Co., LTD. (Shanghai, China). The primary mechanical indexes of the four fiber composite materials and epoxy resin adhesive are shown in Table 1.

**Table 1:** Properties of fiber composites and epoxy resin adhesives.

Material	Strength/MPa	Elasticity modulus/GPa	Elongation at break/%
CFRP	3520	267	1.78
BFRP	3000	120	1.60
GFRP	2500	80	2.3
AFRP	2106	117.8	1.75
Epoxy resin	54.3	2.7	2.25

### Preparation of fiber cloth sheet and reinforced concrete

**Fig 3:**
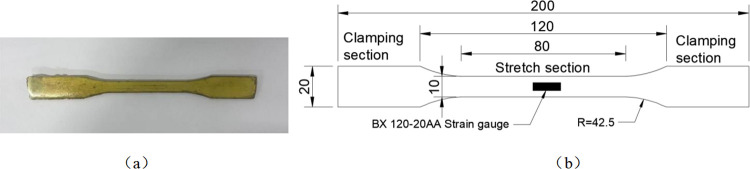
Epoxy resin: (a) Epoxy resin specimen, (b)Specimen size diagram (unit: mm).

**Fig 4:**
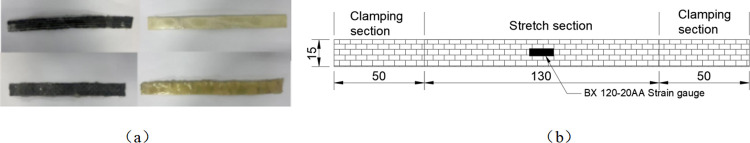
Fiber composite material specimen: (a) Fiber composite specimen, (b) FRP sheet size (unit: mm).

In order to test the effect of chemical-freeze-thaw cycle erosion from the material point of view, epoxy resin adhesive and fiber sheet samples were prepared. Epoxy resin adhesive sample, as shown in Fig 3), epoxy resin adhesive is composed of curing agent and resin and mixed with a mass ratio of 1:2. A fiber cloth (standard thickness of 0.165 mm) was coated with epoxy resin adhesive on both sides to make a fiber composite specimen as shown in Fig 4.

**Fig 5:**
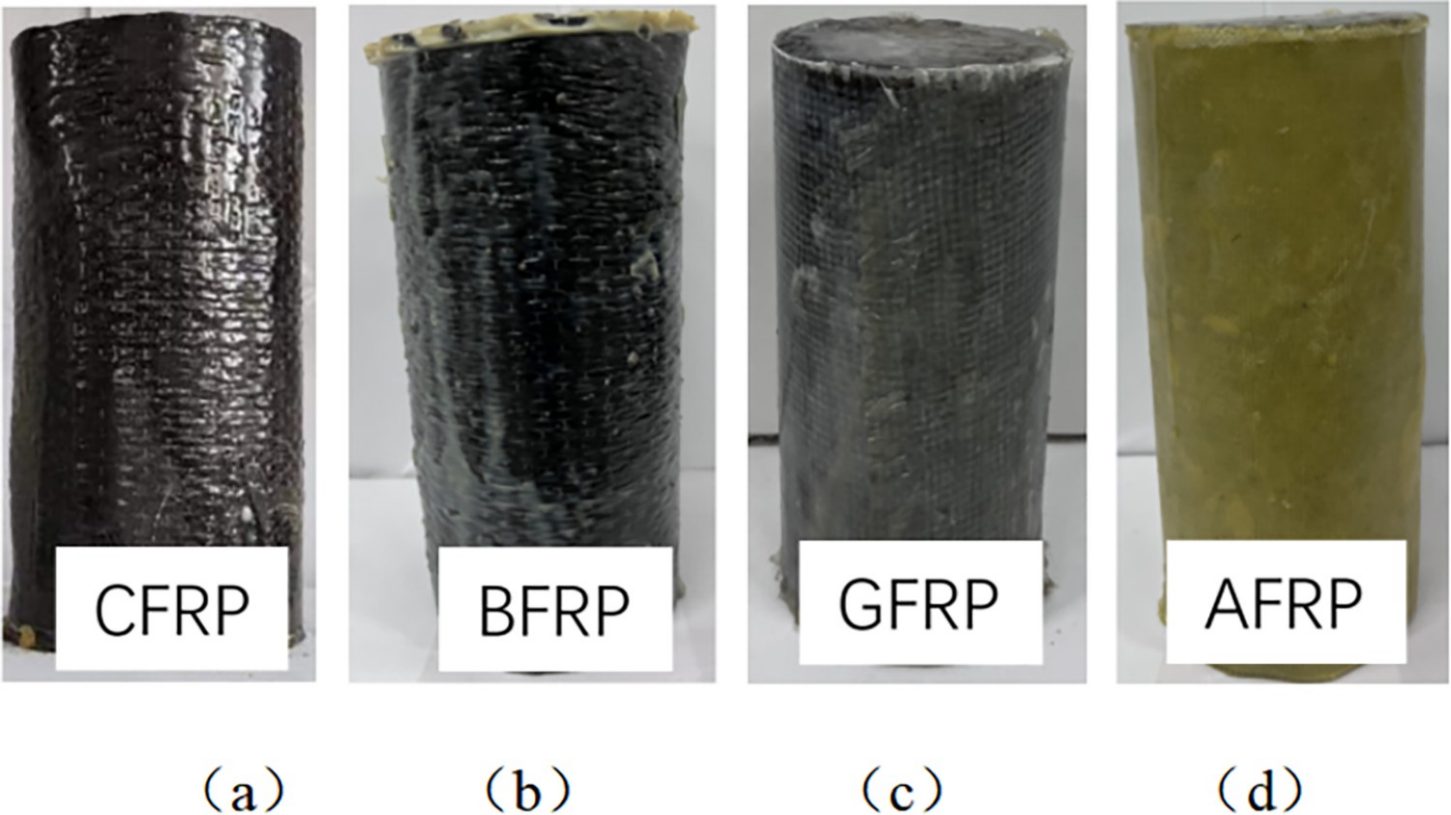
Fiber cloth reinforced concrete compression specimen: (a) CFRP, (b) BFRP, (c) GFRP, (d) AFRP.

FRP materials possess high elastic modulus and tensile strength. When wrapped around concrete, they can increase the compressive resistance of concrete columns due to the confining pressure effect. Additionally, attaching FRP materials to the underside of beams can enhance the bearing capacity and ductility of concrete beams. Furthermore, for damaged concrete structures exhibiting issues such as cracks and peeling, FRP reinforcement technology can effectively repair and strengthen them, restoring their original bearing capacity and functionality. Cylindrical specimens with a diameter of 100mm and a height of 200mm were made, as well as prismatic specimens with a length of 100mm, a width of 100mm, and a height of 400mm. After mold removal, the specimens were cured for 24 hours under natural conditions and put at room temperature (20±2)°C, relative humidity 95%, and curing room for 28 days. The concrete cylindrical specimen is pasted with fiber cloth using the wet adhesion method. First, the outer surface of the specimen is polished and smooth, and the surface of each specimen is cleaned with a brush to remove unnecessary dust and cement particles. The selected cylinder is wrapped in a single fiber cloth (200 mm wide, 340mm long, 0.165mm thick) and bonded with epoxy resin. The outer wrapping circumference of the fiber cloth is 25mm so that the CFRP material is not wasted but also to prevent the interface from sticking. The top and bottom of the FRP-wrapped cylinders are covered with epoxy resin (approximately 5mm thick) to avoid direct contact with the concrete surface with the chemical environment through unwrapped areas. According to the manufacturer’s technical data, the concrete specimen reinforced with fiber cloth must be cured for seven days at room temperature. The concrete compression specimen reinforced with fiber cloth is shown in Fig 5.

**Fig 6:**
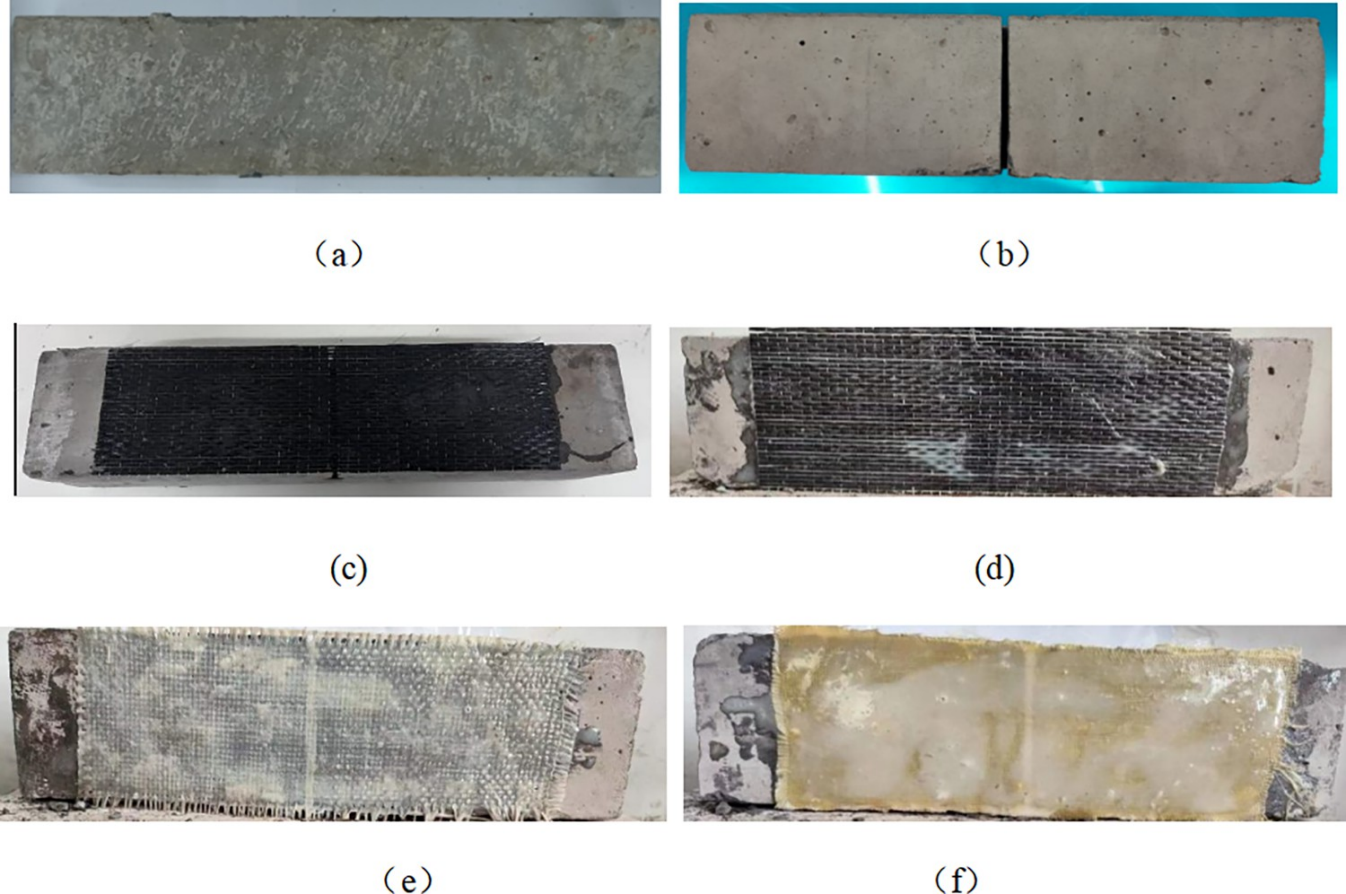
FRP reinforced bending specimen:(a) Unreinforced bending specimen, (b) Slotted bending specimen, (c) CFRP reinforced bending specimen, (d) BFRP reinforced bending specimen, (e) GFRP reinforced bending specimen, (f) AFRP reinforced bending specimen.

The bending specimens of concrete prisms are manufactured according to the bonding test method in the specification (as shown in Fig 6). In order to study the influence of FRP strengthening on the bending properties and durability of the bending specimens, the bending specimens of unreinforced concrete (6(a)~(b)) and the bending specimens of FRP reinforced concrete (6(c)~(f)) are designed. Cracks with a height of 40mm are prefabricated on the mid-span tension side of the unreinforced concrete bending specimen, as shown in Fig 6(A) and 6(B). Visually verify that there are no microcracks near the cut tip, then clean the specimen with a spray gun and rinse with water to remove concrete residue. The fibers are surface-polished before being attached to them. Epoxy adhesive is applied evenly to the jagged concrete surface. One layer of unidirectional drying fiber cloth (100 mm wide x 300 mm long) is coated with epoxy resin glue to form a fiber cloth composite system, followed by another layer of epoxy resin on the other side and cured at room temperature for 7 days.

### Specimen exposure environment

**Fig 7:**
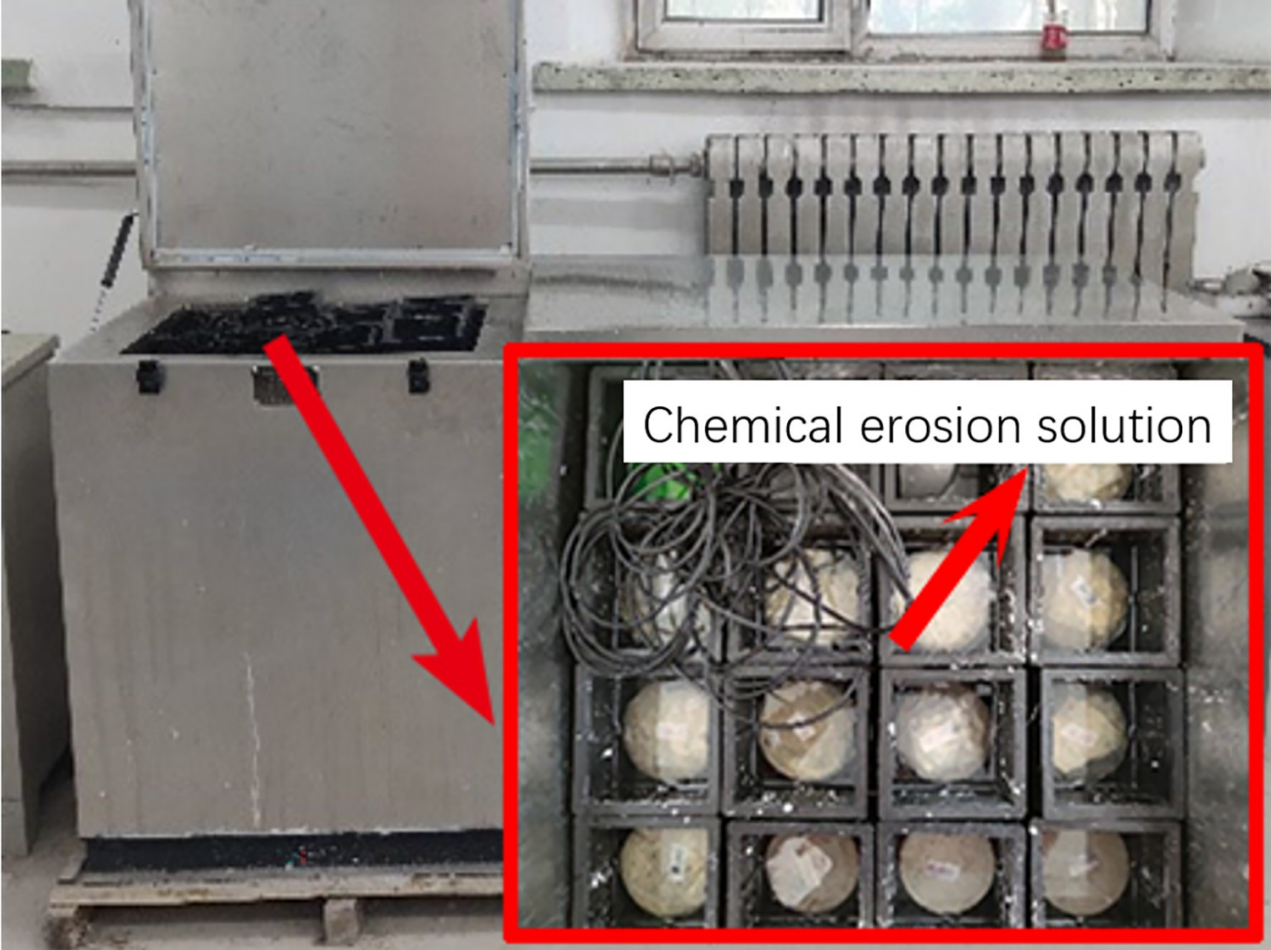
Chemistry-freeze-thaw cycle coupled immersion environment.

In order to simulate the coupled environment of chemical and freeze-thaw cyclic erosion of FRP reinforced concrete deterioration, the tests were conducted according to GB/T 50082–2009 "Standard for Experimental Methods of Long-term Performance and Durability of ordinary Concrete" and freeze-thaw cyclic experiments were conducted by using the quick-freezing method. The test equipment is shown in Fig 7. During the freeze-thaw cycle, the freeze-thaw cycle temperature was set as 5±2°C at high temperature and -17±2°C at low temperature. The freeze-thaw cycle medium was 5% H2SO4 solution with mass concentration, 2mol/L NaOH solution, or 3.5% NaCl solution with mass fraction, which were acid-freeze environment, alkali-freeze environment, salt-freeze environment coupled with thaw cycle. Epoxy resin adhesive, FRP sheet, and concrete specimen are put into a freeze-thaw circulation box. The time to complete a freeze-thaw cycle test was 4 hours, and the surface observation and mechanical test were carried out after 0, 50, and 100 freeze-thaw cycles, respectively.

### Microscopic observation

**Fig 8:**
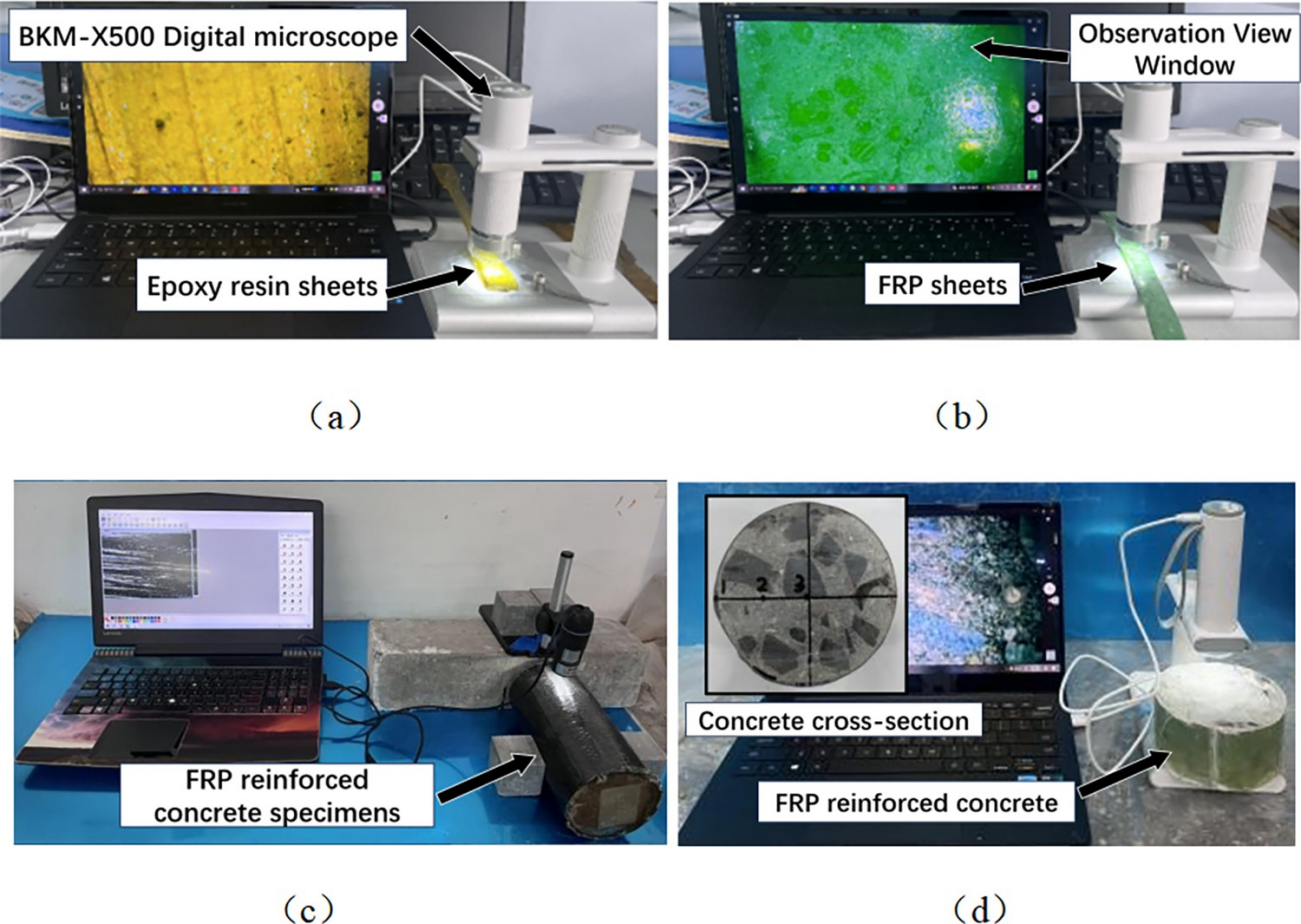
Specimen microscopic observation: (a) Epoxy resin, (b) FRP sheet, (c) FRP reinforced concrete, (d) FRP reinforced concrete cross section resin.

In civil engineering, digital microscopes can study the microstructure and properties of concrete, steel, and wood. The cracks and damage inside concrete can be observed by digital microscope, the microstructure and mechanical properties of FRP sheets can be analyzed, and the fiber structure and durability of FRP sheets can be studied. This information is essential for evaluating the properties of materials and designing civil engineering structures. In order to study the degree of damage to the concrete specimen, epoxy resin adhesive, and four kinds of FRP sheet in a coupled erosion environment, the surface of the specimen was observed using a BKM-X500 digital microscope. Samples of acid-freeze-thaw, alkali-freeze-thaw, and salt-freeze-thaw cycles were selected for 50 and 100 freeze-thaw cycles. At the same time, the non-deteriorated sheet was observed for comparison, and the observation ratio was 100 times, as shown in Fig 8.

### Mechanical test

#### Tensile test

According to the difference of environment and cycle, the epoxy resin adhesive and CFRP, BFRP, AFRP, and GFRP sheets are grouped and numbered, with five sheets in each group, a total of 225 sheets, as shown in Fig 2 below. EPN represents epoxy resin film without coupling erosion, EPAC-F50 represents epoxy resin film that has experienced 50 times acid-freeze coupling erosion, and EPAC-F100 represents epoxy resin film that has experienced 100 times acid-freeze coupling erosion. For the fiber sheet number: CSAC-F0 represents the carbon fiber sheet without coupling erosion; CSAC-F50 represents the carbon fiber sheet that has experienced 50 times acid-freeze coupling erosion; CFA-F100 represents the carbon fiber sheet that has experienced 100 times acid-freeze coupling erosion. Other fiber sheets are numbered in Table 2 below.

**Table 2:** Numbers of epoxy resin and fiber sheets.

Sheet type	Freeze-thaw cycle	Acid-freeze coupling erosion specimen No.	Alkali-freeze coupling erosion specimen No.	Salt-freeze coupling erosion specimen No.
Epoxy resin	0	EPN-F0	EPN-F0	EPN-F0
CFRP sheet	0	CSN-F0	CSN-F0	CSN-F0
BFRP sheet	0	BSN-F0	BSN-F0	BSN-F0
GFRP sheet	0	GSN-F0	GSN-F0	GSN-F0
AFRP sheet	0	ASN-F0	ASN-F0	ASN-F0
Epoxy resin	50	EPAC-F50	EPAL-F50	EPSA-F50
CFRP sheet	50	CSAC-F50	CSAL-F50	CSSA-F50
BFRP sheet	50	BSAC-F50	BSAL-F50	BSSA-F50
GFRP sheet	50	GSAC-F50	GSAL-F50	GSSA-F50
AFRP sheet	50	ASAC-F50	ASAL-F50	ASSA-F50
Epoxy resin	100	EPAC-F100	EPAL-F100	EPSA-F100
CFRP sheet	100	CSAC-F100	CSAL-F100	CSSA-F100
BFRP sheet	100	BSAC-F100	BSAL-F100	BSSA-F100
GFRP sheet	100	GSAC-F100	GSAL-F100	GSSA-F100
AFRP sheet	100	ASAC-F100	ASAL-F100	ASSA-F100

**Fig 9:**
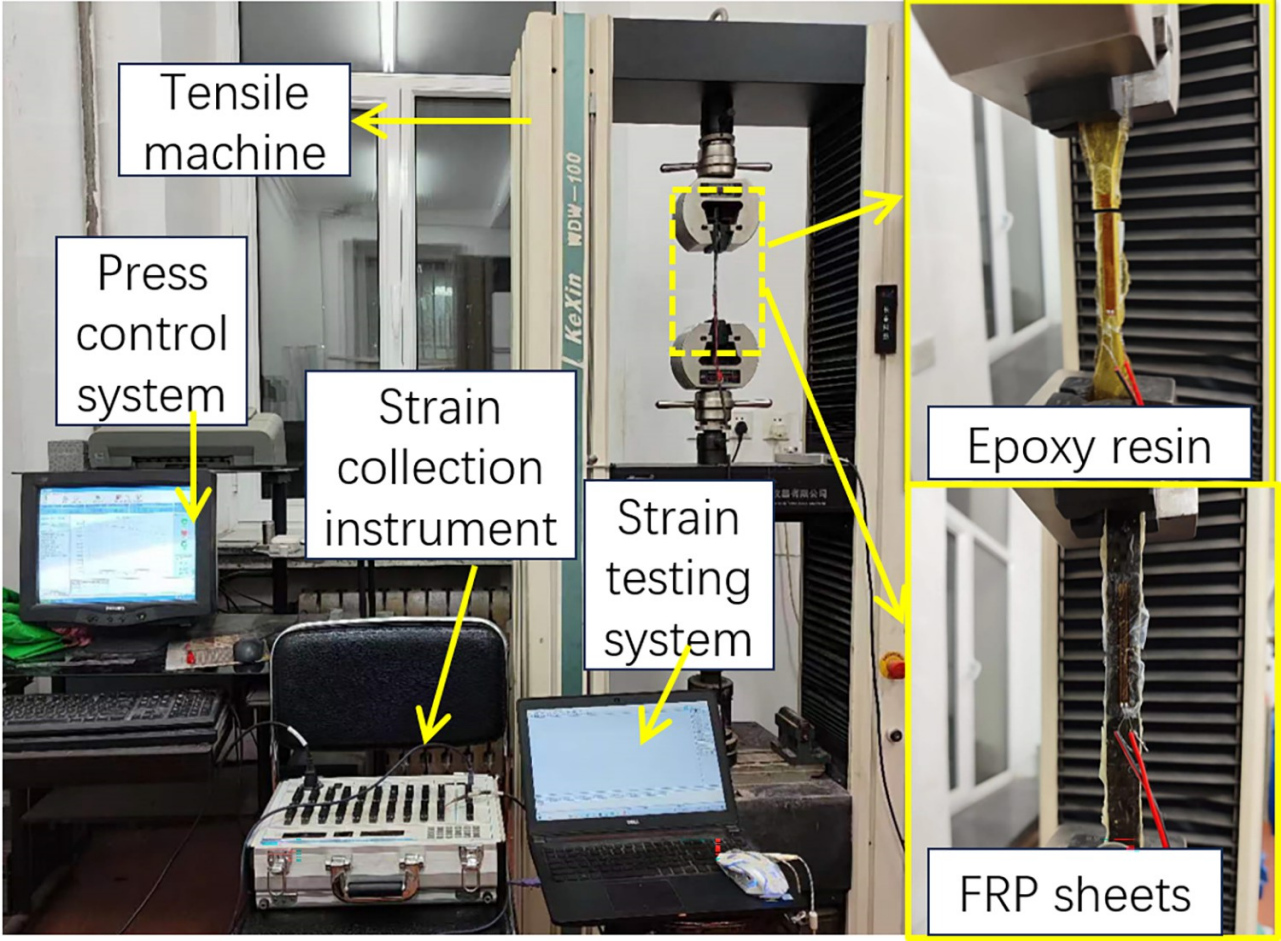
Tensile mechanical test.

In order to investigate the influence of an acid-freeze-thaw coupled erosion environment on the mechanical properties of epoxy resin adhesive and FRP sheet, the tensile tests of epoxy resin adhesive and FRP sheet were conducted. The tensile test is a loading process that comprises a mechanical control system and a data acquisition system. The former is responsible for controlling the loading rate and collecting load data, and the sheet is stretched by controlling the mechanical testing machine; the latter is responsible for collecting strain gauge data. In order to obtain the strain in the tensile direction of the sheet, the strain gauge is pasted in the middle of the standard distance segment of the sheet, and the loading rate is 2mm/min. The loading and collection process is shown in Fig 9.

#### Compression test

According to the difference in environment and period, the unreinforced specimens and FRP-reinforced specimens were grouped and numbered, with six specimens (three cylindrical test blocks and 3 prismatic test blocks) in each group, as shown in Table 3.

**Table 3:** Summary of the number of acid-freeze-thaw coupled erosion specimens.

Specimen type	Freeze-thaw cycle	Acid-freeze coupling erosion specimen No.	Alkali-freeze coupling erosion specimen No.	Salt-freeze coupling erosion specimen No.
Unreinforced specimen	0	Control group	Control group	Control group
CFRP reinforced concrete	0	CCAC-F0	CCAL-F0	CCSA-F0
BFRP reinforced concrete	0	BCAC-F0	BCAL-F0	BCSA-F0
GFRP reinforced concrete	0	GCAC-F0	GCAL-F0	GCSA-F0
AFRP reinforced concrete	0	ACAC-F0	ACAL-F0	ACSA-F0
Unreinforced specimen	50	Control group	Control group	Control group
CFRP reinforced concrete	50	CCAC-F50	CCAL-F50	CCSA-F50
BFRP reinforced concrete	50	BCAC-F50	BCAL-F50	BCSA-F50
GFRP reinforced concrete	50	GCAC-F50	GCAL-F50	GCSA-F50
AFRP reinforced concrete	50	ACAC-F50	ACAL-F50	ACSA-F50
Unreinforced specimen	100	Control group	Control group	Control group
CFRP reinforced concrete	100	CCAC-F100	CCAL-F100	CCSA-F100
BFRP reinforced concrete	100	BCAC-F100	BCAL-F100	BCSA-F100
GFRP reinforced concrete	100	GCAC-F100	GCAL-F100	GCSA-F100
AFRP reinforced concrete	100	ACAC-F100	ACAL-F100	ACSA-F100

**Fig 10:**
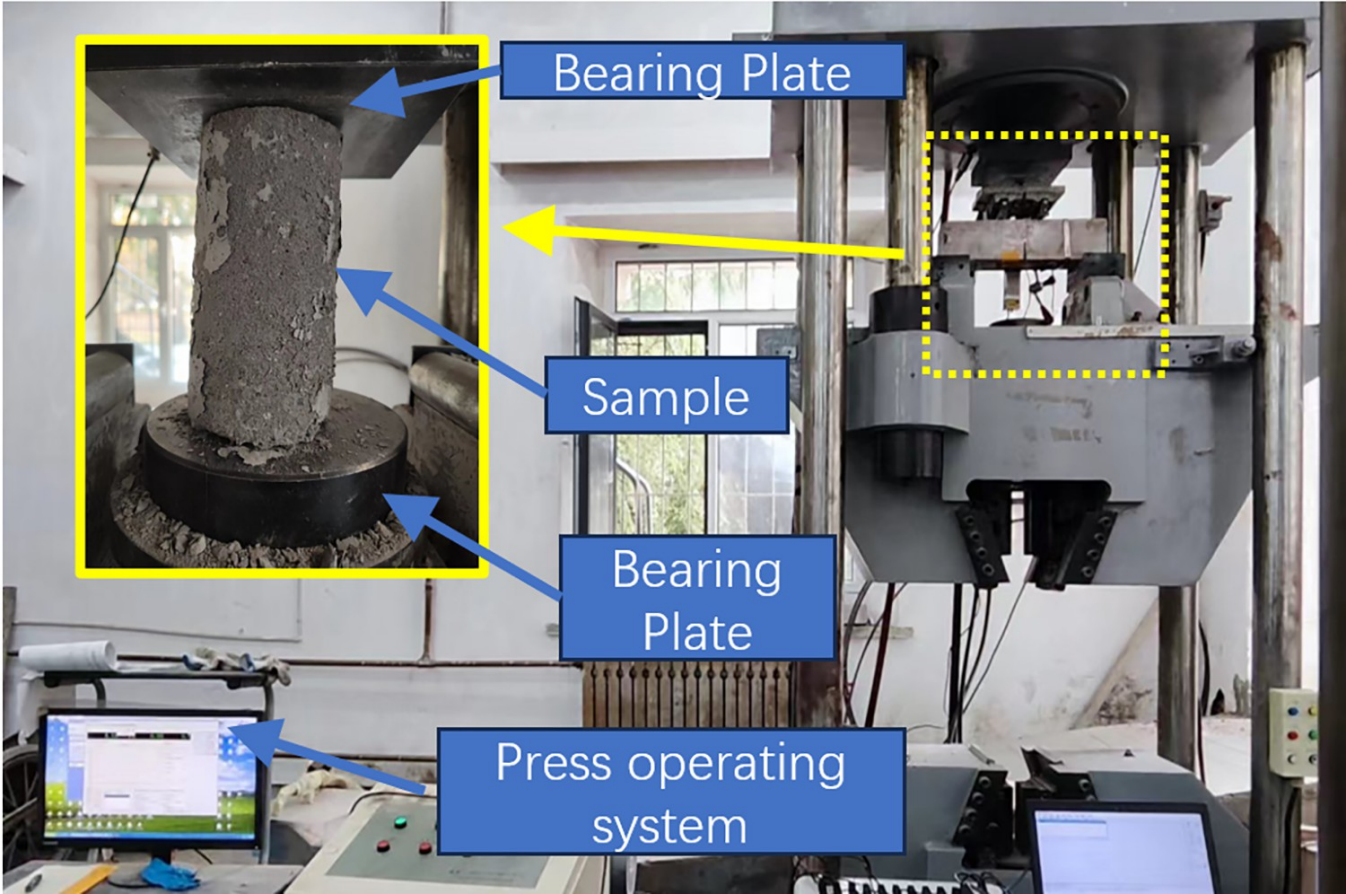
Mechanical test device.

The compressive strength of cylindrical specimens was determined according to the compressive strength test method of the standard for Testing methods of mechanical properties of ordinary Concrete (GB/T 50081–2016). The cylindrical specimen is placed between the upper and lower pressure plates, and the center of the specimen is aligned with the center of the pressure plate. In the compression test, the loading speed is 0.3MPa/s. The compression test and bending test are shown in Fig 10.

#### FRP Reinforced concrete prism

**Fig 11:**
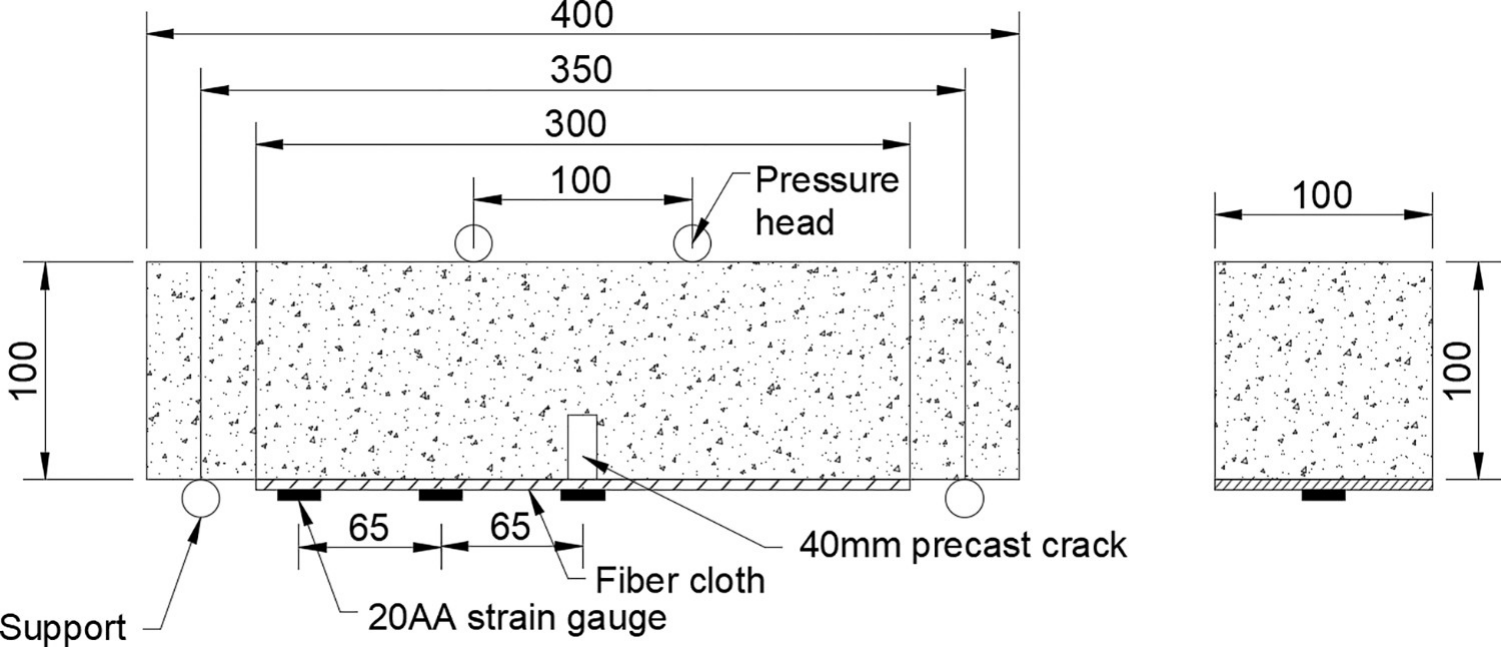
Bending specimen size and strain gauge layout (unit: mm).

The flexural load of prisms and fiber-reinforced prisms was tested according to the flexural test method of Standard for Test Methods of Mechanical Properties of Ordinary Concrete (GB/T 50081–2016). The flexure specimens’ mid-span deflection and prisms’ flexure strength were tested by displacement meter. The four-point loading test method was adopted. A steel stirrup was fixed on one side of the specimen, and the other side was pasted with a strain gauge of 2cm length at the midline of the bottom surface of the specimen (FRP surface) to measure the strain change during loading. The loading speed is set to 0.03MPa/s. In order to discuss the bonding performance between FRP and concrete, strain gauges are pasted on the surface of FRP. The dimensions, loading points, and strain gauge distribution points of FRP-reinforced specimens are shown in Fig 11.

## Microscopic analysis

### Microscopic observation of epoxy resin

**Fig 12:**
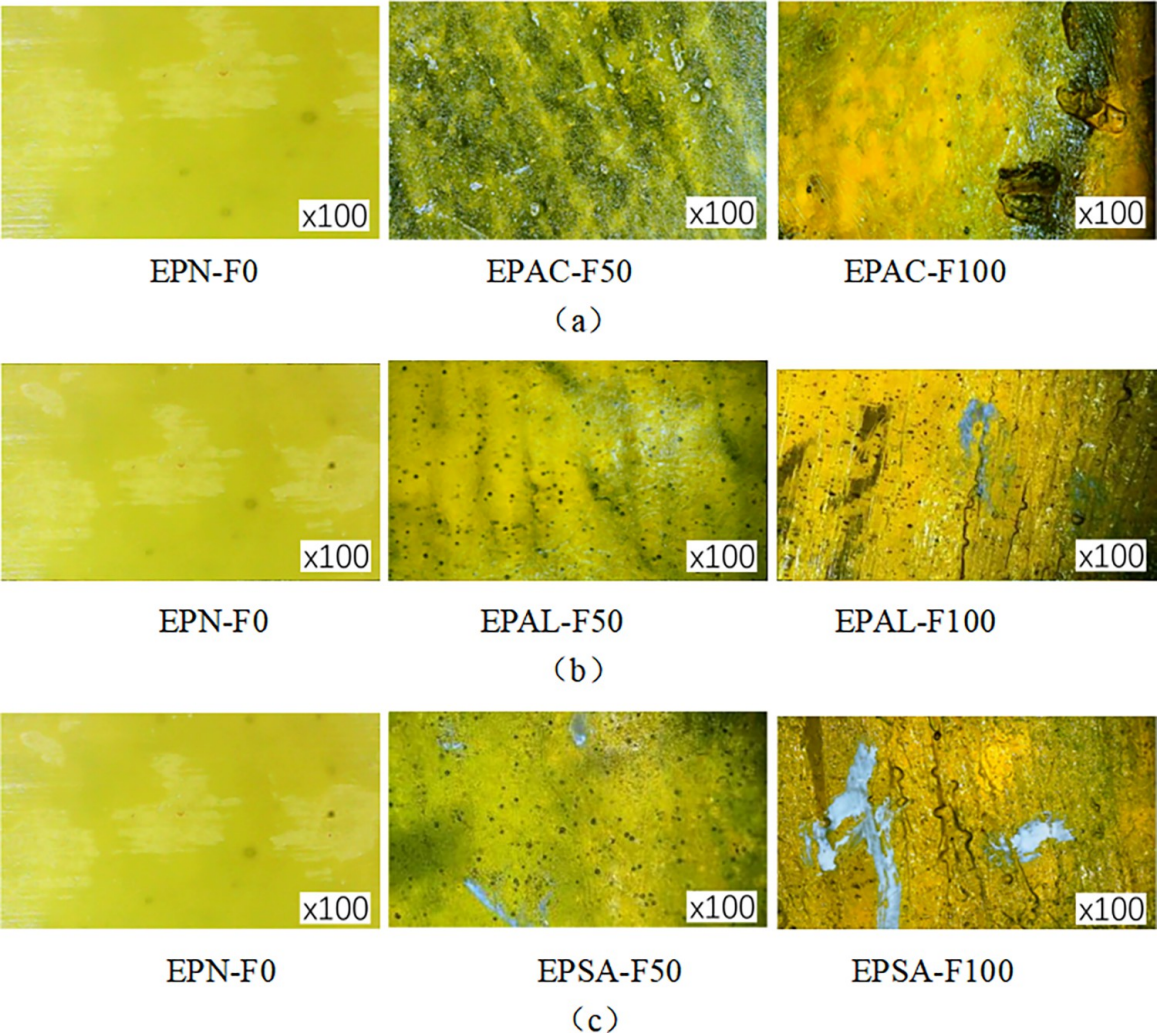
Microscopic imaging of epoxy resin: (a) acid-freeze cycle erosion, (b) alkali-freeze cycle erosion, (c) salt-freeze cycle erosion.

Fig 12(A) shows an electron microscope image of an epoxy resin specimen subjected to sulfuric acid-freeze-thaw cycle erosion. At 100 magnification factor, the epoxy resin specimen (EPAC-F50) showed small holes and irregular white spots on its surface after 50 cycles of coupling erosion. The color changes dramatically, from yellow to yellow-green. This bleaching effect is characteristic of materials typically eroded by sulfuric acid. After 100 cycles of acid-freeze-thaw coupled erosion, the surface holes of the epoxy sheet (EPAC-F100) were increasingly damaged, and ample area folds appeared. The discontinuous white spots on the surface spread randomly due to the chemical reaction of calcium ions in epoxy resin with sulfate ions in sulfuric acid solution to produce white calcium sulfate. These observations show that the epoxy initially reacts with sulfuric acid on its surface and that, over time, the sulfuric acid penetrates the inside of the epoxy, causing the overall performance of the epoxy to decline.

Fig 12(B) shows an electron microscope image of an epoxy resin specimen subjected to alkali-freeze-thaw cycle erosion. Under the magnification factor of 100 times, the surface color of the epoxy resin specimen changed obviously after 50 freeze-thaw cycles. Some white spots were observed attached to its surface, and tiny holes appeared on the surface of the epoxy resin. As the freeze-thaw cycle increases to 100 times, due to the continuous development and formation of these substances, the white adhesion expands into random shapes; tiny cracks appear, holes expand, and the surface of the epoxy resin wrinkles. Fig 12(C) shows an electron microscope image of an epoxy resin specimen subjected to salt-freeze-thaw cycle erosion. Under the amplification factor of 100 times, the surface color of the epoxy resin specimen EPS-F50 changed obviously after 50 freeze-thaw cycles. Some white sodium chloride crystals were observed attached to its surface, and tiny holes appeared on the surface of the epoxy resin. When the freeze-thaw cycle was increased to 100 times, the substances attached to the specimen EPS-F100 continued to develop and form, and the sodium chloride crystal attachments expanded and formed into random shapes, with tiny cracks and enlarged holes, and the surface of the epoxy resin showed folds. In summary, the deterioration of epoxy resin sheets increases with the increase of the chemical-freeze erosion cycle. The surface holes increase and the surface cracks and folds appear.

### Microscopic observation of FRP sheet

**Fig 13:**
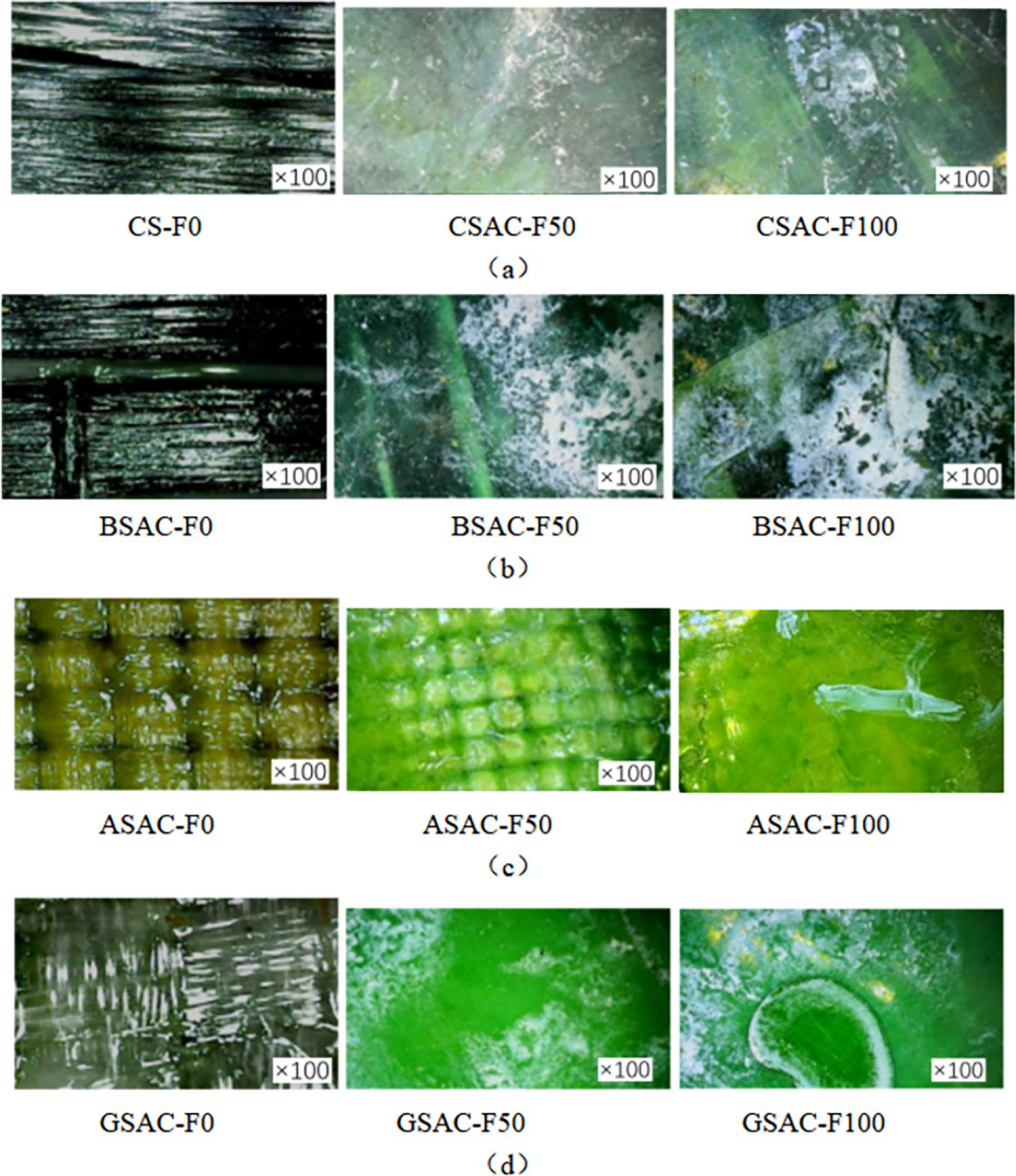
Microscopic imaging of acid-frost erosion FRP sheets: (a) CFRP, (b) BFRP, (c) GFRP, (d) AFRP.

Fig 13(A)–13(D) show the electron microscope imaging of the FRP sheet at 100 times magnification. The fiber sheets (CSAC-F0, BSAC-F0, GSAC-F0, ASAC-F0), which have not been subjected to coupled acid-freeze-thaw cycles, are bright and shiny in color, and the texture of their internal fiber cloth is visible. After 50 times of acid-freeze-thaw coupling erosion (CSAC-F50-100, BSAC-F50-100, GSAC-F50-100, ASAC-F50-100), the color changes of four kinds of fiber sheets were noticeable. Due to the corrosive effect of external sulfuric acid on the epoxy resin, the internal texture of the fiber sheet has become blurred, and there are holes. White calcium sulfate is attached to the surface of basalt fiber and glass fiber sheet. The fiber sheets (CSAC-F100-100, BSAC-F100-100, GSAC-F100-100, ASAC-F100-100) experienced 100 times coupled acid-freeze-thaw cycle erosion, similar to the surface of the epoxy resin specimen; the white adhesion expanded, the surface cracks and folds appeared.

**Fig 14:**
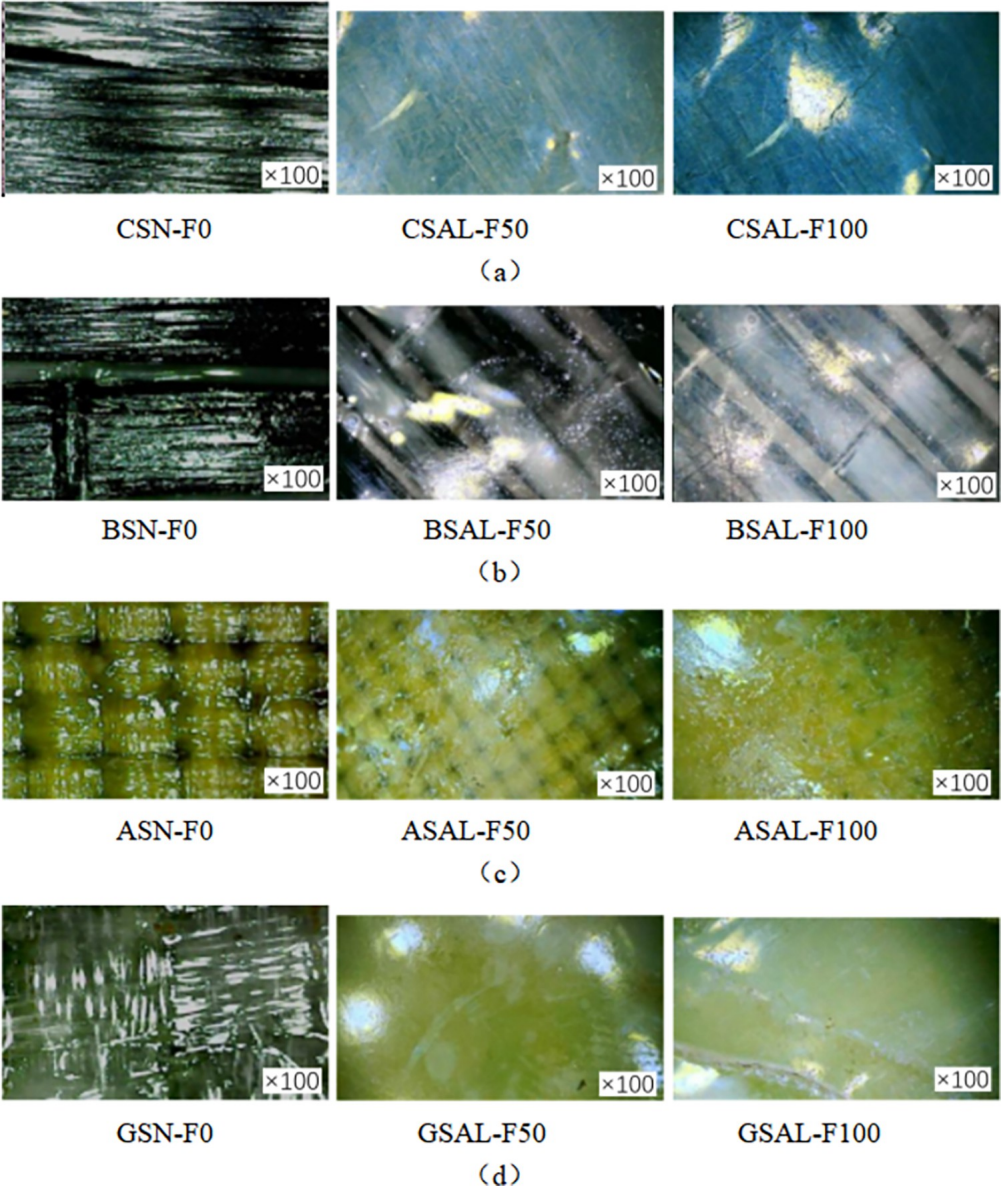
Microscopic imaging of alkali-freeze erosion FRP sheets: (a) CFRP, (b) BFRP, (c) GFRP, (d) AFRP.

Fig 14(A)–14(D) show the electron microscope imaging of the FRP sheet at 100 times magnification. The fiber sheets (CSN-F0-100, BSN-F0-100, GSN-F0-100, ASN-F0-100) that have not been subjected to alkalinity-freeze-thaw cyclic coupling erosion have bright and shiny colors, and the texture of their internal fiber cloth is visible. After 50 times of acid-freeze-thaw coupling erosion (CSAL-F50, BSAL-F50, GSAL-F50, ASAL-F50), the color changes of four kinds of fiber sheets were noticeable. Due to the corrosive effect of the external sodium hydroxide solution on the epoxy resin, the internal texture of the fiber sheet has become blurred, and there are holes. There is white adhesion on the surface of the basalt fiber and glass fiber sheet. The fiber sheets (CSAL-F100, BSAL-F100, GSAL-F100, ASAL-F100), which had undergone 100 times coupled acid-freeze-thaw cycles, were similar to the surface of the epoxy resin specimens, and the white adhesion expanded, and cracks and folds appeared on the surface.

**Fig 15:**
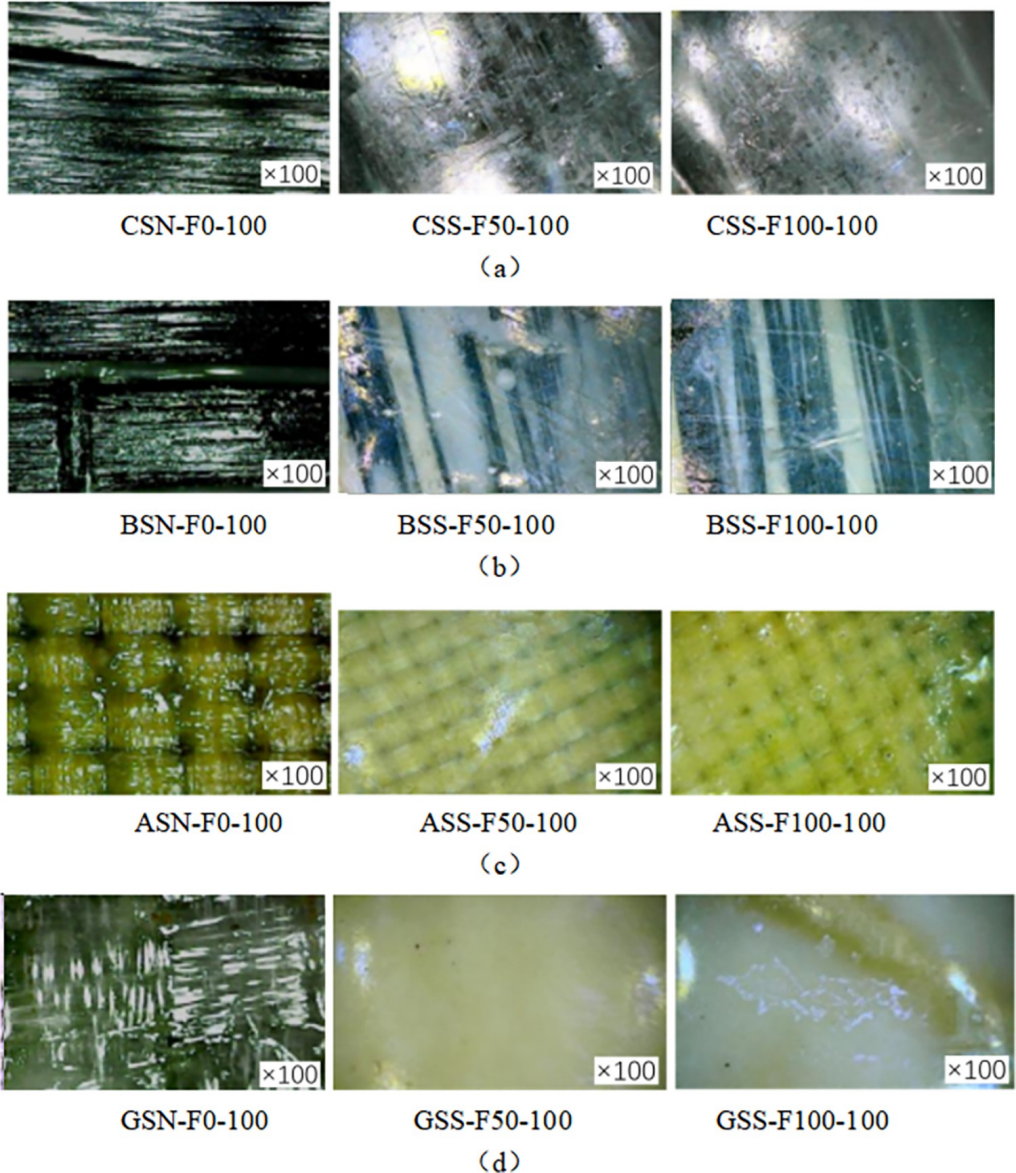
Microscopic imaging of salt-freeze erosion FRP sheets: (a) CFRP, (b) BFRP, (c) GFRP, (d) AFRP.

Fig 15(A)–15(D) show the electron microscope imaging of the FRP sheet at 100 times magnification. The fiber sheets (CSN-F0, BSN-F0, GSN-F0, ASN-F0) that have not been subjected to the coupled salt-freeze-thaw cycle erosion are bright and shiny, and the texture of the fiber fabric inside is visible. After 50 times of acid-freeze-thaw coupling erosion (CSS-F50, BSS-F50, GSS-F50, ASS-F50), the color changes of four kinds of fiber sheets were noticeable. Due to the corrosive effect of the external sodium hydroxide solution on the epoxy resin, the internal texture of the fiber sheet has become blurred, and there are holes. There is white adhesion on the surface of the basalt fiber and glass fiber sheet. After 100 cycles of coupled salt-freeze-thaw erosion (CSS-F100, BSS-F100, GSS-F100, ASS-F100), similar to the surface of the epoxy resin specimen, the white adhesion expanded, and the surface showed cracks and folds.

In summary, the surface of the FRP sheet is smooth, and the texture is clear without chemical-frost coupling erosion. After freezing and thawing in different chemical environments, the texture of FRP sheets is blurred, holes appear, and cracks and folds appear on the surface.

### Digital microscopic observation of specimen cross section

**Fig 16:**
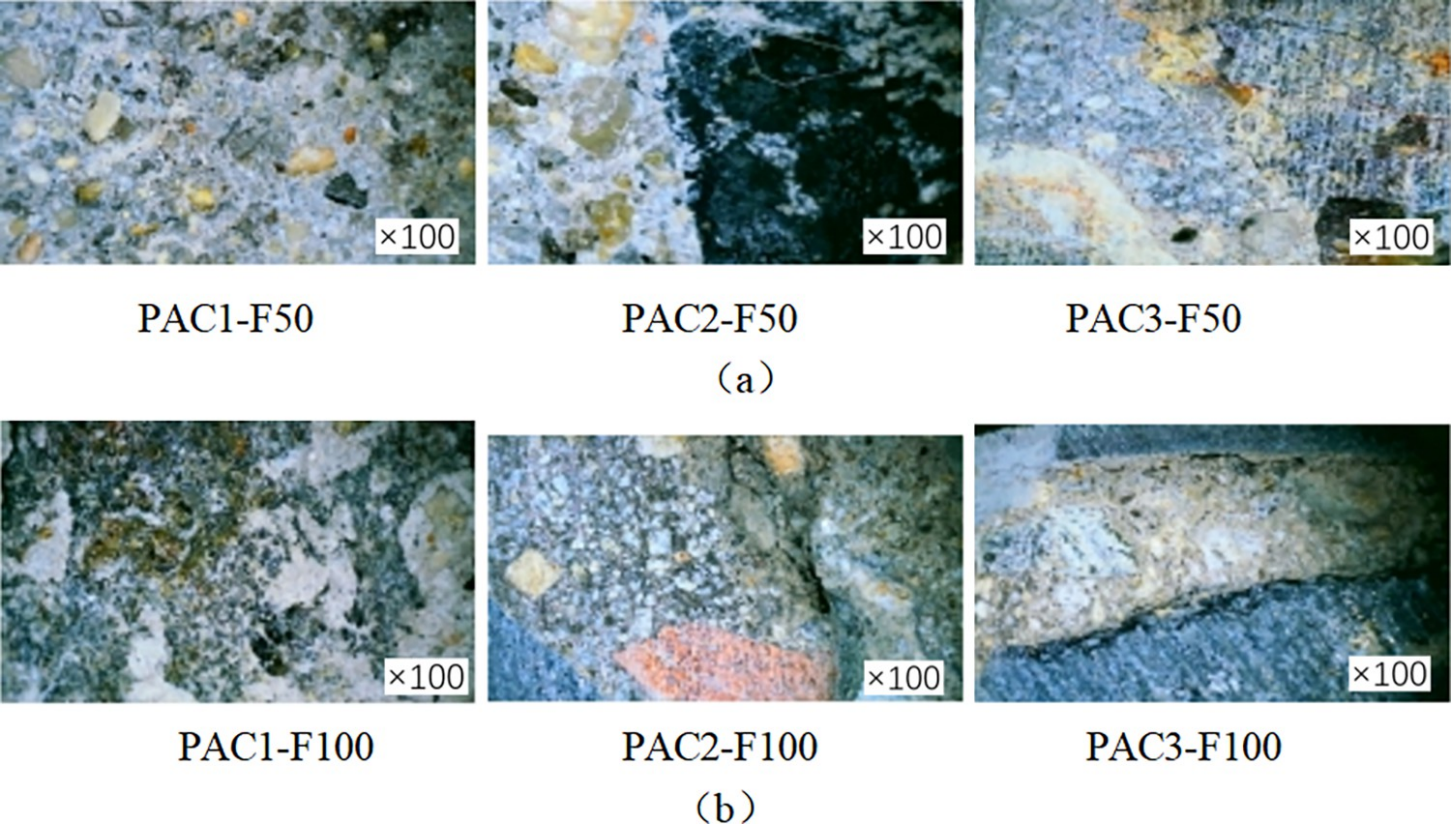
Microstructure of cross section of unreinforced specimen(a) Under 50 cycles of acid freeze-thaw erosion conditions, (b) Under 100 cycles of acid freeze-thaw erosion conditions.

**Fig 17:**
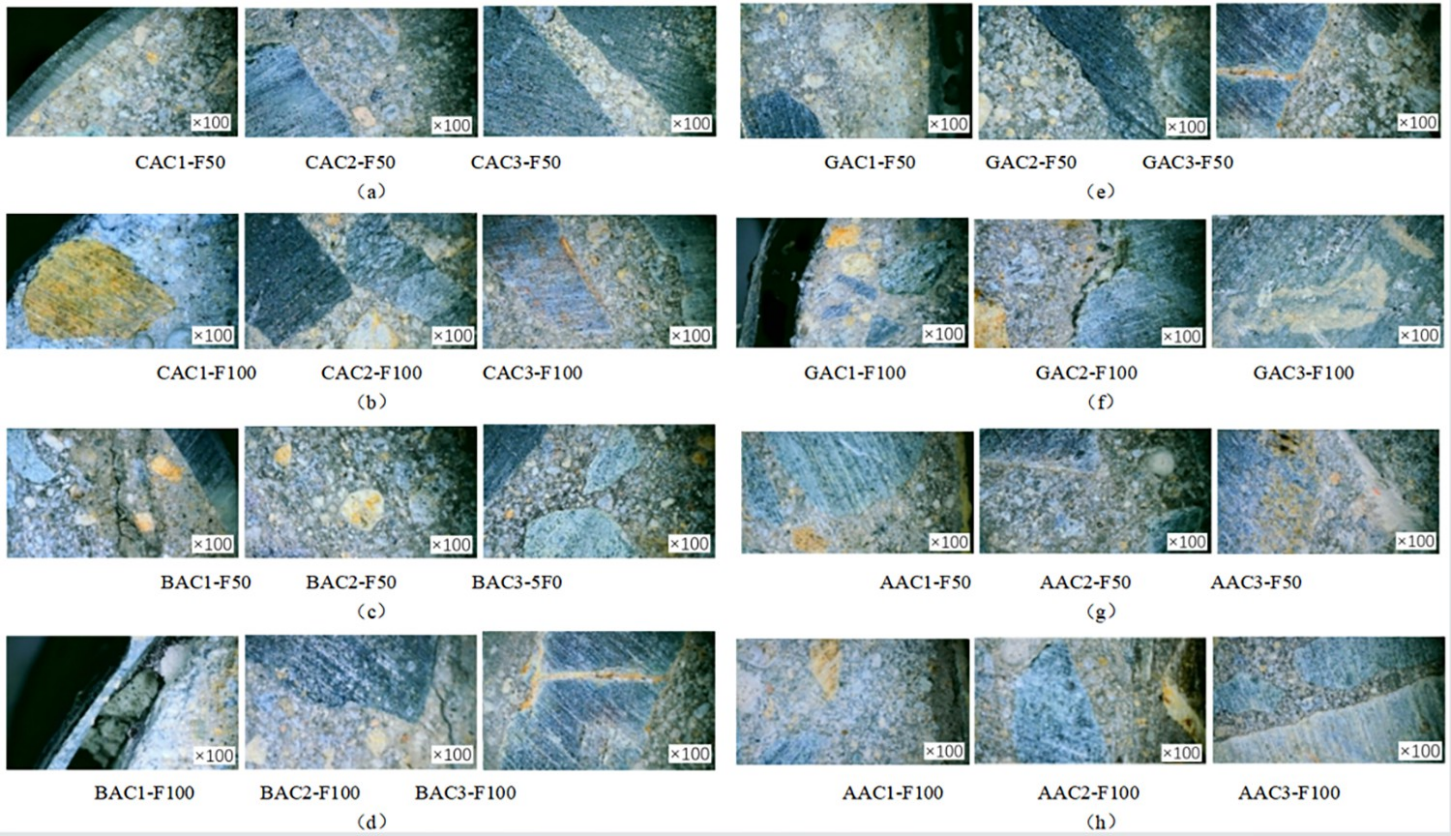
Microstructure of FRP reinforced specimens: (a) CFRP reinforced specimens subjected to 50 cycles of acid freeze cycles, (b) CFRP specimens subjected to 100 cycles of acid freeze cycles; (c) BFRP specimens subjected to 50 cycles of acid freeze cycles, (d) BFRP specimens subjected to 100 cycles of acid freeze cycles, (e) GFRP specimens subjected to 50 cycles of acid freeze cycles, (f) GFRP specimens subjected to 100 cycles of acid freeze cycles, (g) AFRP specimens subjected to 50 cycles of acid freeze cycles, (h) AFRP specimens subjected to 100 cycles of acid freeze cycles.

In order to investigate the damage to the concrete specimen caused by the coupled chemical freeze-thaw cycle erosion environment, the specimen was cut at 1/2 height. The surface of the specimen was washed and dried, and the specimen’s cross-section was observed by a digital microscope with an observation factor of 100. Fig 8(D) shows the specimen’s sampling point. Fiber cloth-reinforced specimens with 50 and 100 coupled acid-freeze-thaw cycles and axial compression specimens not reinforced with fiber cloth were selected for observation and comparative analysis, and the micromorphologies of the specimens were shown in Figs 16 and 17.

Under the coupling erosion of 50 and 100 times acid-freeze-thaw cycles, the cross-section microstructure of the unreinforced specimen is shown in Fig 16. PAC1-F100 and PAC1-F50 are the positions near the outer edge point 1 on the cross-section, and the coupling erosion is the most serious. A large amount of white calcium sulfate is generated inside the specimen. PAC2-F50 and PAC2-F100 are the locations of observation points 2, where cracks are observed in the cross-section, and a small amount of calcium sulfate is observed at PAC2-F100. The locations of observation point 3 are shown in Fig 8(D). PAC3-F50 and PAC3-F100. The internal microstructure of specimens can be seen through observation. After 100 freeze-thaw cycles, cracks appear in ITZ, the interface transition zone between aggregate and cement slurry in the cross-section of specimens PAC3-F100, the weakest concrete area.

Fig 17(A)–17(H) show the microstructure of FRP-reinforced specimens under acid-freeze-thaw cyclic erosion. Fig 17(A), 17(B), 17(F) and 17(H), respectively, show the microscopic morphology of the cross-section of the reinforced specimens with carbon fiber and aramid fiber. No apparent damage was observed under the microscope, and no micro-cracks appeared in the cross-section of the reinforced specimens. Fig 17(C) and 17(E) show basalt and glass fiber’s microscopic morphology under 50 cycles of acid-freeze-thaw erosion, respectively. It can be seen from the microscopic imaging at 1 point of cross section that there are holes and damage in the epoxy resin adhesive matrix in the FRP strip area. With the increase of the coupling erosion period, the damage became more serious, but no apparent damage, such as fracture and wire drawing, was found. The freeze-thaw cycle hurts the bond interface between composite materials and concrete. After all, when pasting FRP or resin curing process, the bond interface between FRP and concrete will inevitably have pores, cracks, and impurities. In the melting state, water is immersed in these gaps, and when the temperature is lower than 0°C, the water icing volume expansion generates pressure. Cracks occur when the pressure exceeds the strength of the material. Cracks appeared on the surfaces of BAC2-F100 and GAC2-F100, and the crack propagation was evident with the increase of the coupling erosion period, as shown in Fig 17(D) and 17(F). Compared with the unreinforced specimens, due to the encapsulation of epoxy resin and fiber cloth, no apparent cracks, holes, or other deterioration damage were found at cross-section point 3.

From the appearance and development of microcracks, the damage degree of the specimens is the most serious from the largest to the smallest, followed by the glass fiber and basalt fiber reinforced specimens and the carbon fiber and aramid fiber reinforced specimens. These cracks expand and extend with the increase of the freeze-thaw cycle. The above microscopic analysis shows that FRP reinforcement can effectively improve the durability of concrete in the coupled environment of the acid-freeze-thaw cycle, and carbon fiber and aramid fiber have the best protection effect on concrete, followed by basalt fiber and glass fiber. In the coupled environment of the acid-freeze-thaw cycle combined with the macroscopic and microscopic observation results, the specimen’s concrete surface matrix falls off, exposing coarse aggregate, and the micro-cracks appear earlier and develop more rapidly. The damage to the specimen comes from two aspects: the extrusion pressure of calcium sulfate, the product generated by the reaction of sulfuric acid and concrete, and the frost swelling force generated by the freeze-thaw cycle.

**Fig 18:**
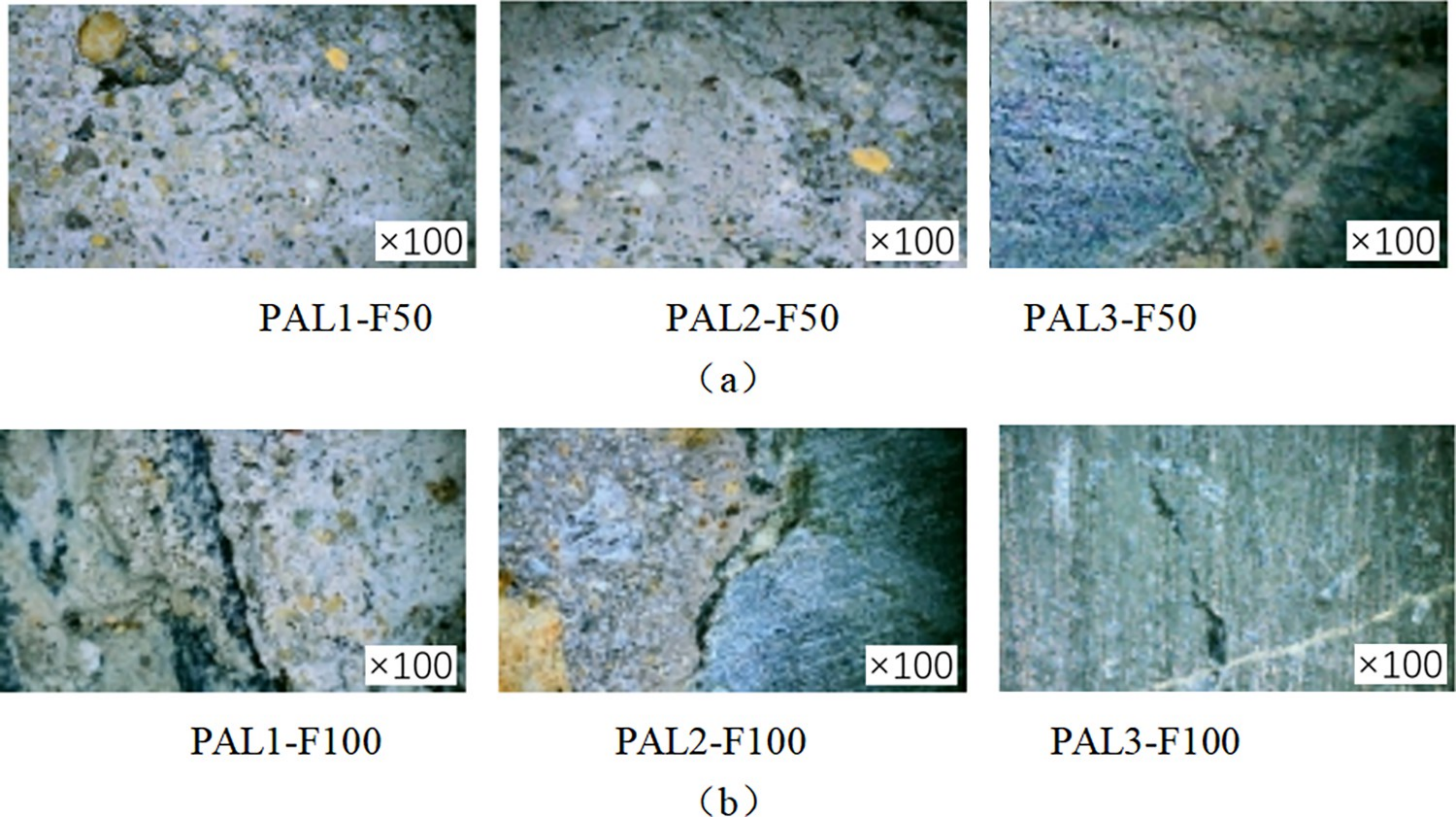
Microstructure of cross section of unreinforced specimen:(a) Under 50 cycles of alkaline freeze-thaw erosion conditions, (b) Under 100 cycles of alkaline freeze-thaw erosion conditions.

**Fig 19:**
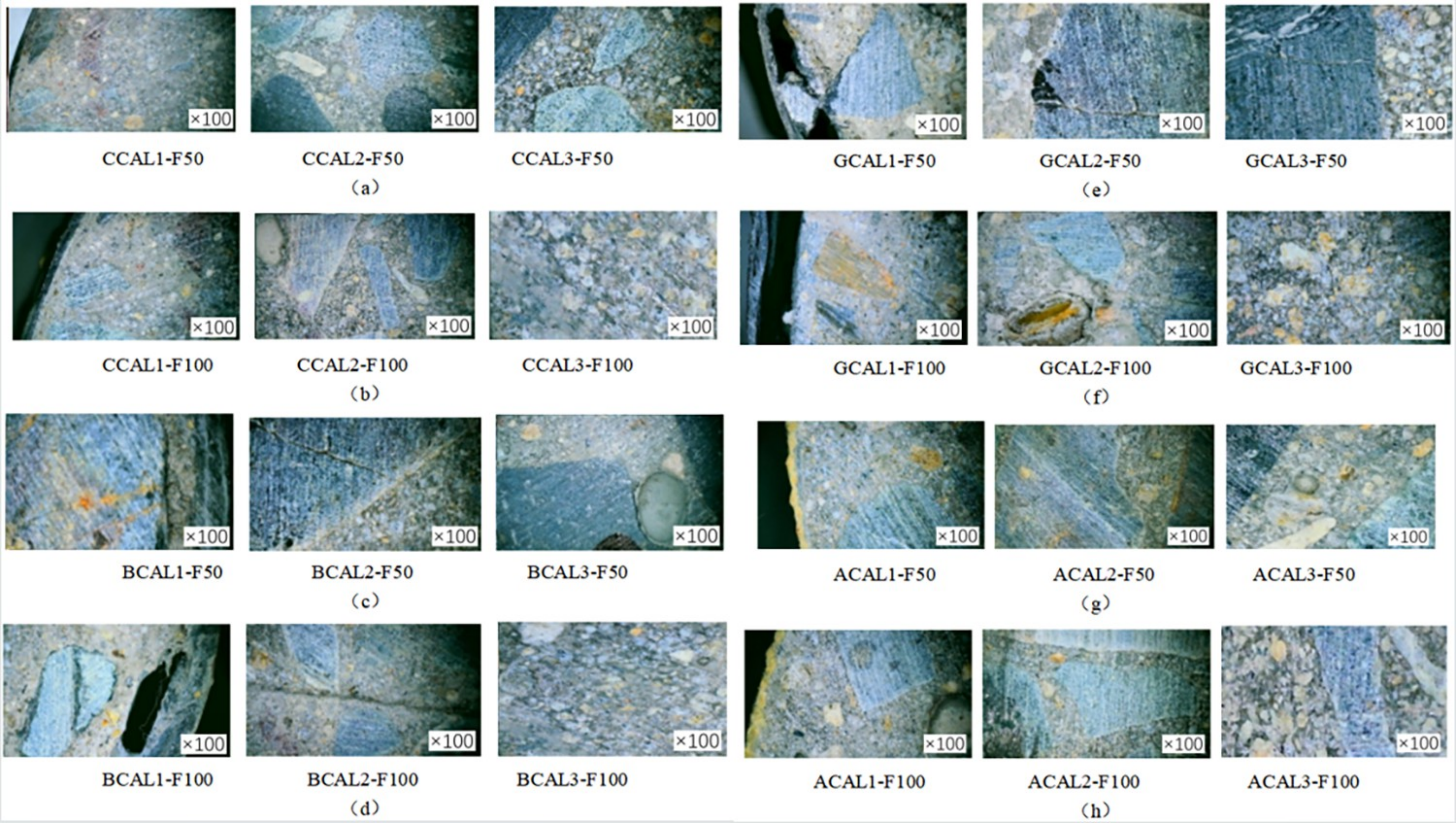
Microstructure of the FRP reinforced specimen: (a) CFRP reinforced specimens subjected to 50 cycles of alkali freeze cycles, (b) CFRP specimens subjected to 100 cycles of alkali freeze cycles; (c) BFRP specimens subjected to 50 cycles of alkali freeze cycles, (d) BFRP specimens subjected to 100 cycles of alkali freeze cycles, (e) GFRP specimens subjected to 50 cycles of alkali freeze cycles, (f) GFRP specimens subjected to 100 cycles of alkali freeze cycles, (g) AFRP specimens subjected to 50 cycles of alkali freeze cycles, (h) AFRP specimens subjected to 100 cycles of alkali freeze cycles.

In order to investigate the degree of damage to the cylindrical specimen caused by the coupled alkali-freeze-thaw cycle erosion environment, the specimen’s cross-section was observed by a digital microscope with an observation factor of 100. Fig 8(D) shows the specimen’s sampling points. The fiber cloth reinforced specimen with 50 and 100 times alkali-freeze-thaw cyclic coupled erosion and the cylinder specimen without fiber cloth reinforced were selected for observation and comparative analysis, and the specimen’s microstructure was shown in Figs 18 and 19. Under 100 times of alkali-freeze-thaw cyclic coupling erosion, the cross-section microstructure of the unreinforced specimen is shown in Fig 18. PAL1-F100 and PAL1-F50 are the positions near the outer edge point 1 on the cross-section, and the coupling erosion is the most serious. The specimen is attached with a large amount of white material (alkali-silica gel). Obvious cracks were observed in PAL2-F50 and PAL2-F100. PAL3-F50 and PAL3-F100 are the central areas of the specimens where micro-cracks can be observed.

Fig 19(A)–19(H) show the microstructure of FRP-reinforced specimens under alkali-freeze-thaw cyclic erosion. Fig 19(a), 19(B), 19(G) and 19(H) show the microscopic morphology of the cross-section of the reinforced specimens with carbon fiber and aramid fiber, respectively. No apparent damage was observed under the microscope, and no micro-cracks and white alkali-silica gel were found in the cross-section of the reinforced specimens. Fig 19(C) and 19(E) show the microscopic morphology of basalt fiber and glass fiber under 50 cycles of acid-freeze-thaw erosion, respectively. It can be seen from the microscopic imaging at 1 point of cross section that the coupling erosion results in the separation of epoxy resin adhesive in the FRP strip area from the concrete matrix and the appearance of holes, thus opening a channel for the intrusion of sodium hydroxide solution and resulting in internal deterioration of concrete damage. The fiber cloth did not have apparent damage, such as breakage and wire drawing. Cracks were observed in BCAL2-F100, while GCAL2-F100 showed cracks in ITZ, the interface transition zone between aggregate and cement, the weakest concrete area. Compared with the unreinforced specimens, due to the encapsulation of epoxy resin and fiber cloth, no apparent cracks, holes, or other deterioration damage were found at cross-section point 3. From the appearance and development of microcracks, the degree of damage to the specimen is consistent with the law of acid-freeze-thaw cyclic coupling erosion. The damage of unreinforced specimens was the most serious, followed by glass fiber and basalt fiber reinforced specimens, and the damage of carbon fiber and aramid fiber reinforced specimens was the least.

**Fig 20:**
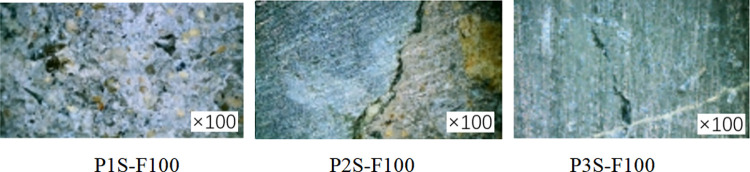
Microstructure of cross section of unreinforced specimen.

**Fig 21:**
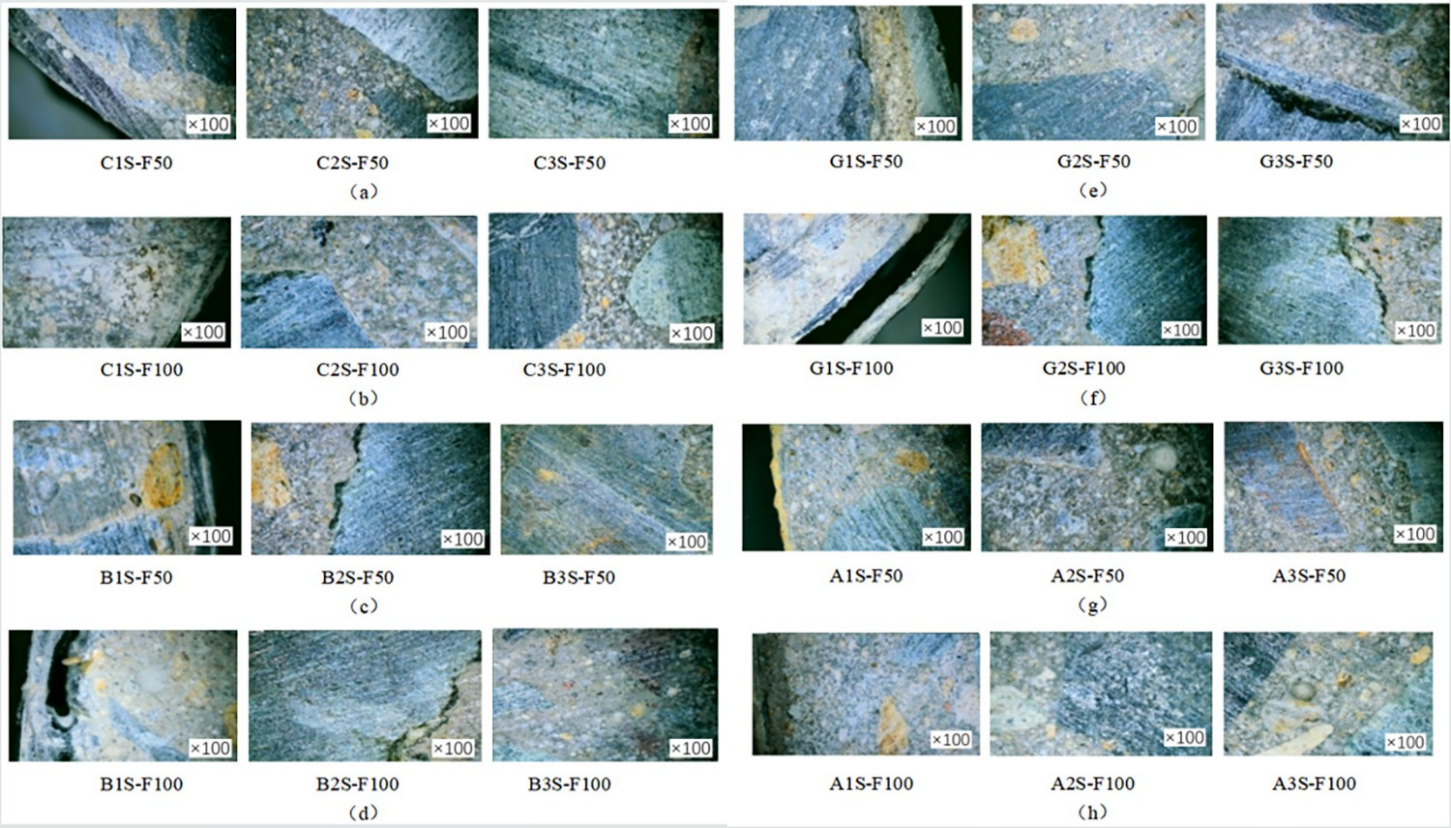
Microstructure of the FRP reinforced specimen: (a) CFRP reinforced specimens subjected to 50 cycles of salt freeze cycles, (b) CFRP specimens subjected to 100 cycles of salt freeze cycles; (c) BFRP specimens subjected to 50 cycles of salt freeze cycles, (d) BFRP specimens subjected to 100 cycles of salt freeze cycles, (e) GFRP specimens subjected to 50 cycles of salt freeze cycles, (f) GFRP specimens subjected to 100 cycles of salt freeze cycles, (g) AFRP specimens subjected to 50 cycles of salt freeze cycles, (h) AFRP specimens subjected to 100 cycles of salt freeze cycles.

In order to investigate the damage to the concrete specimen caused by the coupled salt-freeze-thaw cycle erosion environment, the specimen was cut at 1/2 height. The surface of the specimen was washed and dried, and the specimen’s cross-section was observed by a digital microscope with an observation factor of 100. Fig 8(D) shows the specimen’s sampling point. The fiber cloth reinforced specimens with 50 and 100 times acid-freeze coupling erosion and the axial compression specimen without fiber cloth reinforcement were selected for observation and comparative analysis, and the micromorphology of the specimens is shown in Figs 20 and 21. Under the action of 100 coupled salt-freeze-thaw cycles, the cross-section microstructure of the unreinforced specimen is shown in Fig 20. The position of P1S-F100 near the outer edge point 1 on the cross-section suffers the most severe coupling erosion, and the specimen is attached with many white sodium chloride crystals. P2S-F100 is the location of observation point 2, and the cross-section of P2S-F100 shows that deep cracks are generated and appear in the ITZ region of the interface between aggregate and cement paste. The position of observation point 3 is shown in figure P3S-F100, and a small crack is generated under coupling erosion.

Fig 21(A)–21(H) show the microstructure of FRP-reinforced specimens under salt-freeze-thaw cyclic erosion. Fig 21(A), 21(B), 21(G) and 21(H) show the microscopic morphology of the cross-section of the reinforced specimens of carbon fiber and aramid fiber, respectively. No apparent damage was observed under the microscope, and no micro-cracks and holes were found in the cross-section of the reinforced specimens. Figure A1S-F100 shows a small gap between the aramid fiber and the concrete matrix. Fig 21(C) and 21(E) show the microscopic morphology of basalt fiber and glass fiber under 50 cycles of salt-freeze-thaw erosion. It can be seen from the microscopic imaging at 1 point of cross section that the epoxy resin is detached from the concrete matrix, resulting in holes and damage. With the increase of coupling erosion period, the damage was more serious, but no apparent damage such as fracture and wire drawing was found. Cracks appeared in B2-F100 and G2-F100, and the crack propagation was evident with the increase of the coupling erosion period, as shown in Fig 21(D) and 21(F). Compared with the unreinforced specimens, due to the encapsulation of epoxy resin and fiber cloth, no apparent cracks, holes, or other deterioration damage were found at cross-section point 3.

The damage degree of the specimens is from large to small, and the unreinforced specimens have the most severe damage, and cracks have appeared in the central area of concrete. Glass fiber, basalt fiber, and aramid fiber reinforced specimens were the second carbon fiber and reinforced specimens had the minor damage degree. FRP reinforcement can effectively improve the durability of concrete in a salt-freeze-thaw coupling environment, and carbon fiber has the best protection effect on concrete, followed by aramid fiber, basalt fiber, and glass fiber. In summary, FRP reinforcement can effectively improve concrete’s durability in the coupled chemical-freeze-thaw cycle environment. Carbon fiber and aramid fiber have the best protection effect on concrete, followed by basalt fiber and glass fiber.

### Failure modes of epoxy and FRP sheets

**Fig 22:**
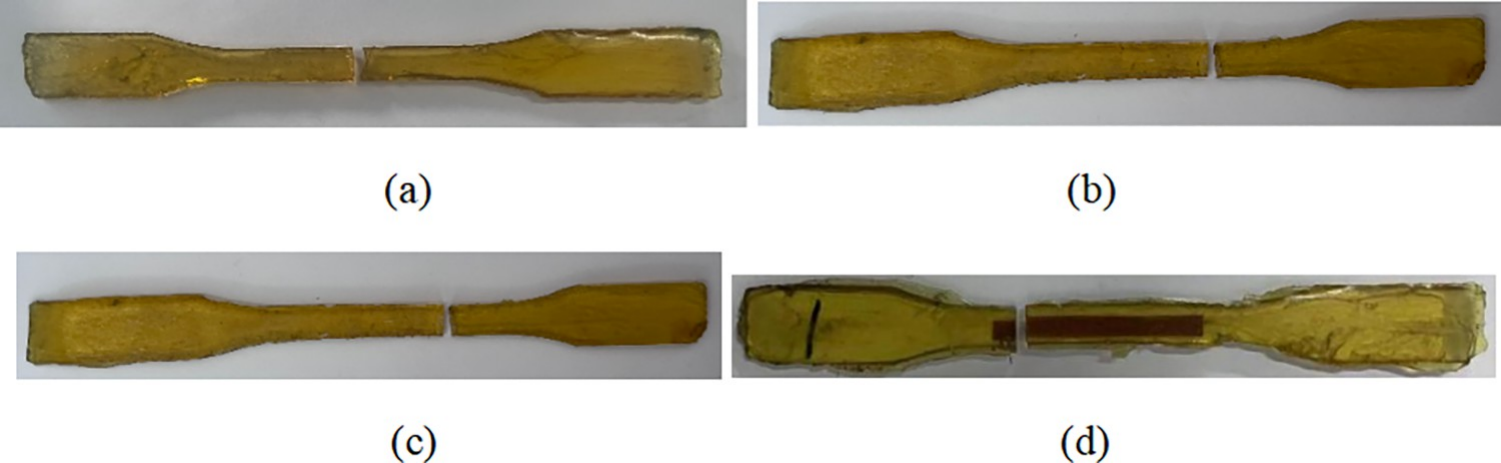
Damage pattern of epoxy resin:(a) EPN-F0, (b) EPAC-F100, (c)EPAL-F100, (d) EPSA-F100.

Fig 22(A)–22(D) show the failure modes of epoxy resin sheet EPN, EPAC-F100, EPAL-100, and EPS-F100, respectively, under the coupled acid-freeze-thaw cycles. There was no apparent damage to the sheet at the initial loading stage. When the loading strength reached the ultimate load, the specimen was broken and made a "crack" sound. The fracture position of the epoxy resin film appears in the middle zone fracture (EPN-F0), and the failure plane is perpendicular to the direction of the load. With the increase of the chemical coupling erosion period, the failure location becomes uncertain, and the failure surface tilts with the load direction.

**Fig 23:**
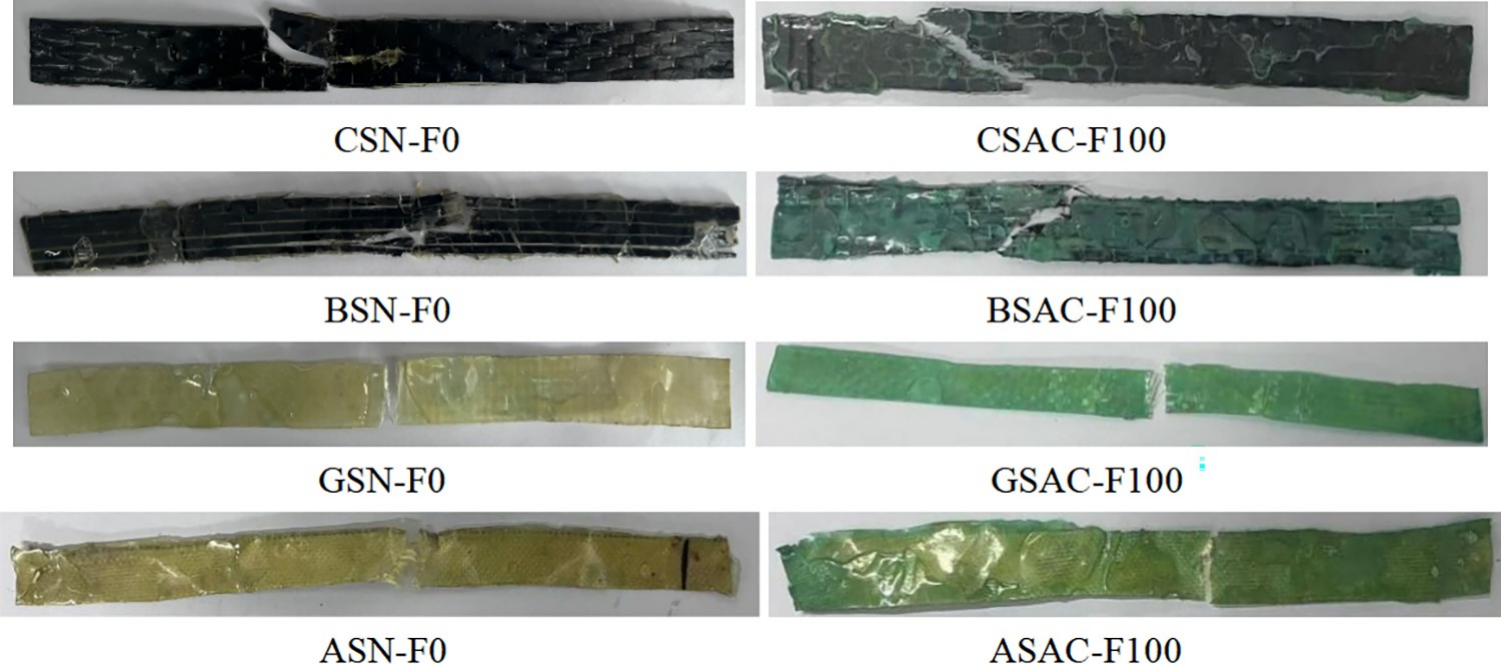
Damage patterns of FRP sheets under acid-freeze coupling erosion.

CSAC-F100, BSAC-F100, GSAC-F100, and ASAC-F100 are the same failure patterns. There was no apparent damage to the sheet at the initial loading stage. The epoxy resin matrix broke as the loading continued, and the sheet made a slight and continuous "snap" sound. When the ultimate load is reached, the sheet makes a vast "snap" sound, and the sheet breaks instantaneously, as shown in Fig 23. Carbon fiber and basalt fiber sheets start to fracture from the corner of the standard distance segment, which is because the fixture obstructs the transverse deformation at the corner of the sheet, the stress concentration at the corner leads to the sheet fracture, and the coupled erosion environment does not affect the failure form of the sheet. The aramid fiber and the glass fiber sheet are broken in the middle. It can be seen from the failure morphology of these specimens that the interface properties between fiber and epoxy resin are affected by coupling erosion, and the failure location is always in the weakened region of the fracture interface.

**Fig 24:**
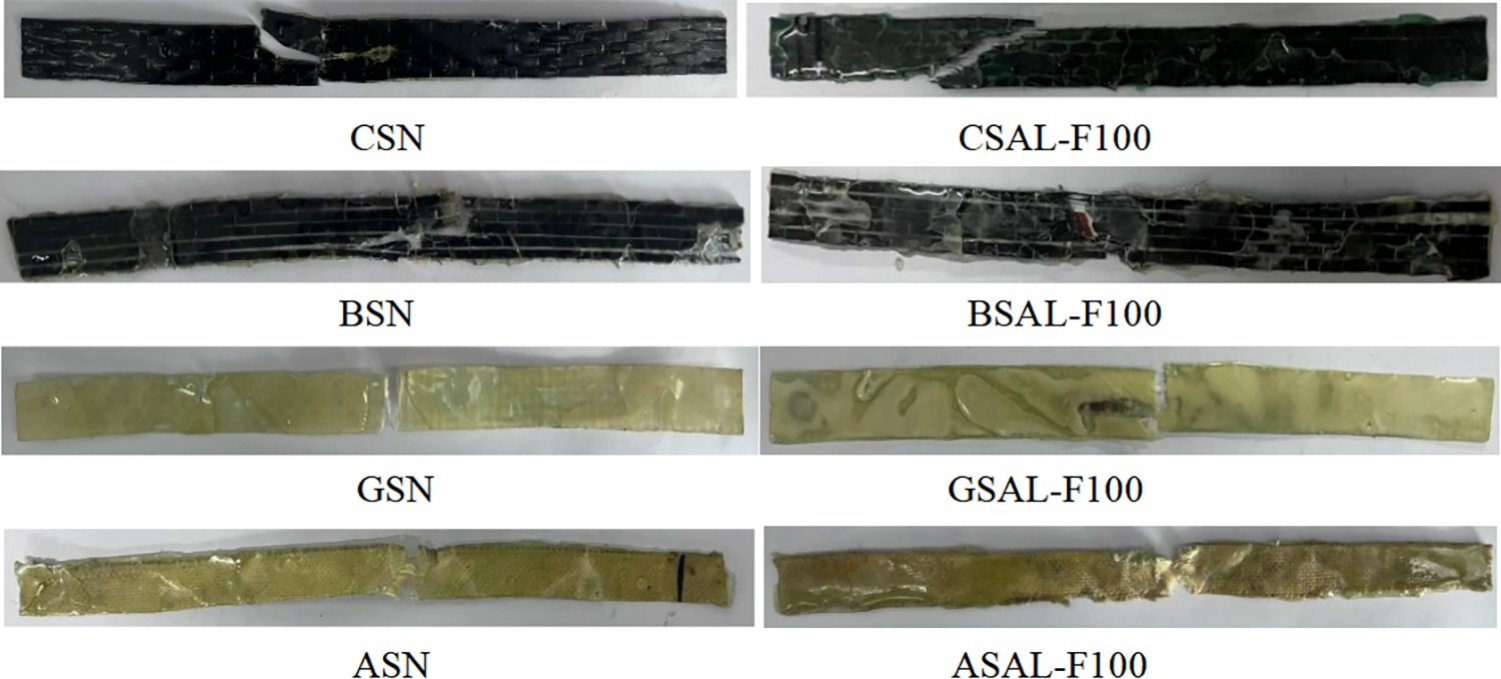
Damage patterns of FRP sheets under alkali-freeze coupling erosion.

As shown in Fig 24, the failure pattern of the sheet without alkali-freeze coupling erosion is the same as that after 100 freeze-thaw cycles. Carbon fiber (CSAL-F100) and basalt fiber sheet (BSAL-F100) start to fracture from the corner of the standard distance segment, which is because the fixture obstructs the transverse deformation at the corner of the sheet, and the stress concentration at the corner leads to the sheet fracture. The coupled erosion environment does not affect the failure form of the sheet. The aramid fiber and the glass fiber sheet are broken in the middle. It can be seen from the failure morphology of these specimens that the interface properties between fiber and epoxy resin are affected by coupling erosion, and the failure location is always in the weakened region of the fracture interface.

**Fig 25:**
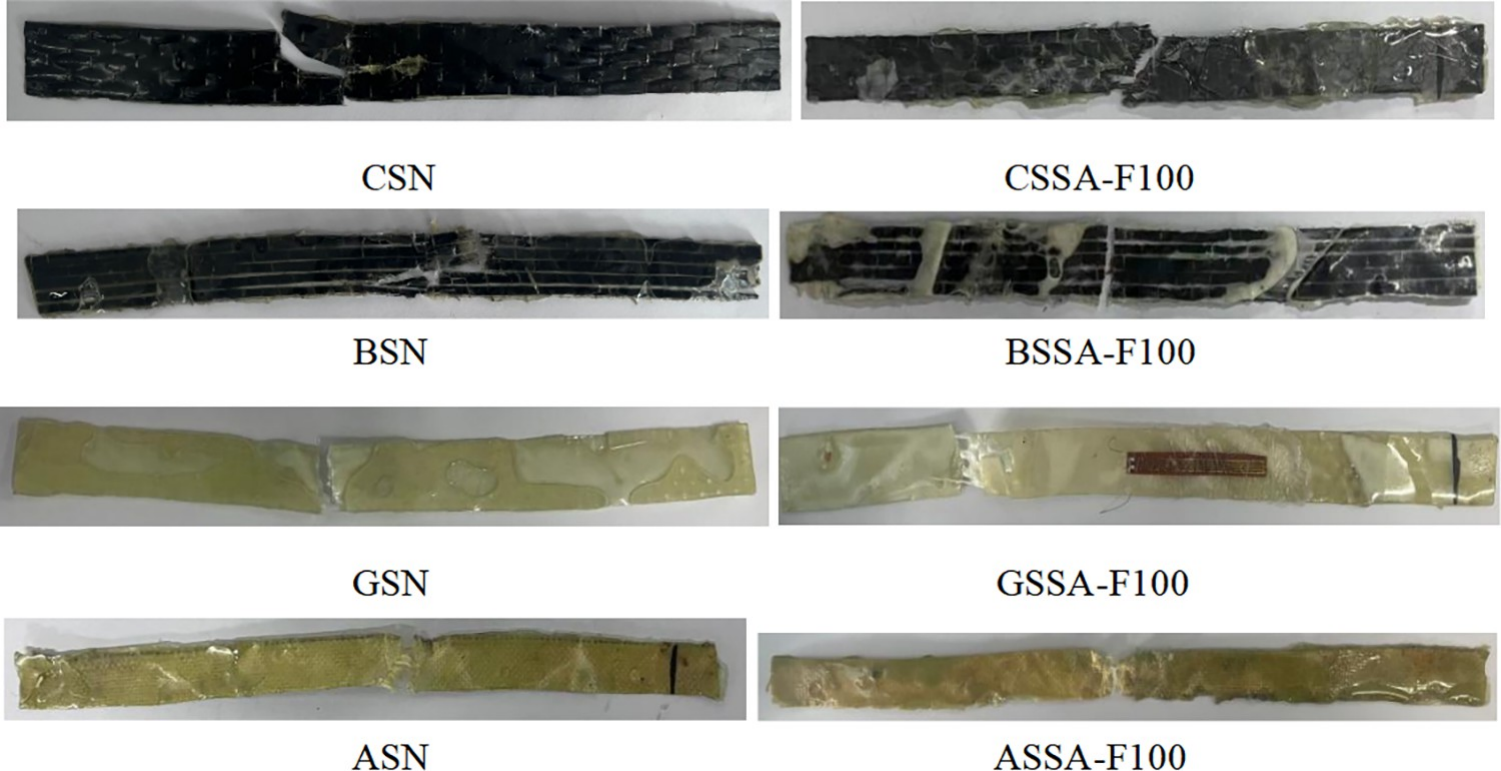
Damage patterns of FRP sheets under salt-freeze coupling erosion.

As shown in Fig 25, the failure pattern of the sheet without salt-freeze coupling erosion is the same as that after 100 freeze-thaw cycles. The carbon fiber (CSS-F100) starts to break from the corner of the standard distance segment because the fixture obstructs the transverse deformation at the corner of the sheet, and the stress concentration at the corner leads to the sheet fracture. The coupled erosion environment does not affect the failure form of the sheet. The aramid fiber (ASS-F100) and glass fiber (GSS-F100) sheets are broken in the middle. It can be seen from the failure patterns of these specimens that the interface properties between fiber and epoxy resin are affected by coupling erosion, and the failure location occurs in the weakened region of the fracture interface. To sum up. The failure pattern of the FRP sheet without chemical-freeze coupling erosion is the same as that after 100 chemical-freeze thaw cycles.

### Failure pattern analysis of cylindrical specimen

**Fig 26:**
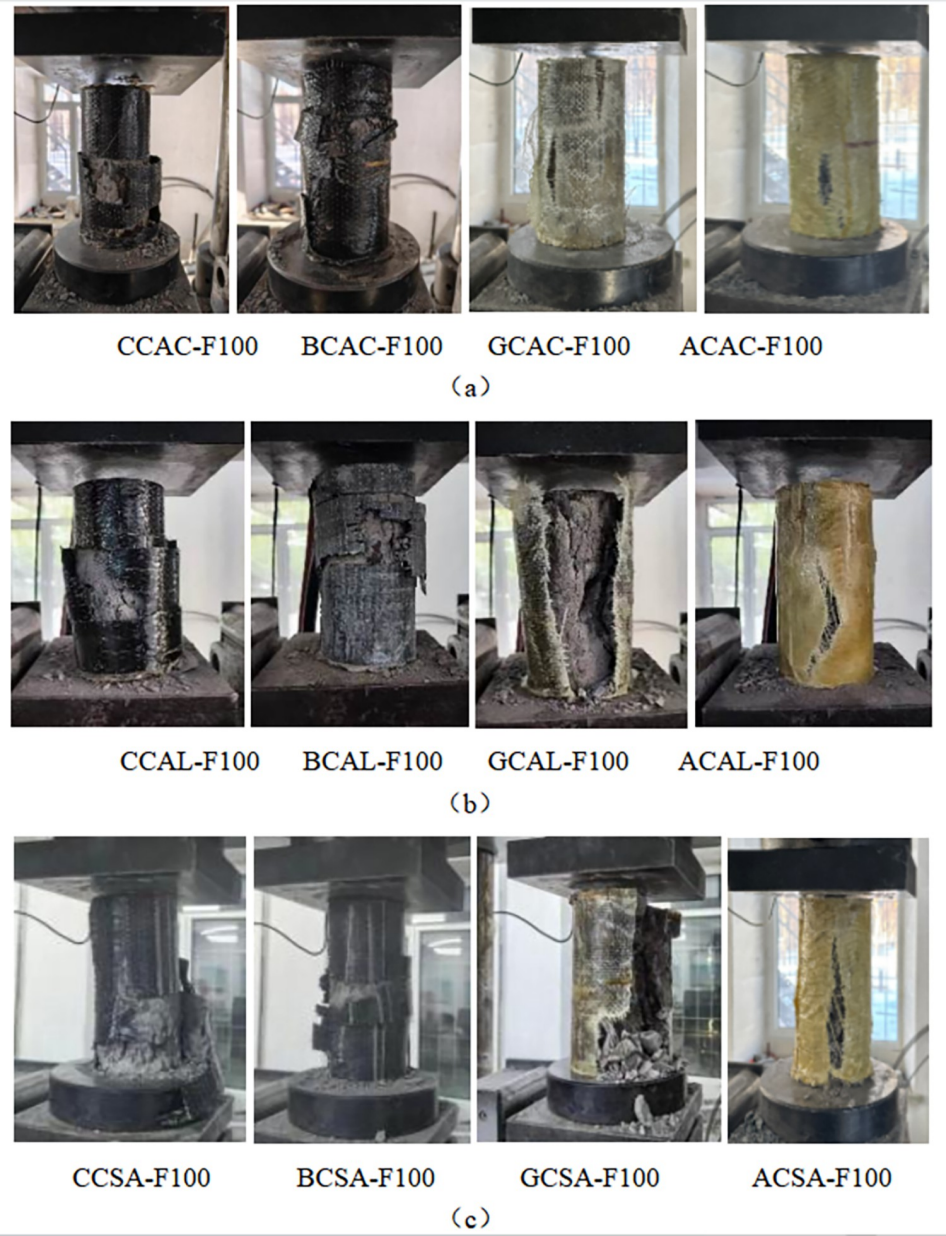
Compressive failure modes of FRP reinforced specimens and unreinforced specimens: (a) Under acid freezing coupled erosion conditions, (b) Under alkaline freezing coupled erosion conditions, (c) Under salt freezing coupled erosion conditions.

Fig 26 shows the typical compressive failure patterns of FRP-reinforced and unreinforced specimens. It can be seen that the carbon fiber and basalt fiber reinforced specimens show annular cracking in the middle. At the initial loading stage, the specimen had no noticeable change. When the load gradually increased, the specimen made a continuous and slight "crackling" sound, and part of the epoxy resin matrix cracked. When the load reached the ultimate bearing capacity of the specimen, the specimen made a vast "crackling" sound, and the middle part of the FRP strip broke instantaneously, accompanied by concrete splashes. The specimen showed apparent brittle failure, and the morphology of the damaged specimen is shown in Fig 26(A) and 26(B). With the increase of pressure, the friction at both ends increases, the swelling in the middle will become larger and larger, and the final specimen will be destroyed first from the middle part.

In the three chemical erosion environments, the failure of AFRP reinforced specimens showed strong conductivity, and the fiber cloth at the failure site gradually cracked, which was different from the brittle failure of other FRP and was related to the densification of the fiber cloth itself, as shown in ACC-F100, ACAL-F100, and ACSA-F100. For glass fiber reinforced cylindrical specimens, the degradation phenomenon in chemical solution is evident; the color becomes bright, and the damage diagram confirms this view. The failure of the control group was manifested as concrete fragmentation because there was no strengthening of FRP itself, and it was affected by the coupling effect of the acid-freeze-thaw cycle.

### Failure pattern analysis of prismatic specimen

For unreinforced prismatic specimens with different coupled erosion cycles of chemical-freeze-thaw cycles, their failure patterns are the same, and there is no noticeable change in the specimens at the initial loading stage. With the progress of loading, tiny cracks appear at the prefabricated cracks of the specimens, and then the cracks rapidly expand upward to the top of the specimens. The failure pattern is the bending failure of the regular section. The typical failure for FRP-reinforced concrete bending specimens is that the FRP strip is spalling from the surface of the concrete, and the regular section of the concrete is bending.

**Fig 27:**
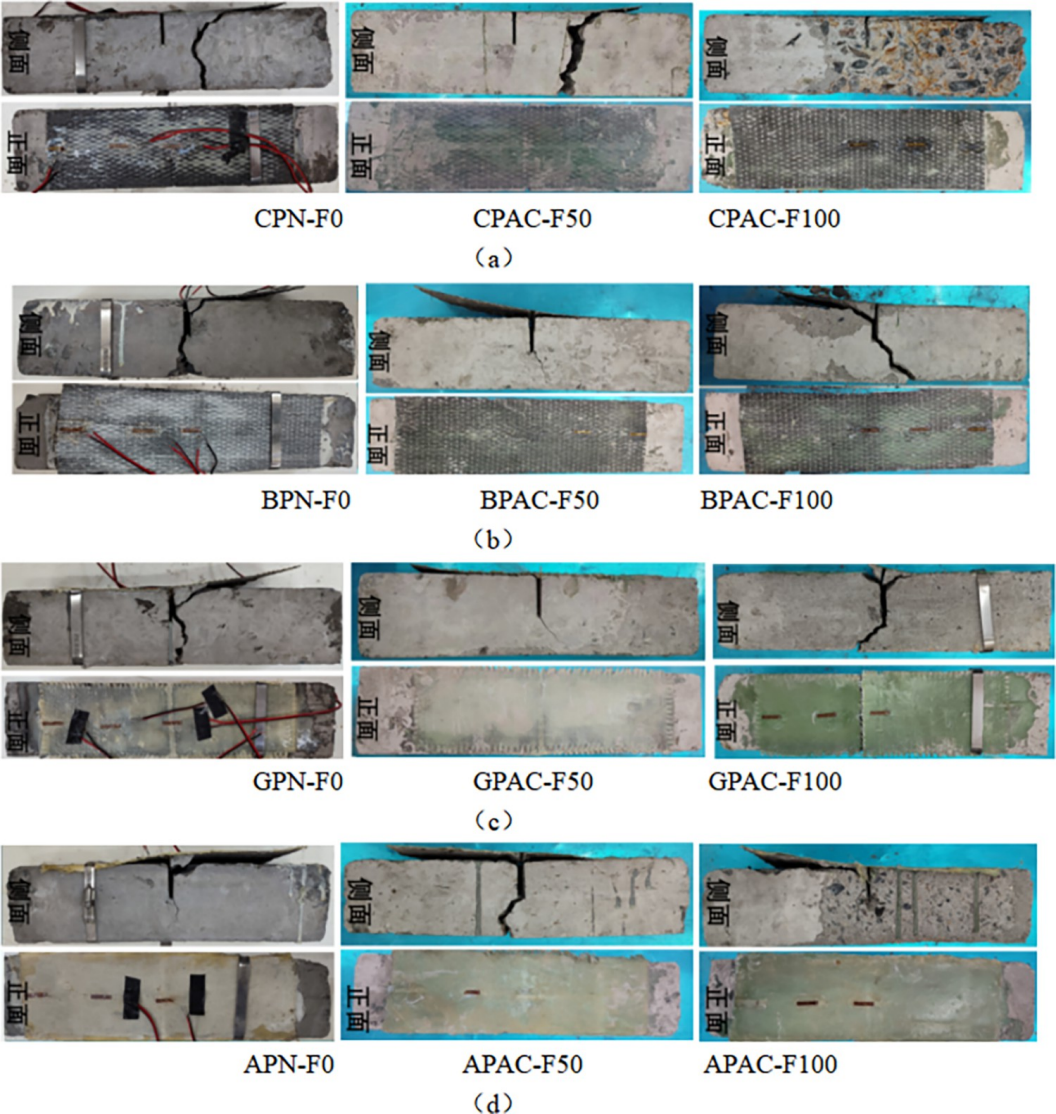
Failure modes of prismatic specimen under acid-freeze coupling erosion: (a) Failure modes of Carbon fiber reinforced prismatic specimen; (b) Failure modes of basalt fiber reinforced prismatic specimen; (c) Failure modes of glass fiber reinforced prismatic specimen; (d) Failure modes of aramid fiber reinforced prismatic specimen.

Fig 27 shows the failure morphology of the acid-freeze coupling erosion prismatic specimen. The failure forms are basically the same for different kinds of reinforced bending specimens. FRP fracture of specimen GPAC-F100. After 100 times of acid-freeze coupling erosion, GFRP is aged so that its tensile strength is lower than the bond tension between the concrete and FRP interface. A large surface concrete of the GPAC-F100 specimen spalled off, and the bond between FRP and concrete decreased. For unreinforced flexural specimens, the concrete on the upper side was compressed, while the concrete on the lower side was strained. As the tensile strength of concrete was far less than the compressive strength, cracks appeared at the precast cracks and extended.

**Fig 28:**
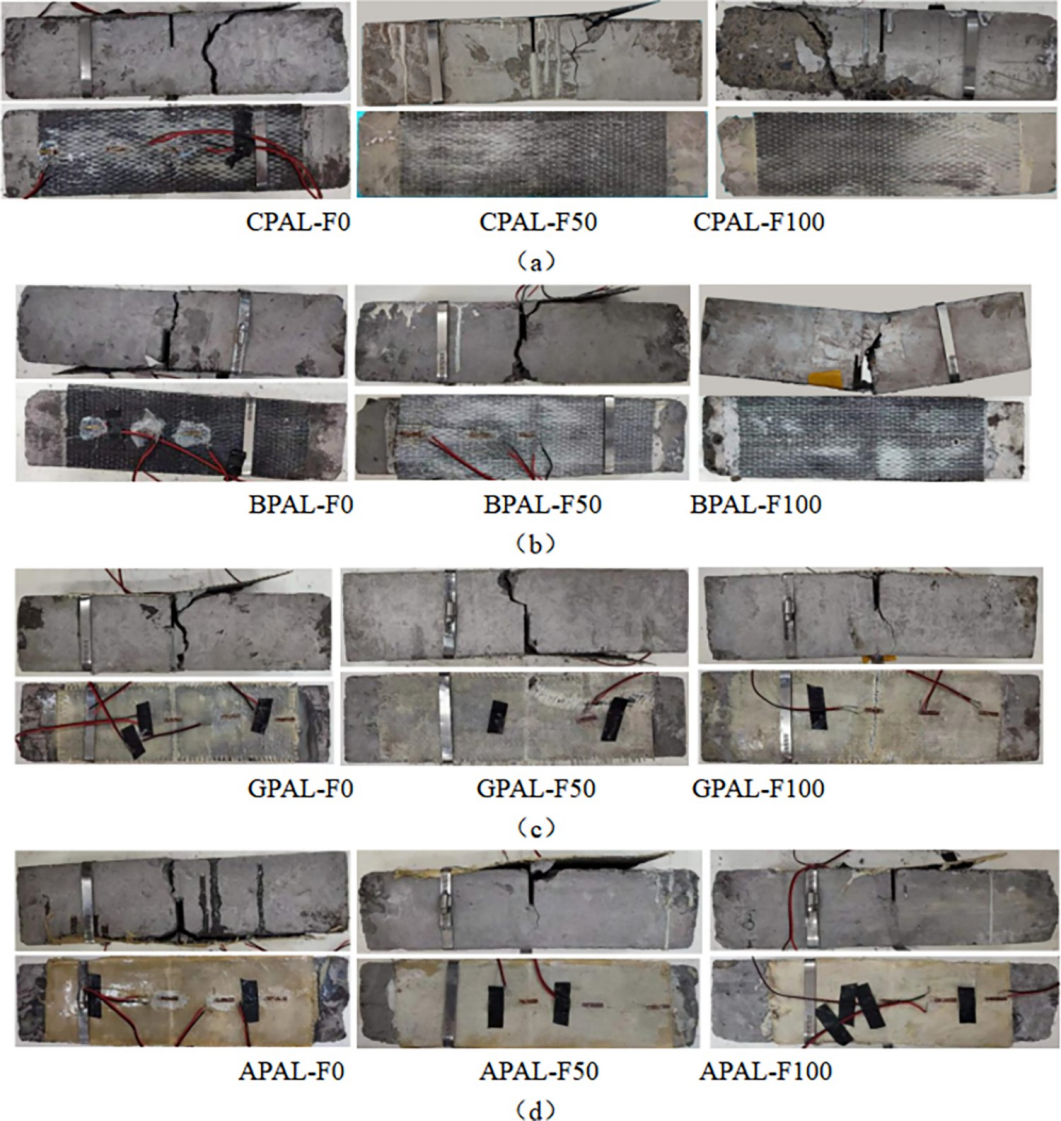
Flexural failure patterns of FRP reinforced specimens and unreinforced specimens under alkali-freeze coupling erosion (a) Failure modes of Carbon fiber reinforced prismatic specimen; (b) Failure modes of basalt fiber reinforced prismatic specimen; (c) Failure modes of glass fiber reinforced prismatic specimen; (d) Failure modes of aramid fiber reinforced prismatic specimen.

**Fig 29:**
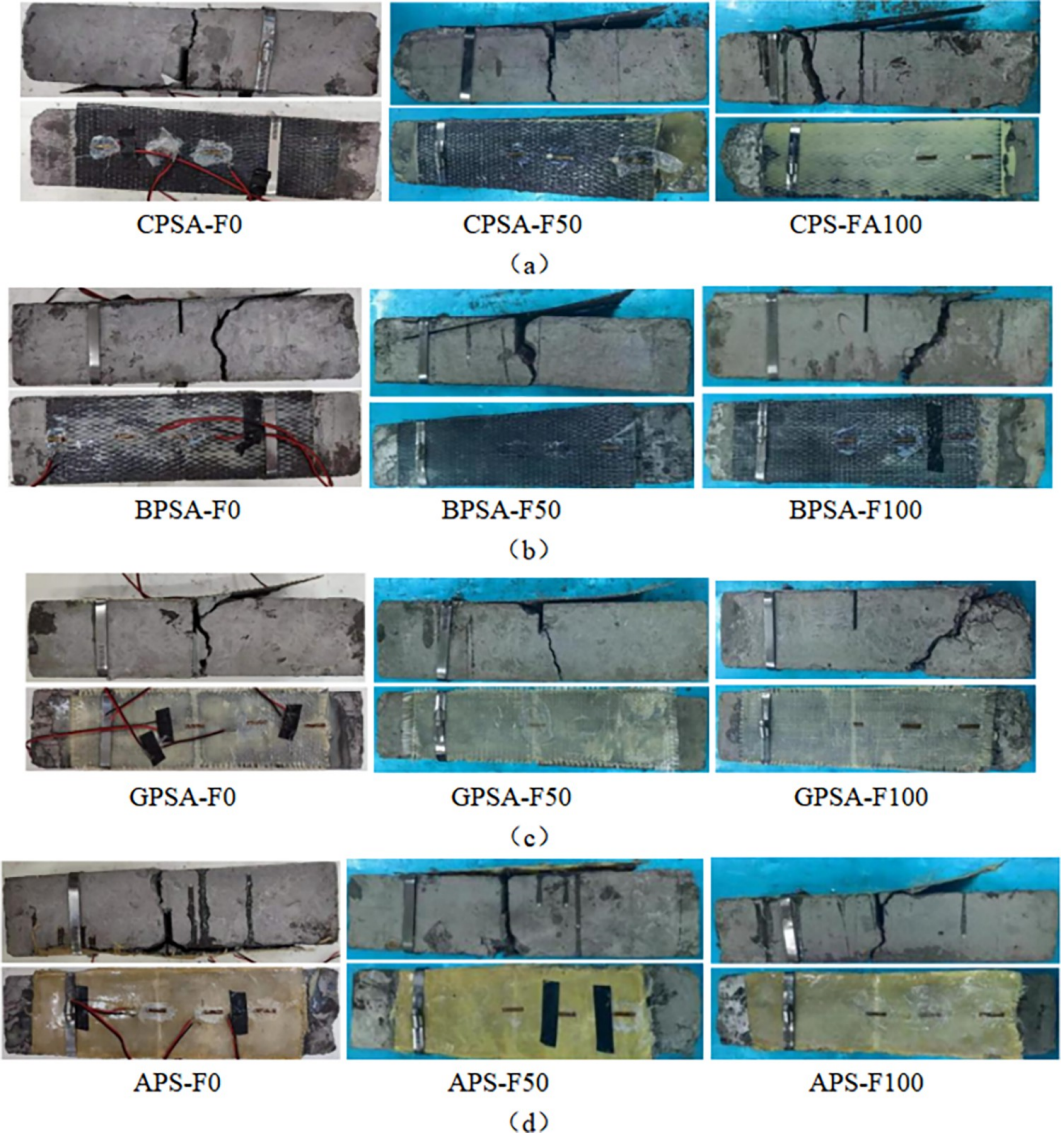
Flexural failure patterns of FRP reinforced specimens and unreinforced specimens under salt-freeze coupling erosion (a) Failure modes of Carbon fiber reinforced prismatic specimen; (b) Failure modes of basalt fiber reinforced prismatic specimen; (c) Failure modes of glass fiber reinforced prismatic specimen; (d) Failure modes of aramid fiber reinforced prismatic specimen.

Fig 28 shows the failure morphology of the alkali-freeze coupling erosion prism specimen. For specimens BPAL-F100 and GPAL-F100, the FRP of the specimens was broken. After 100 times of alkali-freeze coupling erosion, BFRP and GFRP are fractured and destroyed, and their failure patterns are shown in Fig 28(C). Fig 29 shows the failure morphology of the salt-freeze coupling erosion prismatic specimen. With the progress of loading, a small crack appeared at the prefabricated crack of the specimen, and then the crack rapidly expanded upward and extended to the top of the specimen. The damage mode was bending failure of the standard section, as shown in Fig 29. The failure forms are different for different kinds of reinforced bending specimens. For specimens BPSA-F100 and GPSA-F100, the failure mode of specimens is the concrete oblique section failure, and the upper and lower ends of the oblique crack are near the loading point and the support, respectively. The bonding surface between the BFRP strip and the GFRP strip and concrete is not damaged, as shown in Fig 29(B) and 29(C).

In summary, in different chemical and frost-coupled erosion environments, FRP-reinforced concrete mainly presents typical FRP strips peeling off the concrete surface and bending damage to the standard concrete section. After the chemical-freeze-thaw cycle coupling, the bonding force between FRP and concrete is reduced, the strengthening effect is weakened, and the FRP of GPAC-F100, BPAL-F100, and GPAL-F100 are fractured.

## Mechanical test result

### FRP sheet and epoxy resin

**Fig 30:**
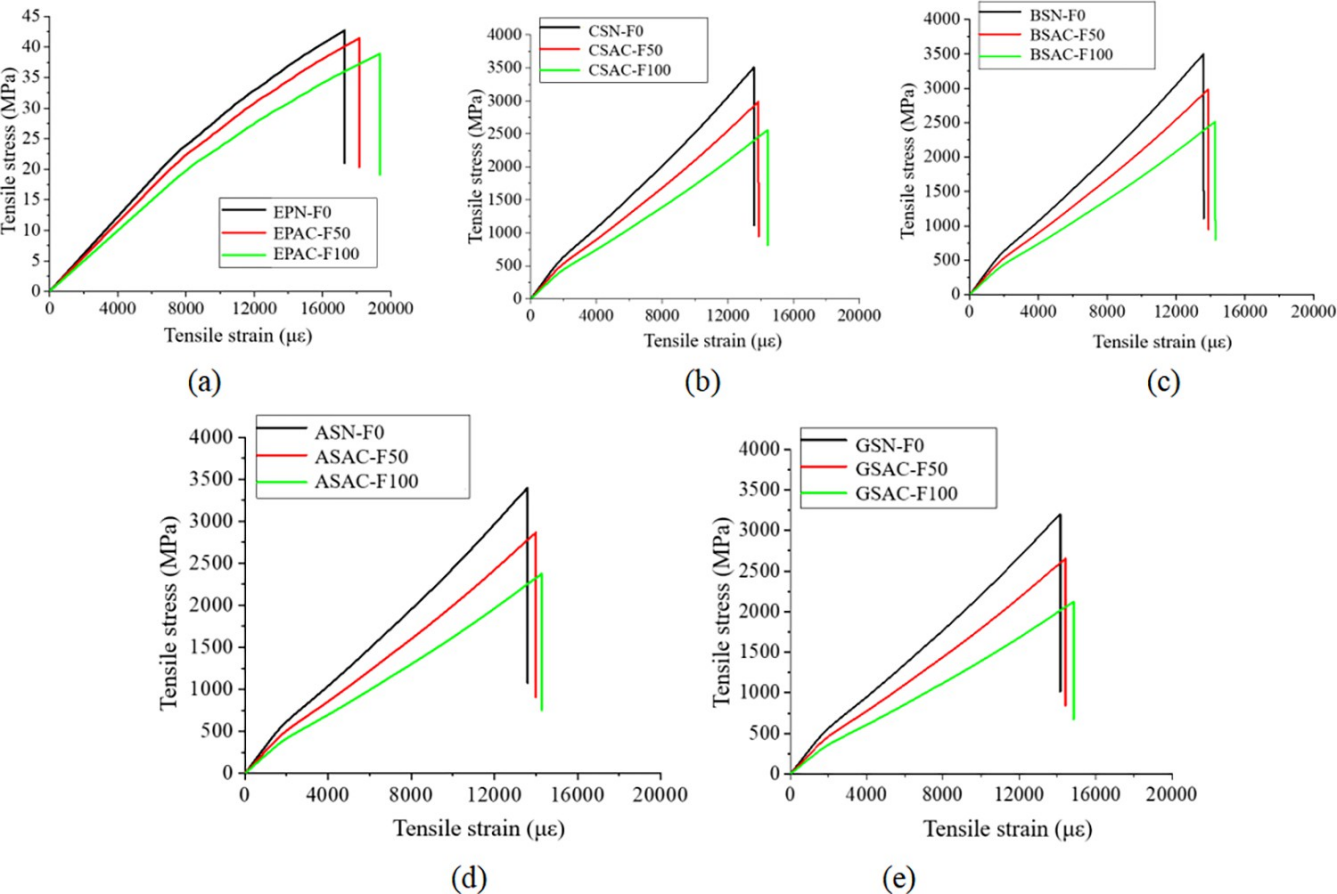
Stress-strain curve of fiber sheet in coupled acid-freeze-thaw cycle environment: (a) epoxy resin, (b) CFRP sheet, (c)BFRP sheet, (d) AFRP sheet, (e) GFRP sheet.

**Fig 31:**
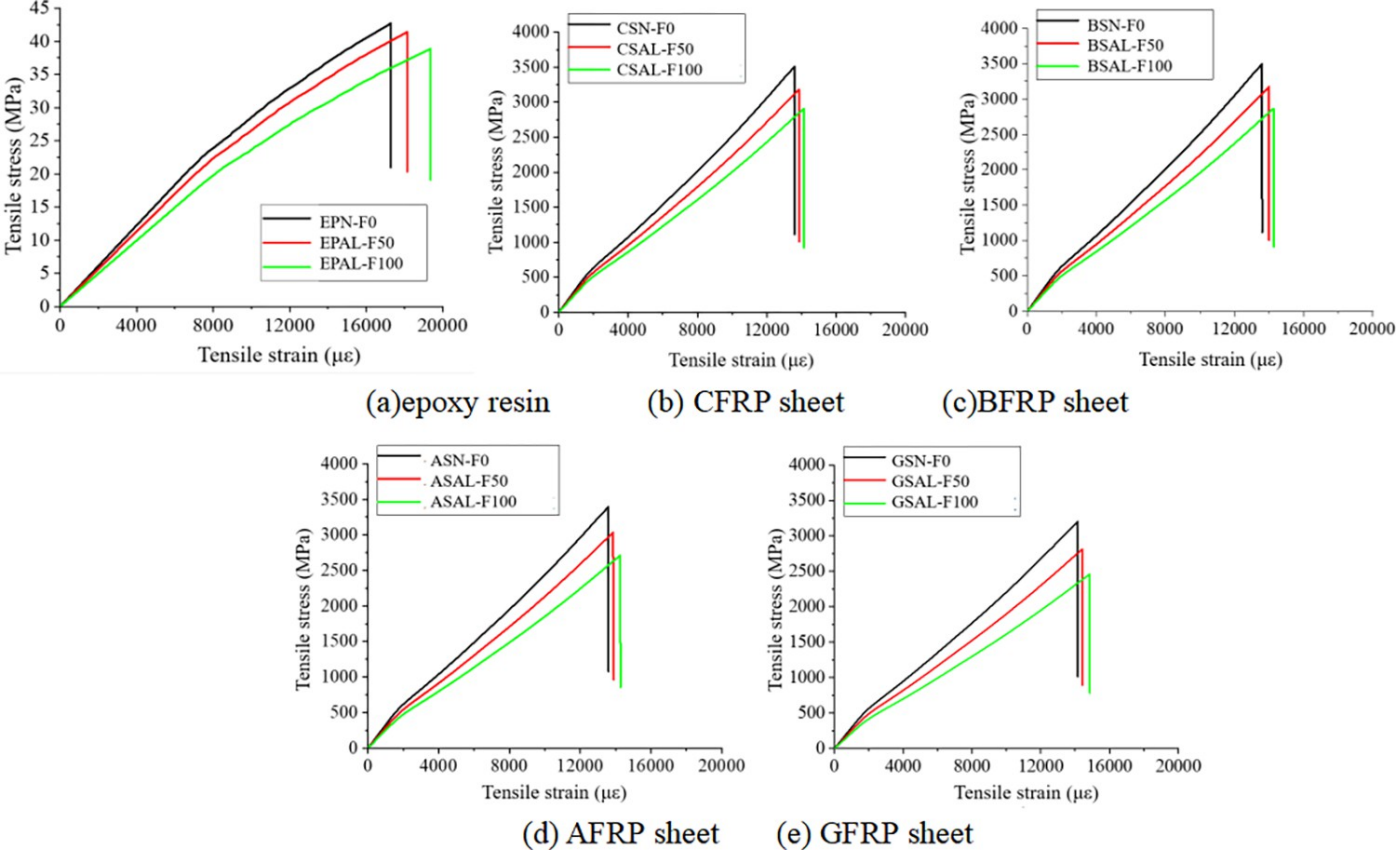
Stress-strain curve of sheet under alkali-freeze-thaw cyclic coupling environment: (a) epoxy resin, (b) CFRP sheet, (c)BFRP sheet, (d) AFRP sheet, (e) GFRP sheet.

**Fig 32:**
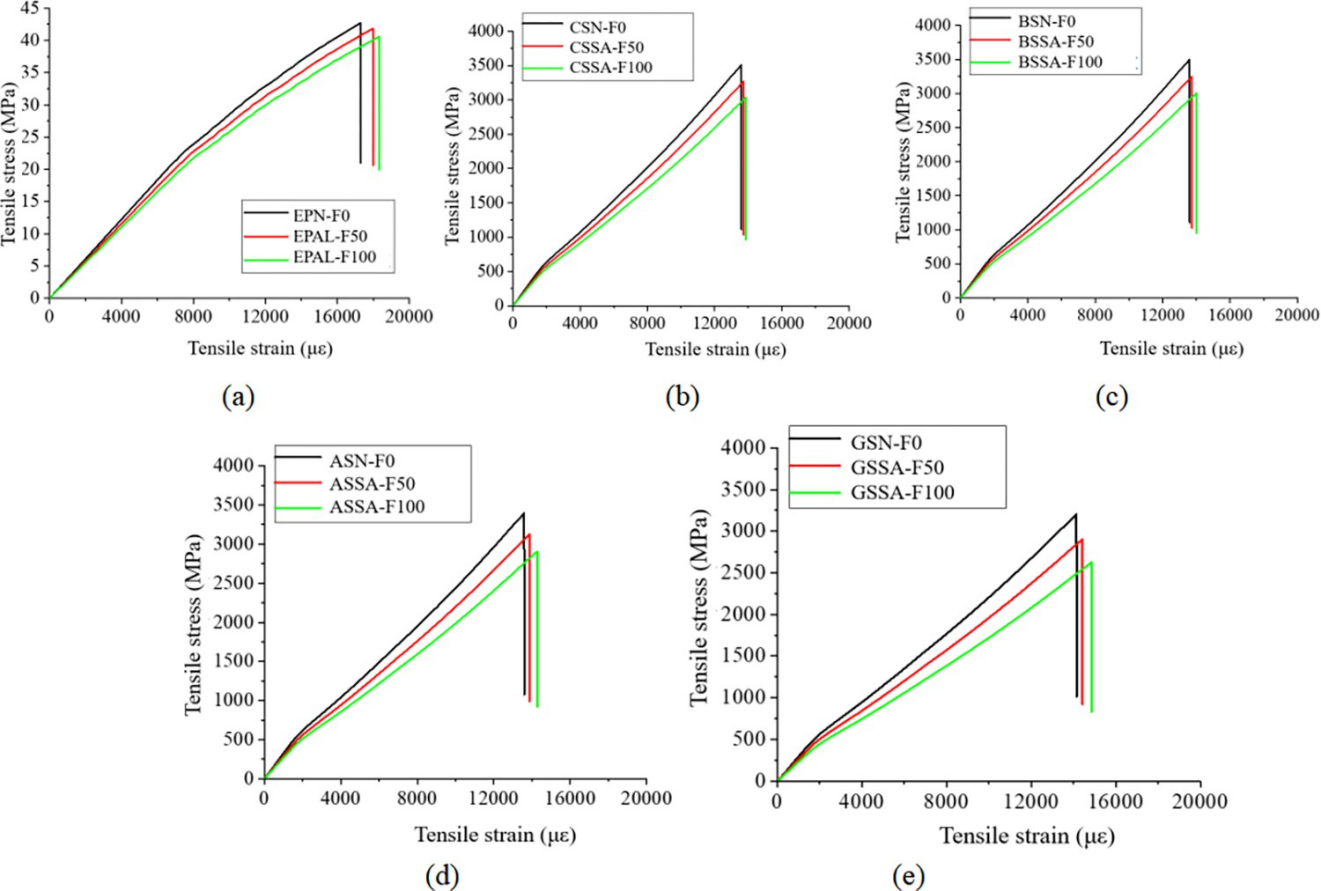
Stress-strain curves of epoxy resin and FRP sheet in coupled salt-freeze-thaw cycle environment: (a) epoxy resin, (b) CFRP sheet, (c)BFRP sheet, (d) AFRP sheet, (e) GFRP sheet.

Figs 30(A), 31(A) and 32(A plot the stress-strain curves of each sheet under the coupled chemistry-freeze-thaw cycle erosion environment. In the acid-freeze environment, the ultimate tensile strength of epoxy resin adhesive was measured to decrease by 9.02% when the acid-freeze-thaw cycle was 50 times and 13.57% when the acid-freeze-thaw cycle was 100 times. The ultimate tensile strength of epoxy resin adhesive was measured by coupling erosion of 50 times and 100 times in alkali-freeze-thaw cycles, and the tensile strength decreased by 7.01% and 11.79%, respectively. The tensile strength decreased by 6.42% and 11.12% under 50 and 100 cycles of coupled salt-freeze-thaw erosion, respectively. In the coupled erosion environment, the tensile strength of epoxy resin film decreases, the ultimate tensile strain increases, the elastic modulus decreases, and the second half of the stress-strain curve shows nonlinearity, which indicates that the mechanical properties of epoxy resin film are seriously degraded. This is because the epoxy resin film, due to the hydrolysis of groups, chloride ions, and freeze-thaw cycle damage caused by surface and internal damage (micro-cracks), is the main factor of the degradation of the mechanical properties of the sheet.

Fig 30(B)–30(E) show each FRP sheet’s stress-strain curves under the coupled acid-freeze-thaw cycle erosion environment. It can be observed that the stress-strain curves of various FRP sheets are highly similar, exhibiting linear elasticity. After reaching the maximum tensile strength, their strength rapidly decreases, demonstrating a distinct brittleness. The stress-strain curve of FRP sheets differs slightly from that of epoxy resin adhesive, which exhibits a nonlinear behavior. The same research conclusion has also been reached in the literature [35]. Although FRP materials exhibit brittleness, the overall structure reinforced with FRP through wrapping around concrete demonstrates superior ductility. This enhanced ductility is achieved through various mechanisms, including restraint effects, improved stress distribution, enhanced crack control, and increased bending resistance. The ultimate tensile strength of the CFRP sheet under the coupled erosion condition is 3508.86MPa, the erosion cycles of the acid-freeze-thaw cycle are 50 and 100 times, and the tensile strength is reduced by 14.87% and 27.08%, respectively. The ultimate tensile strength of the BFRP sheet is 3497.98MPa without coupling erosion. After 50 and 100 cycles of acid-freeze-thaw erosion, the tensile strength decreases by 15.02% and 28.32%, respectively. AFRP sheets were reduced by 15.64% and 30.17%, respectively. GFRP decreased by 15.64% and 30.17%, respectively. For FRP sheets, the tensile strength of epoxy resin adhesive is much smaller than that of the fiber cloth. Hence, the fiber cloth mainly determines the mechanical properties of the sheet, and the damage to the epoxy resin adhesive matrix has little effect on the mechanical properties of the sheet. The interface damage between fiber filament and epoxy resin matrix is the main factor leading to the degradation of the mechanical properties of FRP sheets. On the one hand, the hydrolysis reaction leads to the gap between the fiber filament and the epoxy resin matrix [14]. On the other hand, the temperature expansion coefficient of fiber silk and epoxy resin adhesive is different, and the interface stress between fiber silk and epoxy resin adhesive occurs during the freeze-thaw cycle, resulting in interface damage.

Fig 31(B)-31(E) Plot the stress-strain curves of each FRP sheet under the coupled alkaline freeze-freeze-thaw cycle erosion environment. The ultimate tensile strength of the CFRP sheet is 3508.86MPa without erosion. The tensile strength decays by 9.32% and 17.12%, respectively, after 50 and 100 times of coupling erosion. The ultimate tensile strength of the BFRP sheet under non-erosion conditions is 3497.98MPa. The coupling erosion of alkali-freeze-thaw cycles occurred 50 times and 100 times, and the tensile strength attenuated at 9.57% and 18.26%, respectively. AFRP sheet decreases by 10.66% and 20.12%, respectively. The tensile strength of GFRP sheets decreases by 12.16% and 23.17%, respectively. It shows that the mechanical properties are seriously degraded under the alkali-freeze-thaw cyclic coupling erosion environment. The coupling erosion resistance is CFRP> from high to low. BFRP> AFRP> GFRP.

Fig 32(B)–32(E) plots the stress-strain curves of each FRP sheet under the coupled salt-freeze-thaw cycle erosion environment. The tensile strength of the four kinds of sheets is good. They are CFRP, BFRP, AFRP, and GFRP from high to low. The curves drawn by the tensile test data show that the early stage is elastic, the later stage is elastic-plastic, and the sudden fracture occurs when the ultimate load is reached after the continuous increase. The ultimate tensile strength of the CFRP sheet without erosion is 3508.86MPa, according to the data obtained from the tensile test of the fiber sheet. The coupling erosion of salt-freeze-thaw cycles 50 times and 100 times reduces the tensile strength by 6.87% and 13.52%, respectively. The ultimate tensile strength of the BFRP sheet is 3497.98MPa without erosion, and the tensile strength decreases by 7.47% and 14.28% after 50 and 100 salt-freeze-thaw cycles. AFRP intensity decays 7.98% and 14.41%; The ultimate tensile strength of the GFRP sheet under non-erosion conditions is 3201.41MPa, and the salt-freeze-thaw cycles of 50 and 100 times are reduced by 9.34% and 18.02%, respectively. In the coupled erosion environment, the tensile strength of FRP sheets decreases slightly, and the ultimate tensile strain increases slightly, indicating that FRP sheets’ mechanical properties are degraded. The tensile strength degradation rate of the CFRP sheet is lower than that of epoxy resin adhesive and BFRP, AFRP, and GFRP sheet, indicating that the durability of the CFRP sheet is better than that of the other four sheets under the coupled salt-freeze-thaw cycle environment.

Compared with the salt-freeze-thaw cycle and alkali-freeze-thaw cycle, the mechanical properties of the four FRP sheets are degraded most seriously under the coupled acid-freeze-thaw cycle. At the same time, the properties of the acid-freeze-thaw cycle from high to low are CFRP, BFRP, AFRP, and GFRP. The curves drawn by the tensile test data all show that the early stage is elastic, and after the strain reaches a particular stage, it presents elastic-plastic and then suddenly breaks when the limit load is reached. By comparing the stress-strain curves of each sheet under the coupled acid-freeze-thaw cycle environment, it is evident that the tensile strength loss of the CFRP sheet is smaller than that of BFRP, AFRP, and GFRP sheet, indicating that the durability of CFRP sheet under the coupled acid-freeze-thaw cycle erosion environment is better than that of the other three sheets. For the most seriously degraded GFRP sheet, the tensile degradation strength exceeds 30% after 100 acid-freeze-thaw cycles. Its strength can not meet the actual requirements of the paste. CFRP showed good durability in the three erosion environments compared with the other three fiber fabrics.

### Cylindrical specimen reinforced with FRP

**Fig 33:**
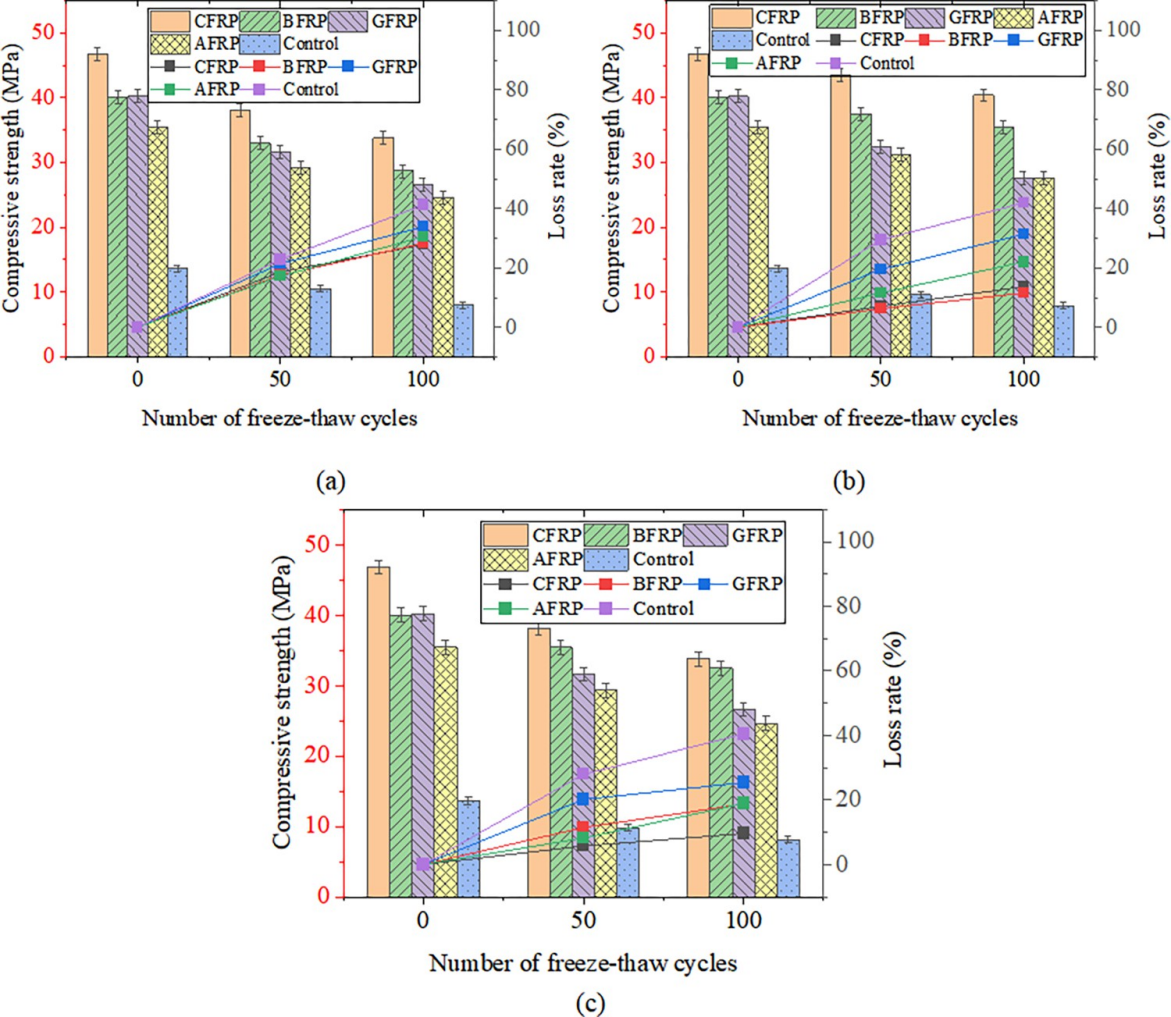
Compressive strength and strength loss rate of cylindrical specimens under different freeze-thaw cycles:(a) acid-freeze cycle erosion, (b) alkali-freeze cycle erosion, (c) salt-freeze cycle erosion.

Fig 33(A) shows FRP-reinforced specimens’ compressive strength and strength loss rate under different acid-freeze-thaw cycles. With the increase of the acid-freeze-thaw cycle, the compressive strength of each group was reduced to a certain extent. When the acid-freeze-thaw cycle was 100 times, the strength of the CFRP-reinforced specimens was the highest, 33.8kN, 4.225 times that of the control specimens. Compared with the initial strength without coupled acid-freeze-thaw cyclic erosion, the strength loss rates of CFRP reinforced specimens<BFRP reinforced specimen<AFRP Reinforced specimen<GFRP reinforced specimen<The control group are small to large. The strength loss of the reinforced and reinforced specimens after 100 acid-freeze-thaw cycles is similar, and the strength loss rate of the reinforced specimen is the lowest at 27.8%. The acid-freeze-thaw cycle of GFRP-reinforced specimens is poor, and the strength loss is 33.8%.

Fig 33(B) shows FRP-reinforced specimens’ compressive strength and strength loss rate under different alkali-freeze-thaw cycles. It can be seen that with the increase of the freeze-thaw cycle and alkali corrosion time, the compressive strength of each group is reduced to a certain extent. Compared with the initial strength of 0 freeze-thaw cycles, the strength loss rate of the CFRP reinforced specimens<AFRP Reinforced specimen< GFRP reinforced specimen<BFRP reinforced specimen<The control group from small to large. The minimum loss of 100 times of freeze-thaw cyclic corrosion of CFRP-reinforced specimens is only 7.1%, and the loss of 100 times of freeze-thaw cyclic corrosion of GFRP-reinforced specimens is very close to that of BFRP-reinforced specimens. The initial strength of AFRP-reinforced specimens is lower than that of GFRP-reinforced specimens and BFRP-reinforced specimens during freeze-thaw cyclic corrosion. However, the strength values of the three specimens are very similar after 100 times of freeze-thaw cyclic corrosion.

Fig 33(C) shows FRP-reinforced specimens’ compressive strength and strength loss rate under different salt-freeze-thaw cycles. It can be seen that the changes in compressive strength of concrete reinforced by four different fiber materials are similar. After 100 freeze-thaw cycles, the compressive strength of the concrete cylindrical specimens reinforced by CFRP,GFRP, BFRP, and AFRP decreased by 8.3%, 19.2%, 27.2%, and 15.3%, respectively. The compressive strength of the specimens strengthened with CFRP has the slightest change. Adhesive FRP significantly improves the compressive strength of specimens under chemical erosion, and the compressive strength of specimens of different groups in different environments also has the same rule, that is, CFRP reinforced specimens>BFRP reinforced specimen>GFRP Reinforced specimen>AFRP strengthens the specimen, which is related to the high strength of FRP itself. Regarding the strength loss rate of FRP-reinforced specimens, the strength loss rate of CFRP-reinforced specimens in a chemical environment is the smallest. The durability is the best, followed by the strength and durability of BFRP and AFRP specimens. The strength loss rate of GFRP-reinforced specimens is the largest, and the chemical durability is the worst.

**Fig 34:**
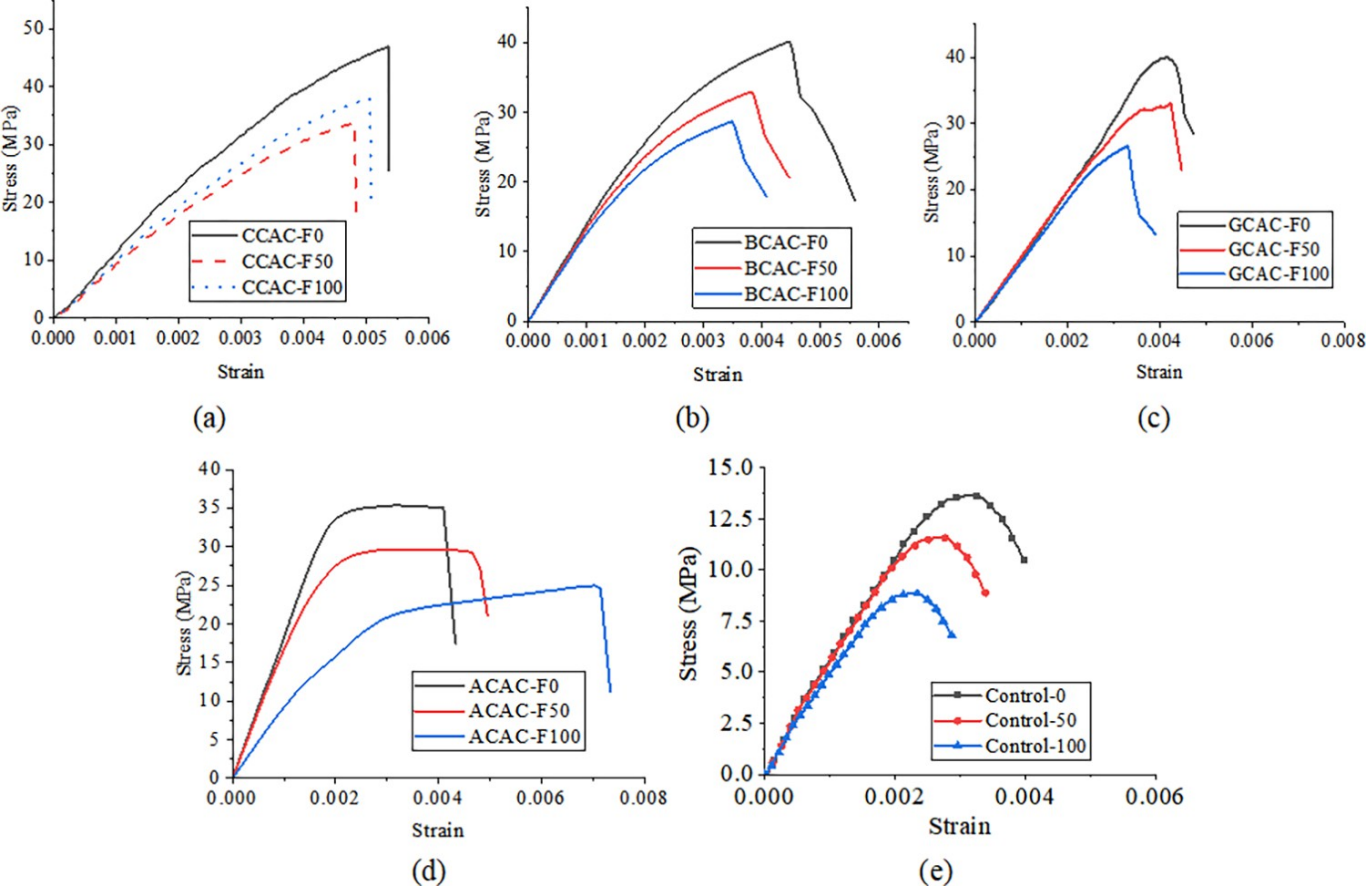
Uniaxial compressive stress-strain curve of cylindrical specimen under coupled acid-freeze erosion: (a)CFRP reinforced specimen, (b) BFRP reinforced specimen, (c) GFRP reinforced specimen, (d) AFRP reinforced specimen, (e)Control group specimen.

Fig 34 shows each group of specimens’ uniaxial compressive stress-strain curves under the action of acid-freeze coupling erosion. With the progress of coupling erosion, the ultimate bearing capacity of the specimen decreases gradually. The specimens in the control group have typical concrete stress-strain curves, but the FRP-reinforced specimens’ stress-strain curves change obviously. The curves of the specimens strengthened by CFRP and BFRP are similar. When the specimens reach the maximum compressive strength, their strength decreases rapidly, showing brittle failure. The constitutive curves of GFRP-reinforced specimens are similar to those of the control group and have a certain plasticity. However, in AFRP-reinforced specimens, when the compressive strength of specimens is close to the compressive strength, the deformation is large, the strength is slow, and the plasticity is good. The order of plasticity is AFRP>GFRP>Control group>BFRP>CFRP.

**Fig 35:**
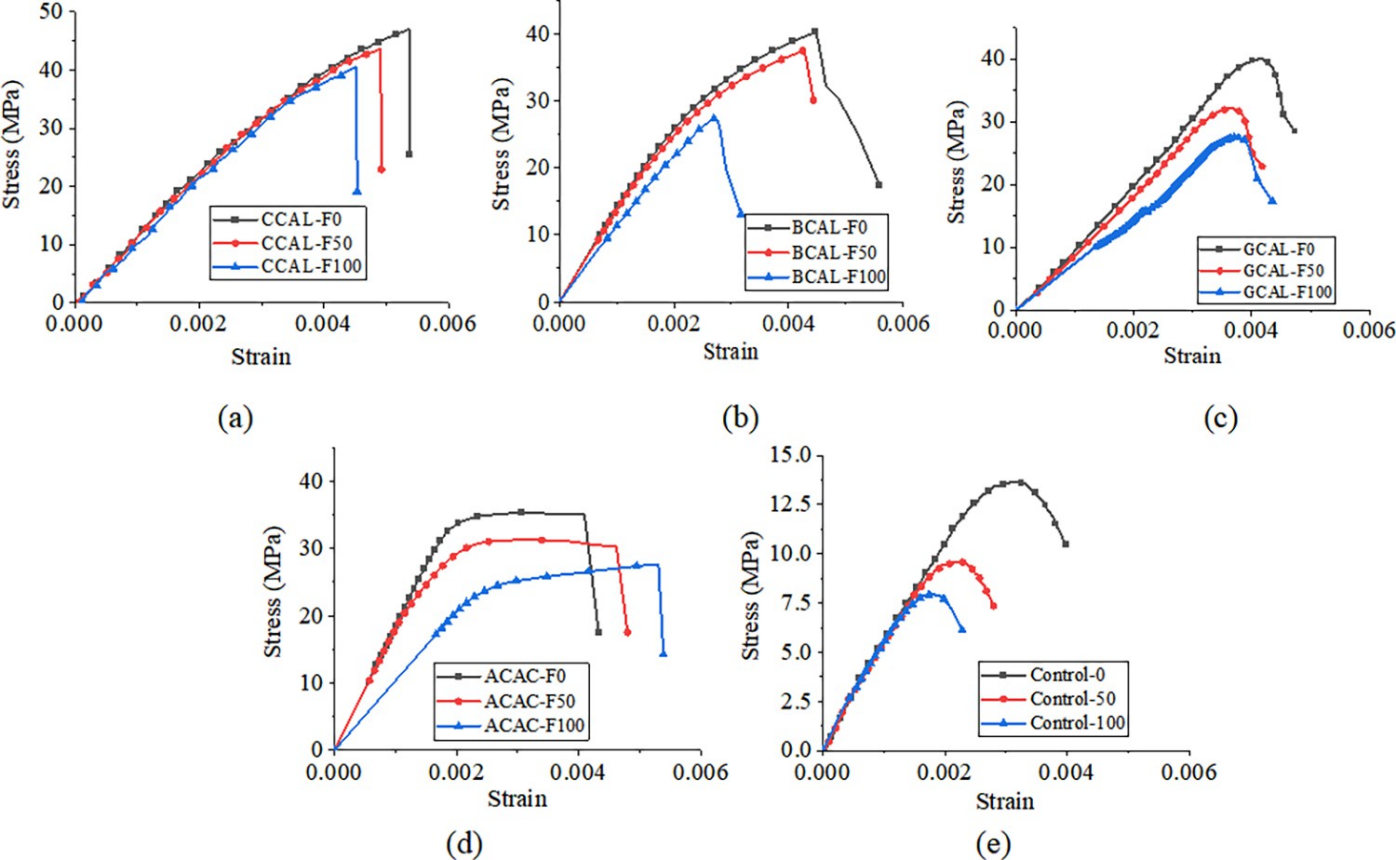
Uniaxial compressive stress-strain curve of cylindrical specimen under coupled alkali-freeze erosion: (a)CFRP reinforced specimen, (b) BFRP reinforced specimen, (c) GFRP reinforced specimen, (d) AFRP reinforced specimen, (e)Control group specimen.

In addition, the flexural properties of the CFRP-reinforced specimens at 0, 50, and 100 freeze-thaw cycles increased by 244.1%, 320.8%, and 450.1% compared with those of the unreinforced specimens, respectively, under the action of alkali-freeze coupling erosion, as shown in Fig 35(A). The flexural characteristics of BFRP-reinforced specimens at 0, 50, and 100 freeze-thaw cycles increased by 194.1%, 246.8%, and 290.6% compared with plain concrete specimens, respectively, as shown in Fig 35(B). The bearing capacity curves of AFRP and GFRP reinforced specimens show good plasticity. Meanwhile, the flexural characteristics of GFRP-reinforced specimens at 0, 50, and 100 freeze-thaw cycles increased by 195.6%, 236.4%, and 249.4%, respectively, compared with plain concrete specimens, as shown in Fig 35(C). The flexural characteristics of BFRP-reinforced specimens during 0, 50, and 100 freeze-thaw cycles increased by 160.3%, 226.0%, and 249.3%, respectively, compared with those of unreinforced specimens, as shown in Fig 35(D). Compared with the control group, the bearing capacity of each group is increased, which shows that FRP plays a good role in protecting concrete in the process of alkali-freeze-thaw cyclic coupling erosion. Compared with unreinforced specimens, each group’s ultimate bearing capacity is increased because FRP limits the transverse deformation of concrete and plays a strengthening role.

**Fig 36:**
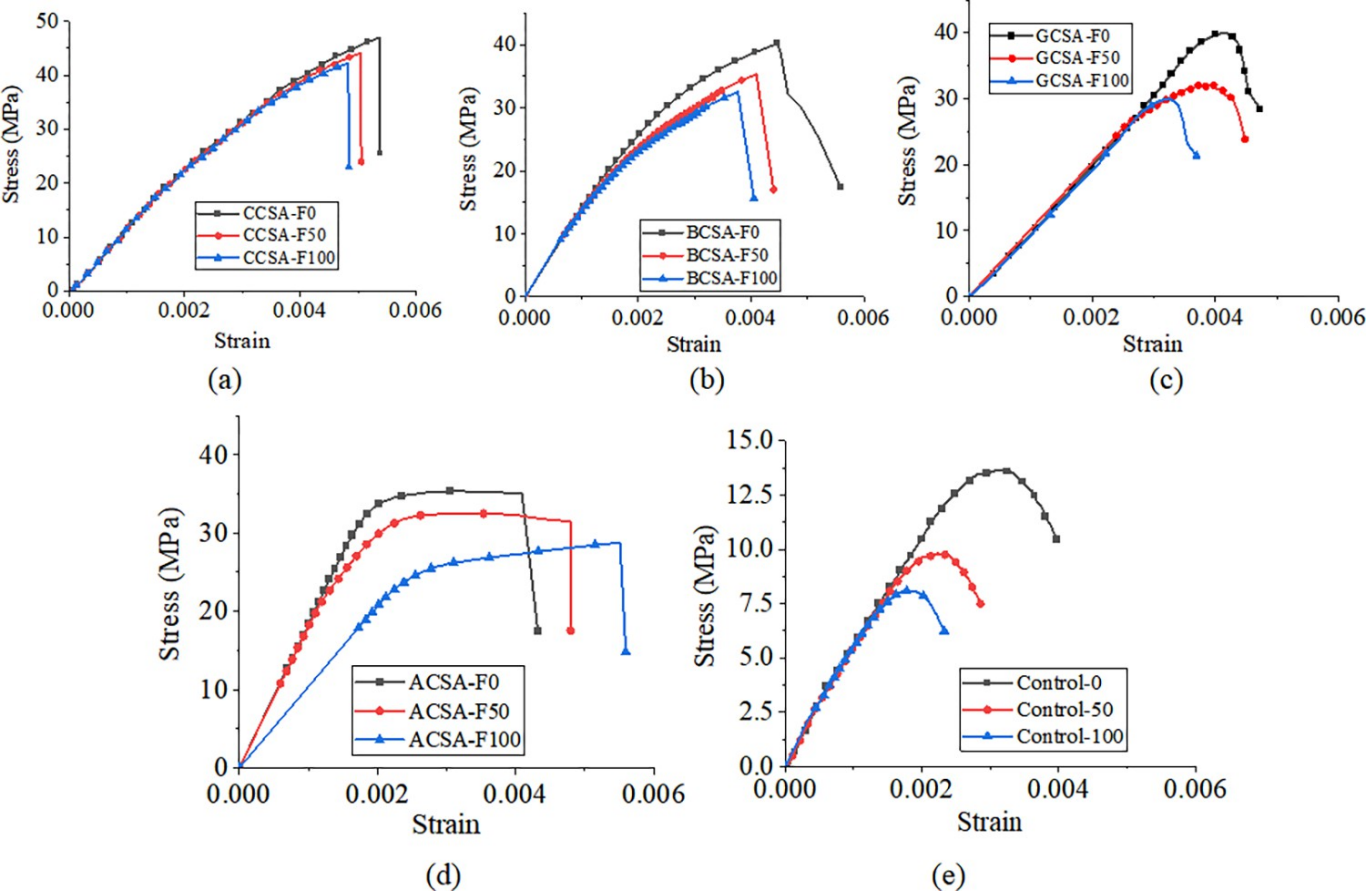
Uniaxial compressive stress-strain curve of cylindrical specimen under coupled salt-freeze erosion: (a)CFRP reinforced specimen, (b) BFRP reinforced specimen, (c) GFRP reinforced specimen, (d) AFRP reinforced specimen, (e)Control group specimen.

The variation of compressive strength of concrete cylindrical specimens reinforced with four kinds of fiber materials under the coupled salt-freeze-thaw cycle erosion environment is shown in Fig 36. It can be seen that the changes in compressive strength of concrete reinforced by four different fiber materials are similar. After 100 freeze-thaw cycles, the compressive strength of the concrete cylindrical specimens reinforced by CFRP, glass fiber cloth, basalt fiber cloth, and AFRP decreased by 8.3%, 19.2%, 27.2%, and 15.3%, respectively. The compressive strength of the specimens strengthened with carbon fiber has the slightest change. In addition, in chemical erosion environments, the unreinforced concrete prism specimen curve slowly rises and then suddenly drops. The curve of CFRP-reinforced concrete and BFRP-reinforced concrete specimens is similar to that of unreinforced concrete prismatic specimens. Also, it consists of a rising section and a rapidly falling section. The bearing capacity curves of AFRP and GFRP reinforced specimens show good plasticity.

### FRP reinforced prisms

**Fig 37:**
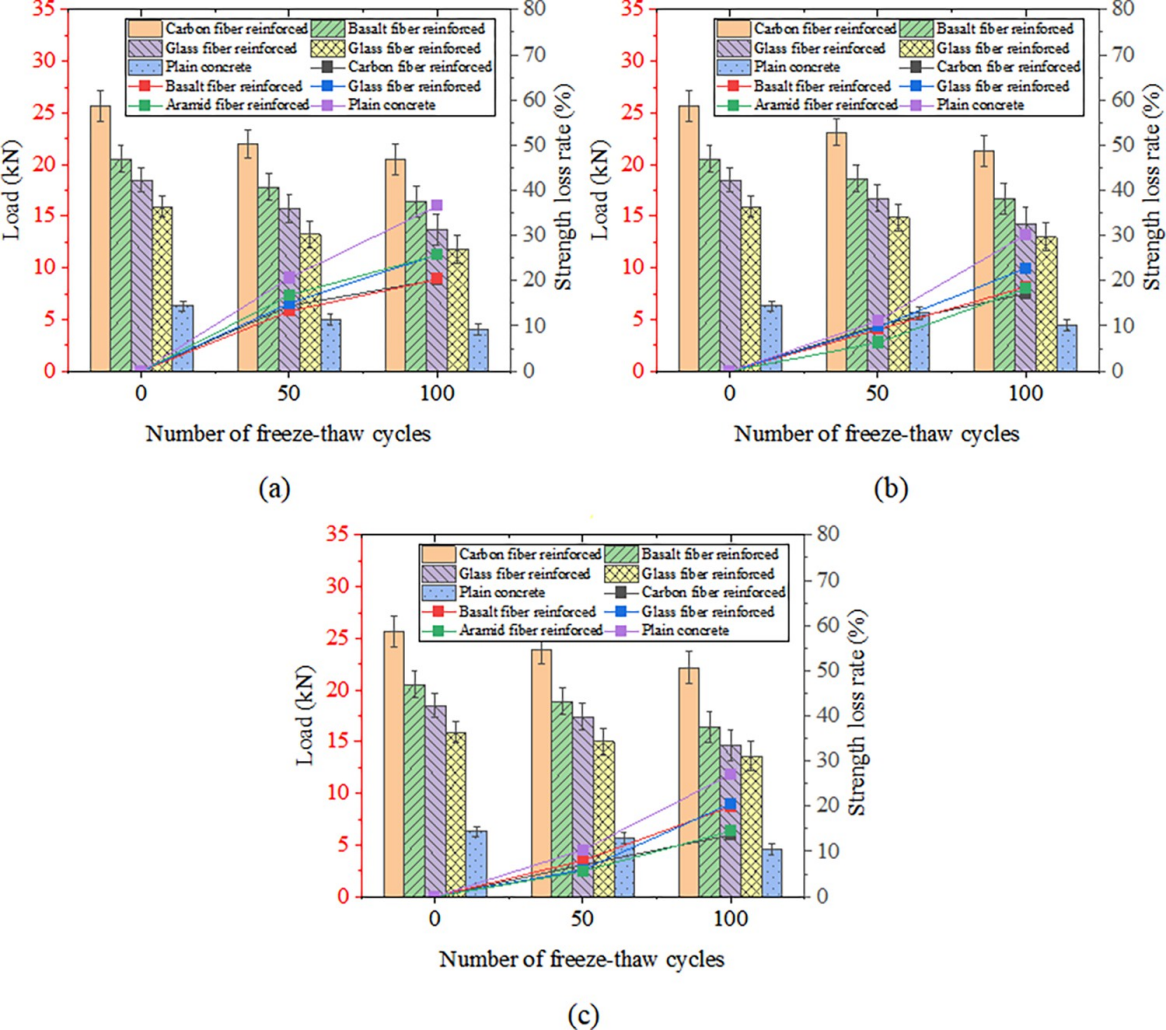
Flexural strength and loss rate: (a) coupled acid-freeze-thaw cyclic erosion; (b) coupled alkali-freeze-thaw cyclic erosion; (c) coupled salt-freeze-thaw cyclic erosion.

Fig 37(A) shows prismatic specimens’ flexural capacity, strength, and loss rate under coupled acid-freeze-thaw cyclic erosion. The bending capacity of the specimens in each group has a similar change rule, and the bending capacity of the specimens decreases with the increase of the acid-freeze-thaw coupling erosion cycle. Taking CFRP-reinforced specimens as an example, after 50 and 100 cycles of acid-freeze-thaw coupled erosion, the loss rates of bending capacity are 14.4% and 20.2%, respectively. The bending capacity of each group of specimens under different acid-freeze-thaw cycles also has the same rule: CFRP reinforced specimens>BFRP reinforced specimen>GFRP Reinforced specimen>AFRP reinforced specimen. In addition, the flexural capacity of prismatic specimens can be significantly improved by pasting FRP. Compared with the unreinforced specimens, the reinforced specimens of carbon fiber, basalt fiber, glass fiber, and aramid fiber increased by 412.5%, 308.8%, 243.0%, and 195.3%, respectively, after 100 times acid-freeze-thaw coupling erosion cycles. Relative to the initial bearing capacity, the bearing capacity loss rate of each group of specimens under 100 times of coupled erosion is in descending order of CFRP reinforced specimens (20.23%)<BFRP reinforced specimen (20.3%)<AFRP reinforced specimens (25.7%)<BFRP reinforced specimens (25.8%)<Unreinforced specimens (36.5%).

Fig 37(B) shows the column diagram of the flexural capacity of FRP-reinforced specimens and unreinforced specimens under alkali-freeze-thaw cyclic coupling erosion. It can be seen that the bending capacity of each group of specimens has the same rule under different alkali-freeze-thaw cycle coupling erosion cycles, and CFRP reinforced specimens>BFRP reinforced specimen>GFRP Reinforced specimen>AFRP Reinforced specimen>Unreinforced specimen. It is obvious that pasted fiber cloth greatly improves the bending capacity of concrete. Compared with the control group, CPAL-F100, BPAL-F100, GPAL-F100 and APAL-F100 increased by 384.1%, 279.5%, 225% and 195%, respectively. With the increase of the coupling erosion period, the bending capacity of all specimens decreased. Each group’s bearing capacity loss rate is CFRP<AFRP<BFRP<GFRP< Unreinforced specimen from small to large when the cyclic corrosion is 100 times.

The flexural strength and strength loss rate of prismatic specimens under coupled salt-freeze-thaw cyclic erosion are shown in Fig 37(C). The flexural strength of the reinforced prismatic specimen is higher than that of the unreinforced prismatic specimen, indicating that FRP can effectively improve the flexural strength of the specimen. After coupled salt-freeze-thaw cyclic erosion, the bending strength of unreinforced specimens and FRP-reinforced specimens decreased, and these changes became more evident with the increase of the coupled erosion period. The load-bearing capacity loss rate of specimens bonded with CFRP (acid freeze 20.23%, alkali freeze 17.12%, salt freeze 13.6%) is relatively the lowest compared to the initial state, while the load-bearing capacity loss rate of unreinforced specimens is the highest (acid freeze 36.5%, alkali freeze 30.16%, salt freeze 26.98%). After reaching the maximum tensile strength, their strength rapidly decreases, demonstrating a distinct brittleness. The stress-strain curve of FRP sheets differs slightly from that of epoxy resin adhesive, which exhibits a nonlinear behavior. The same research conclusion has also been reached in the literature [26].

In summary, with the increase of the coupled environmental period of the chemical-freeze-thaw cycle, the bond between concrete and FRP deteriorates, and the bearing capacity of the flexural specimens decreases. The bending capacity of CFRP reinforced specimens>BFRP reinforced specimen>GFRP Reinforced specimen>AFRP Reinforced specimen>Unreinforced specimen from small to large. The bearing capacity loss rates of CFRP, AFRP, and BFRP are smaller, while the bearing capacity loss rates of GFRP and unreinforced specimens are significant. CFRP, AFRP, and BFRP improve the durability of concrete wells.

### Constitutive model of concrete reinforced by alkali-freeze-thaw cyclic coupled FRP based on Lam-Teng

Mechanical properties are the most critical macroscopic properties of concrete materials, and the study of constitutive relations is a deep understanding of the mechanical properties of concrete. It is necessary to master the constitutive relation of materials for numerical simulation. According to the deformation characteristics of FRP-confined concrete, the Lam-Teng model holds that the deformation curve is composed of a parabolic section and a linear section, and the two sections are smoothly connected. Based on the Lam-Teng model, this paper studies the constitutive relationship of concrete reinforced by FRP under alkali-freeze-thaw cyclic coupling. The Lam-Teng model is as follows:

$$\sigma_{c}=E_{c}\varepsilon_{c}-\frac{{\left(E_{c}-E_{2}\right)}^{2}}{4f_{0}}\varepsilon_{c}^{2},0\leq \varepsilon_{c}\leq \varepsilon_{t}$$ (1)

$$\sigma_{c}=f_{0}+\varepsilon_{c}E_{2},\varepsilon_{t}\leq \varepsilon_{c}\leq \varepsilon_{cc}$$ (2)

Where, *σ*_*c*_ and *ε*_*c*_ are the compressive stress and compressive strain of FRP confined concrete, respectively; *E*_*c*_ is the elastic modulus of unconstrained concrete; *E*_*2*_ is the slope of the enhanced line segment; *f*_0_ is the intercept of the reverse extension line on the stress axis; *ε*_*t*_ is the strain at the connection of the parabola and the line, which is obtained from the condition that the slope of the parabola and the line is equal. From the condition that the slope at the connection of the parabola and the line is equal:

$$\varepsilon_{t}=\frac{2f_{0}}{E_{c}-E_{2}}$$ (3)

Enhanced line segment slope:

$$E_{2}=\frac{f_{cc}-f_{0}}{\varepsilon_{cc}}$$ (4)

**Fig 38:**
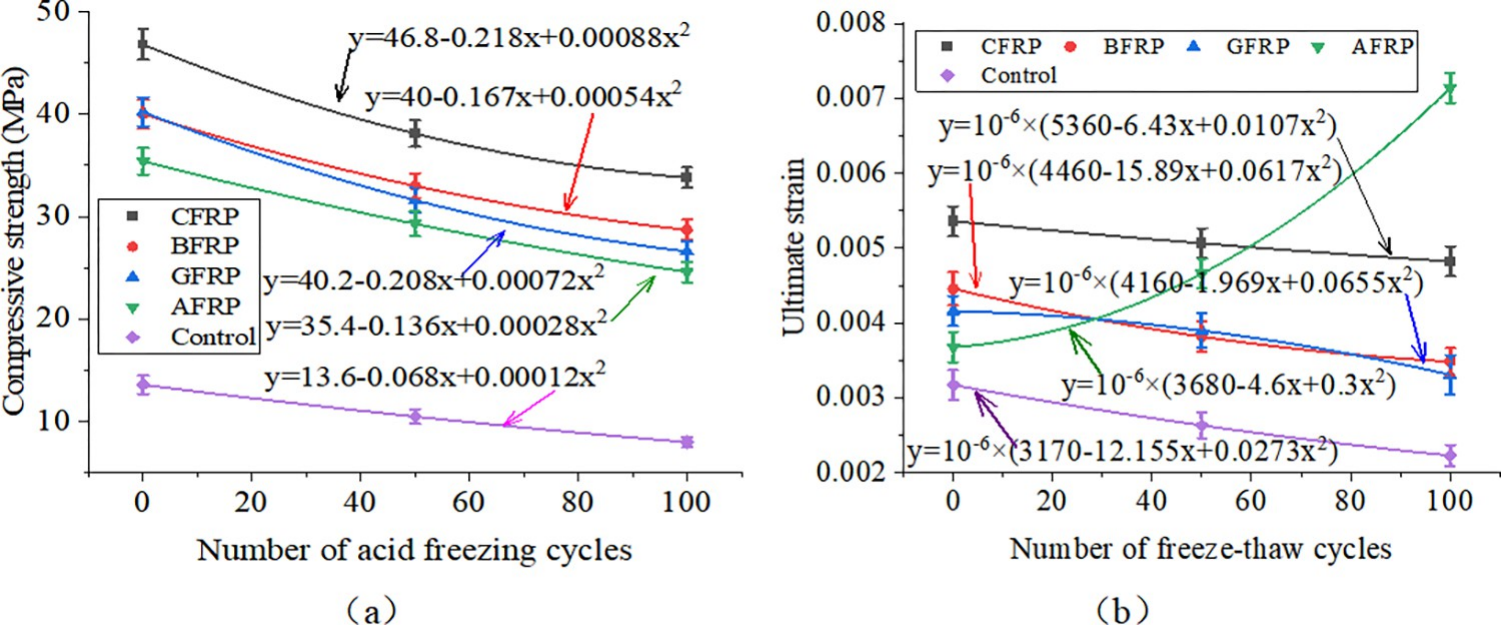
The fitting formula of compressive strength and ultimate strain of acid-freeze-thaw cyclic coupled erosion group:(a) compressive strength, (b) ultimate strain.

The relationship between chemical-freeze-thaw coupling erosion on compressive strength and the ultimate strain of FRP-reinforced concrete is explored. According to the test data, the formula of compressive strength and ultimate strain of FRP reinforced concrete induced by acid-freeze-thaw cyclic coupling erosion was fitted, and the relationship between compressive strength, ultimate strain, and coupled erosion period of different FRP reinforced concrete was obtained, as shown in Fig 38.

**Fig 39:**
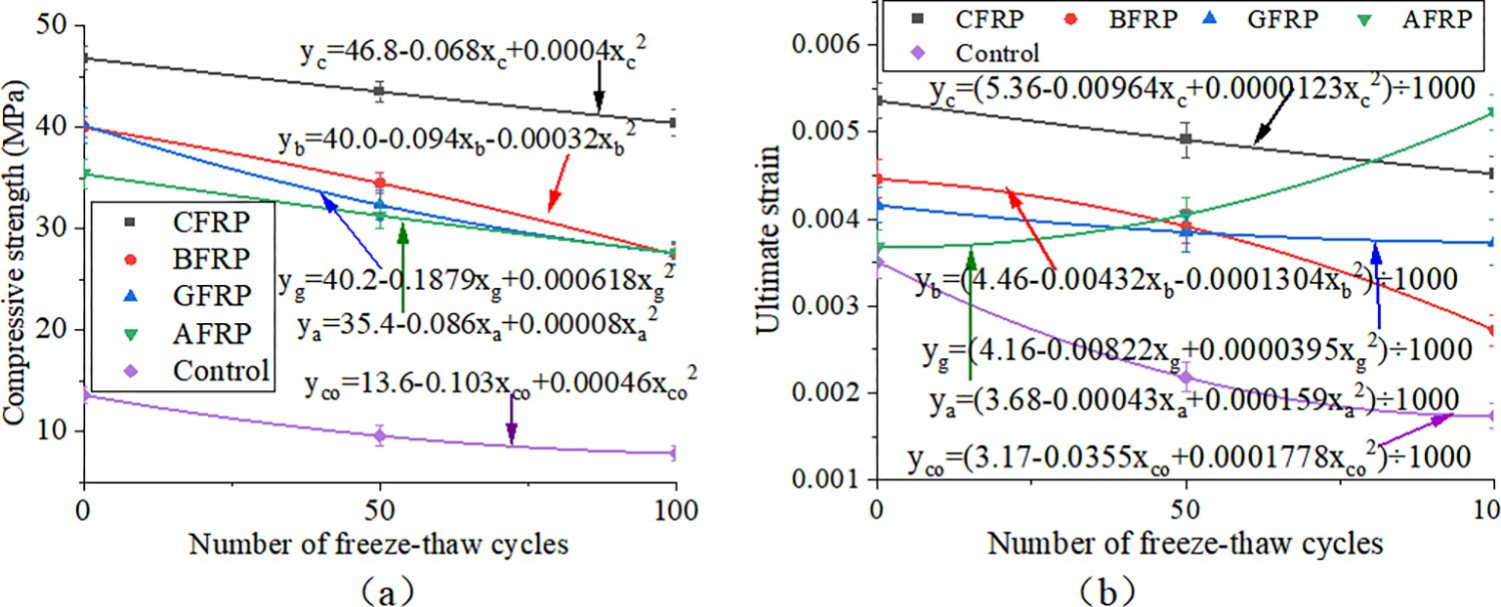
The fitting formula of compressive strength and ultimate strain of alkali-freeze-thaw cyclic coupled erosion specimen:(a) compressive strength, (b) ultimate strain.

**Fig 40:**
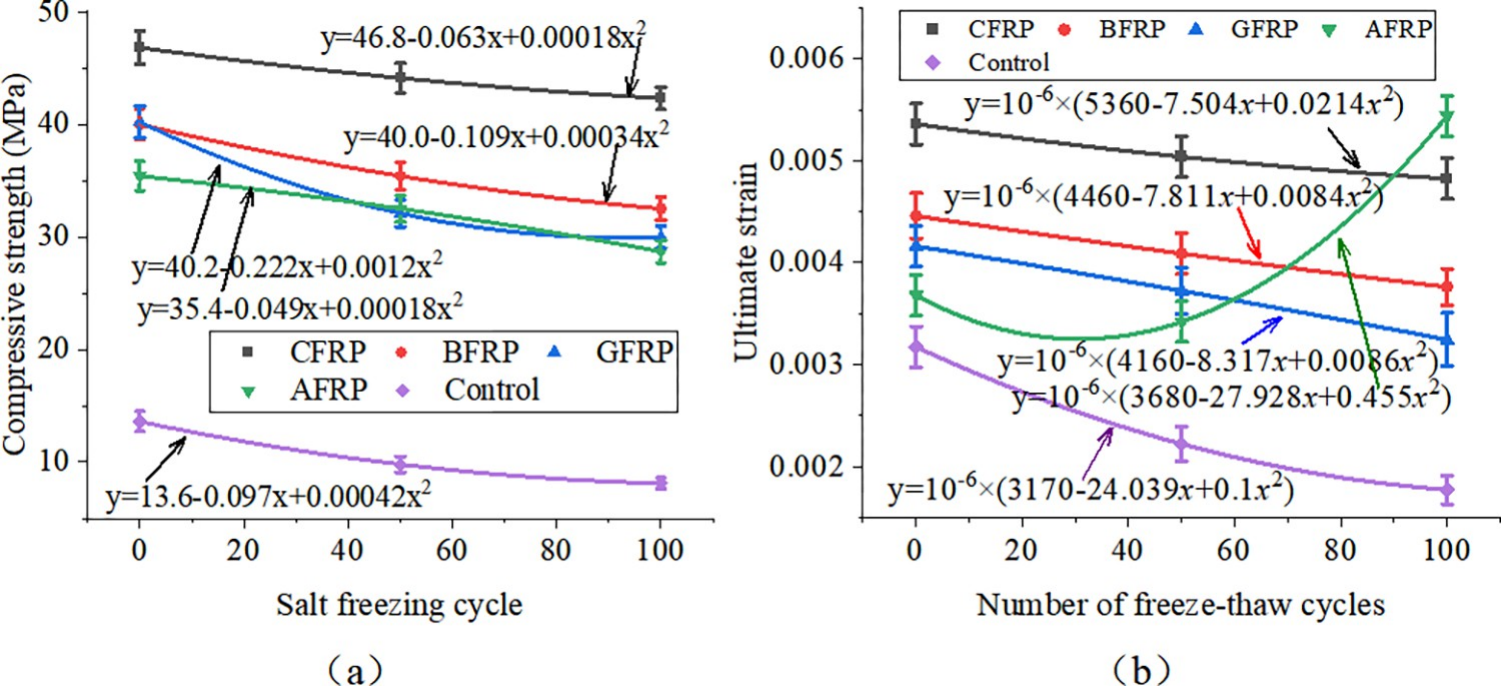
The fitting formula of compressive strength and ultimate strain of salt-freeze-thaw cyclic coupled erosion specimen.

Similarly, Figs 39 and 40 show the calculation results of alkali-freeze-thaw cyclic coupled erosion and salt-freeze-thaw cyclic coupled erosion 40, respectively.

According to the stress-strain curve of FRP-reinforced concrete shown in Fig 34, the characteristic parameters of the stress-strain curve without alkali-freeze-thaw cyclic coupling and acid-freeze-thaw cyclic coupling erosion were extracted. Table 4 lists some of the stress-strain curve parameters.

**Table 4:** Stress-strain curve parameters.

	*E*_*c*_	*f*_0_	εt	*f*_cc_ (MPa)	ε_cc_
CFRP reinforced specimen without corrosion	13000	9.9	0.0032	46.8	0.00536
GFRP reinforced specimen without corrosion	14800	15.0	0.0024	40.2	0.00416
BFRP reinforced specimen without corrosion	11500	5.0	0.0028	40.0	0.00446
AFRP reinforced specimen without corrosion	18200	32.2	0.0021	35.4	0.00351

**Fig 41:**
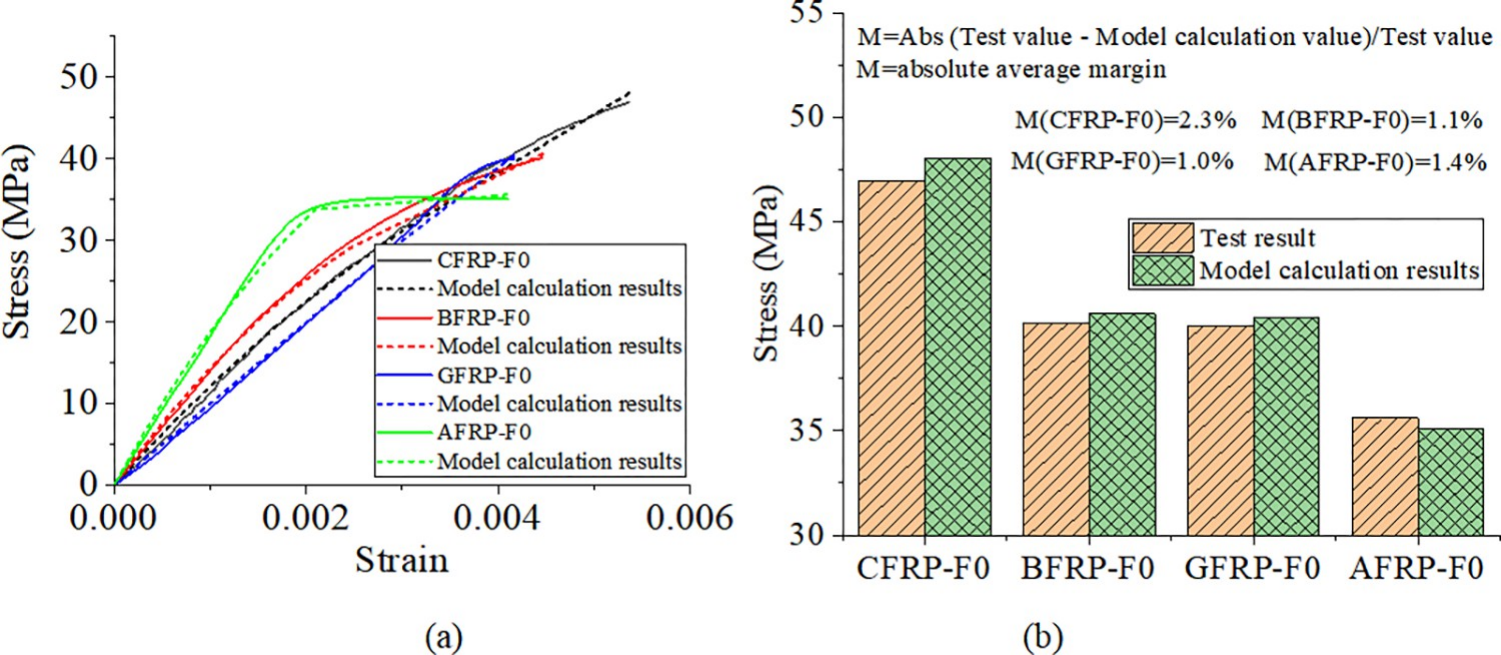
Comparison of calculated and experimental, (a) stress-strain curves, (b)maximum stress.

Put the parameters in Table 4 into Formula 1–Formula 4 and compare the experimental and calculation curves, as shown in Fig 41(A). The experimental results agree with the calculated results. Fig 41(B) compares the maximum stress values obtained from experiments and model calculated.An absolute average margin (M) is defined to quantify the error. It can be observed that the maximum M value for CFRP is 2.3%, while the maximum M value for GFRP is 1.0%. This indicates that there is a relatively small error between the maximum stress values obtained from the model calculations and the experimental results. So, the Lam-Teng model can simulate the constitutive relationship of FRP-reinforced concrete well.

The Lam-Teng model was modified to study the constitutive model of concrete reinforced by FRP under the coupling of the alkali-freeze-thaw cycle. Based on the relationship between the compressive strength and ultimate strain of FRP-reinforced concrete and the number of freeze-thaw cycles and combined with the above formula, the Lam-Teng model equation is modified for the stress-strain curve of FRP-reinforced concrete under the coupling of different alkali-freeze-thaw cycles, as shown in Table 5.

**Table 5:** Modified Lam-Teng model equation.

Number	Proposed stress-strain curve
CFRP reinforced specimen	$\sigma_{c}=E_{c}\varepsilon_{c}-\frac{{\left(E_{c}\varepsilon_{cc}-f_{C}+f_{0}\right)}^{2}}{4f_{0}}{\varepsilon_{c}}^{2}$, 0≤*ε*_*c*_<*ε*_*t*_; $\sigma_{c}=f_{0}+\varepsilon_{c}\frac{f_{C}-f_{0}}{\varepsilon_{cc1}}$, *ε*_*t*_≤*ε*_*c*_≤*ε*_*cc*1_
BFRP reinforced specimen	$\sigma_{c}=E_{c}\varepsilon_{c}-\frac{{\left(E_{c}\varepsilon_{cc}-f_{B}+f_{0}\right)}^{2}}{4f_{0}}{\varepsilon_{c}}^{2}$, 0≤*ε*_*c*_<*ε*_*t*_; $\sigma_{c}=f_{0}+\varepsilon_{c}\frac{f_{B}-f_{0}}{\varepsilon_{cc2}}$, *ε*_*t*_≤*ε*_*c*_≤*ε*_*cc*2_
GFRP reinforced specimen	$\sigma_{c}=E_{c}\varepsilon_{c}-\frac{{\left(E_{c}\varepsilon_{cc}-f_{G}+f_{0}\right)}^{2}}{4f_{0}}{\varepsilon_{c}}^{2}$, 0≤*ε*_*c*_<*ε*_*t*_; $\sigma_{c}=f_{0}+\varepsilon_{c}\frac{f_{G}-f_{0}}{\varepsilon_{cc3}}$, *ε*_*t*_≤*ε*_*c*_≤*ε*_*cc*3_
AFRP reinforced specimen	$\sigma_{c}=E_{c}\varepsilon_{c}-\frac{{\left(E_{c}\varepsilon_{cc}-f_{A}+f_{0}\right)}^{2}}{4f_{0}}{\varepsilon_{c}}^{2}$, 0≤*ε*_*c*_<*ε*_*t*_; $\sigma_{c}=f_{0}+\varepsilon_{c}\frac{f_{A}-f_{0}}{\varepsilon_{cc4}}$, *ε*_*t*_≤*ε*_*c*_≤*ε*_*cc*4_

## Conclusion

The mechanical and apparent deterioration laws of carbon/basalt/glass/aramid fiber cloth reinforced concrete under chemistry-freeze-thaw coupling were studied. The tensile strength of the FRP sheet and epoxy resin sheet before and after chemistry freeze, the compressive strength of the fully wrapped FRP cylindrical test block, and the bending capacity of the prismatic test block pasted with FRP on the precast crack side were tested. Based on the Lam-Teng model, the constitutive model of concrete reinforced by chemically frozen FRP was modified. The conclusion is as follows:

1. In terms of epigenetic observation, with the chemical-freeze erosion cycle increases, the surface holes of epoxy resin sheets increase, and there are tiny cracks and folds. The texture of FRP sheets is blurred, and there are cracks and folds on the surface. FRP reinforcement can effectively improve the durability of concrete in the coupled environment of the acid-freeze-thaw cycle, and carbon fiber and aramid fiber have the best protection effect on concrete, followed by basalt fiber and glass fiber.
2. In terms of the failure state of the specimen, as the chemical coupling erosion cycle increases, the failure position of the epoxy resin sheet becomes uncertain, and the failure direction tilts towards the load direction. The failure mode of FRP sheets has not changed. For compressive specimens, the failure of AFRP reinforced specimens showed strong ductility in three chemical erosion environments, while other FRP reinforced specimens exhibited brittle failure. For flexural specimens, after chemical freeze-thaw cycle coupling, the bonding strength between FRP and concrete decreases, the reinforcement effect weakens, and FRP peels off from the surface of concrete.
3. In terms of mechanical properties, the mechanical degradation of FRP sheets is most severe in acid freezing environments, and the tensile properties from high to low are CFRP, BFRP, AFRP, and GFRP. The curves drawn from the tensile test data indicate that the early stage is linear elastic, and the strain suddenly decreases when it reaches the maximum stress.
4. In terms of mechanical properties, for cylindrical specimens, the compressive strength loss rates of specimens bonded with CFRP (acid freeze minimum loss rate (27.8%), salt freeze minimum loss rate (9.61%)), BFRP (alkali freeze minimum loss rate (11.5%), and AFRP are relatively small compared to the initial state, while GFRP has a larger strength loss rate (acid freeze (33.8%), salt freeze (25.37%)). The stress-strain curve of the unreinforced specimen slowly rises and suddenly decreases after reaching the maximum stress. The curve changes of CFRP reinforced and BFRP reinforced concrete specimens are similar to those of unreinforced specimens. The curves of AFRP reinforced and GFRP reinforced specimens showed good plasticity. For prismatic specimens, the load-bearing capacity loss rate of specimens bonded with CFRP (acid frost 20.23%, alkali frost 17.12%, salt frost 13.6%) is relatively the lowest compared to the initial state, while the load-bearing capacity loss rate of unreinforced specimens is the highest (acid frost 36.5%, alkali frost 30.16%, salt frost 26.98%).
5. Calculate the Lam Teng stress-strain curve of the unreinforced specimen, and the experimental curve matches well with the model calculation curve. For the absolute average margin of maximum stress calculated from experiments and models, CFRP reinforced specimens have a maximum value of 2.3%, while GFRP reinforced specimens have a minimum value of 1.0%, which is verified by the experimental curve. Analyzed the relationship between peak stress-strain and coupled freeze-thaw cycles, and established a Lam Teng constitutive model for chemical freeze coupling FRP reinforced concrete.

## Supporting information

S1 Raw images (PDF)
