# Supplementary material for: Investigation on damage evolution mechanism of various FRP strengthened concrete subjected to chemical-freeze-thaw coupling erosion
Source: PLoS One. 2024 May 21;19(5):e0303645. doi: 10.1371/journal.pone.0303645 (PMC11108217; doi:10.1371/journal.pone.0303645)
Supplement: S1 Raw images — (PDF) [file pone.0303645.s001.pdf]

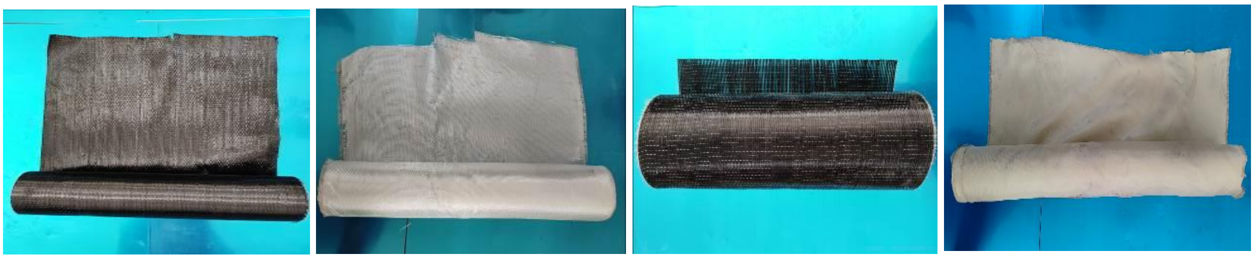

（a） （b） （c） （d）

Fig 1. Four kinds of fiber composites:(a) CFRP, (b) GFRP, (c) BFRP, (d) AFRP

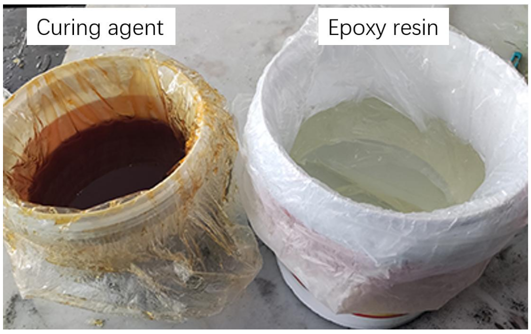

Fig 2. epoxy resin glue

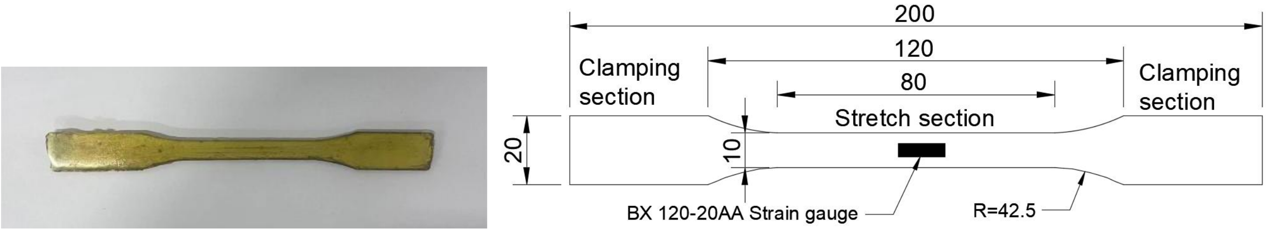

（a） （b）

Fig 3. Epoxy resin: (a) Epoxy resin specimen, (b)Specimen size diagram (unit: mm)

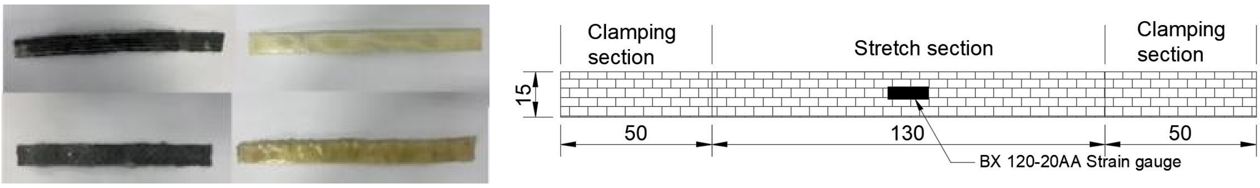

（a） （b）

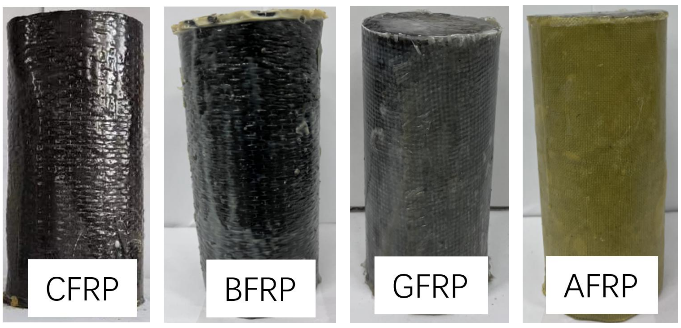

（a）　　（b）　　（c）　　（d）

Fig. 5 Fiber cloth reinforced concrete compression specimen: (a) CFRP, (b) BFRP, (c) GFRP, (d) AFRP

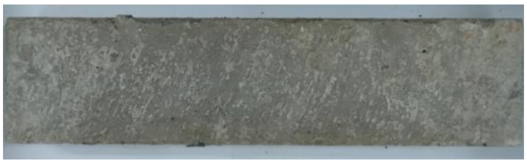

（a）

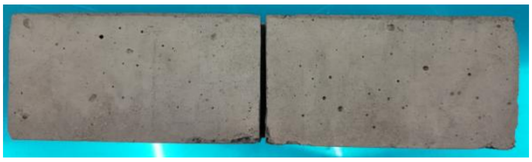

（b）

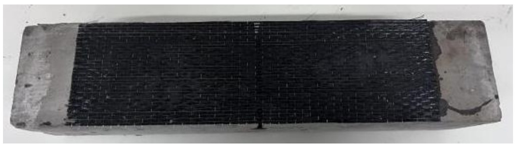

(c)

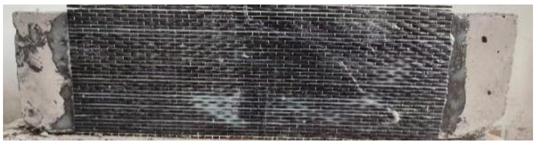

(d)

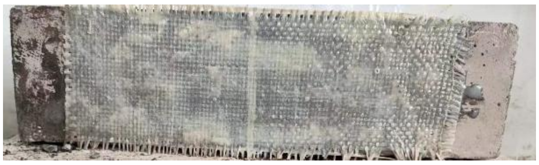

（e）

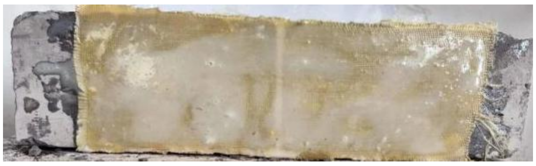

（f）

Fig 6. FRP reinforced bending specimen:(a) Unreinforced bending specimen, (b) Slotted bending specimen, (c) CFRP reinforced bending specimen, (d) BFRP reinforced bending specimen, (e) GFRP reinforced bending specimen, (f) AFRP reinforced bending specimen

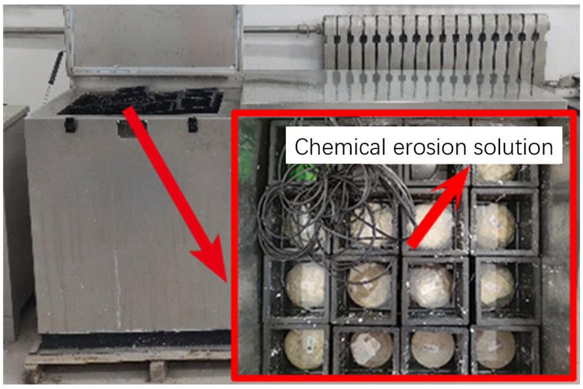

Fig 7. Chemistry-freeze-thaw cycle coupled immersion environment

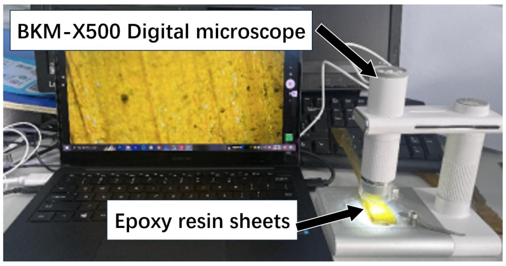

（a）

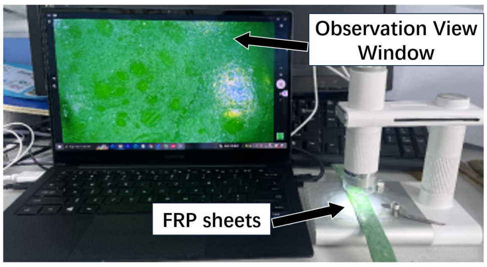

（b）

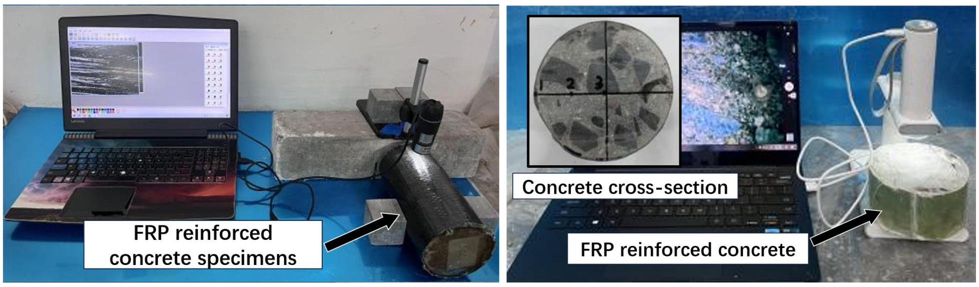

（c）　　　　　　　　　　（d）

Fig 8. Specimen microscopic observation: (a) Epoxy resin, (b) FRP sheet, (c) FRP reinforced concrete, (d) FRP reinforced concrete cross section resin

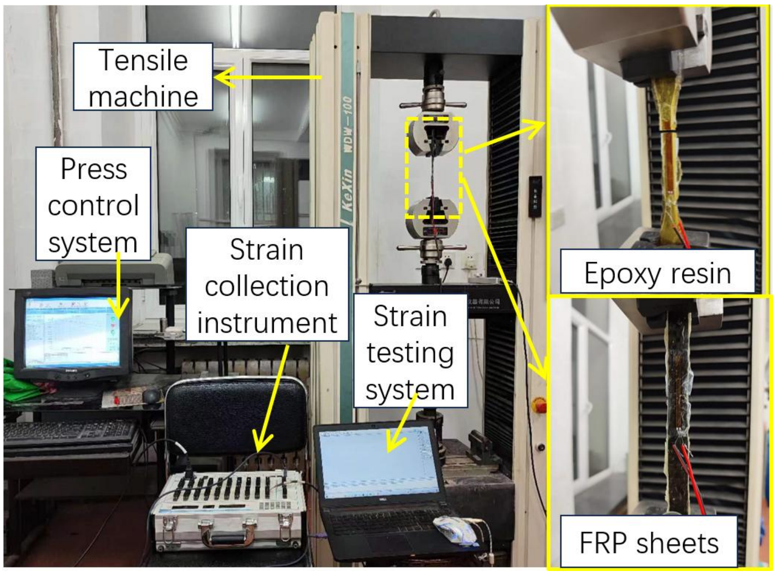

Fig 9. Tensile mechanical test

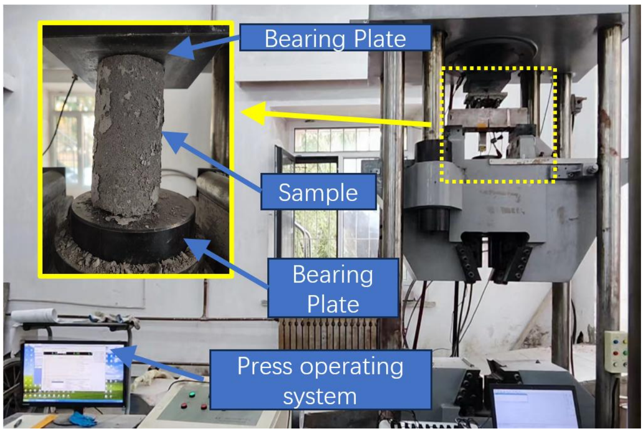

Fig 10. Mechanical test device

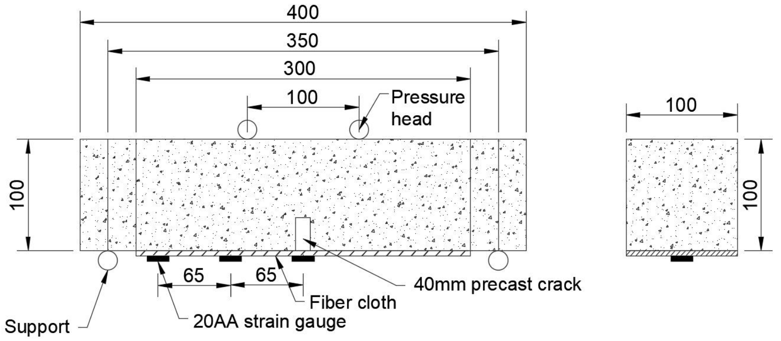

Fig 11. Bending specimen size and strain gauge layout (unit: mm)

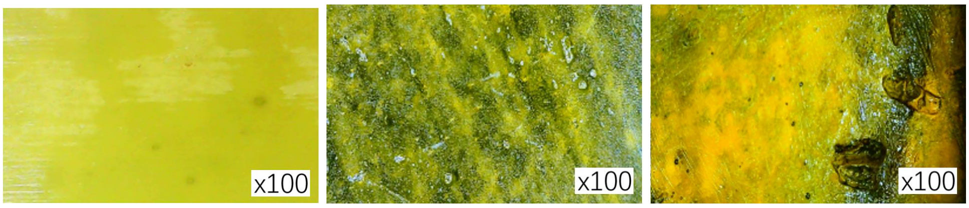

EPN-F0 EPAC-F50 EPAC-F100

（a）

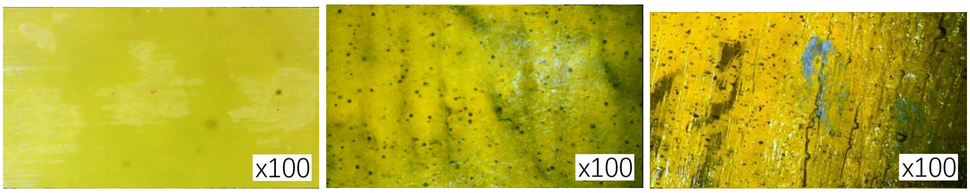

EPN-F0 EPAL-F50 EPAL-F100

（b）

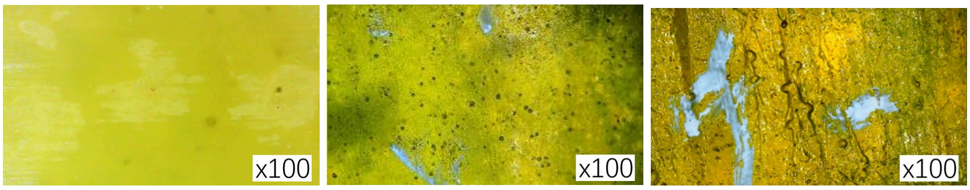

EPN-F0 EPSA-F50 EPSA-F100

（c）

Fig 12. Microscopic imaging of epoxy resin :(a) acid-freeze cycle erosion, (b) alkali-freeze cycle erosion, (c) salt-freeze cycle erosion

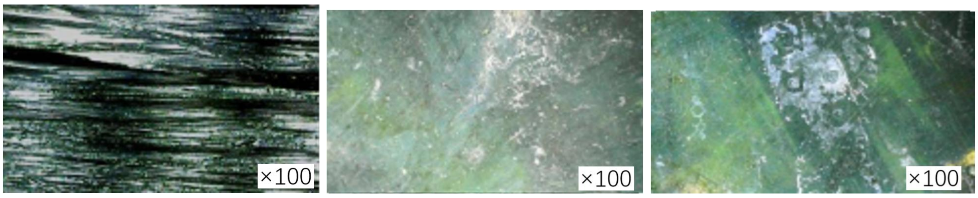

CS-F0 CSAC-F50 CSAC-F100

（a）

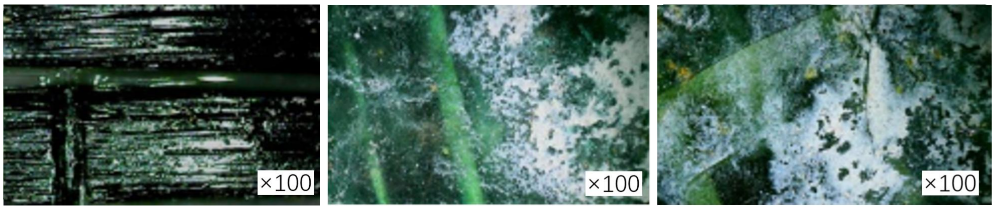

BSAC-F0 BSAC-F50 BSAC-F100

(b)

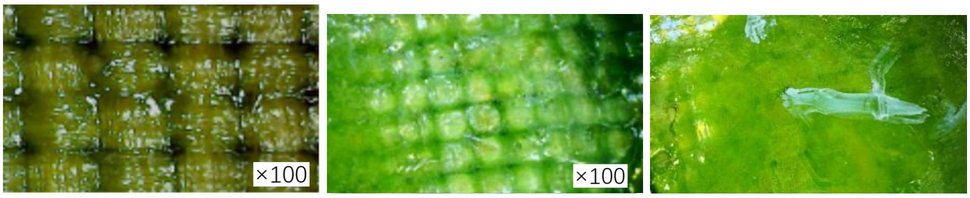

ASAC-F0 ASAC-F50 ASAC-F100

(c)

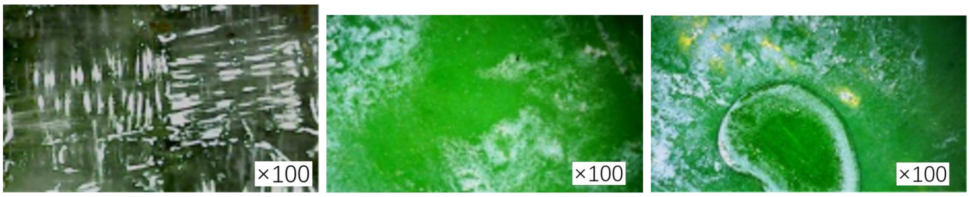

GSAC-F0 GSAC-F50 GSAC-F100

(d)

Fig 13. Microscopic imaging of acid-frost erosion FRP sheets: (a) CFRP, (b) BFRP, (c) GFRP, (d) AFRP

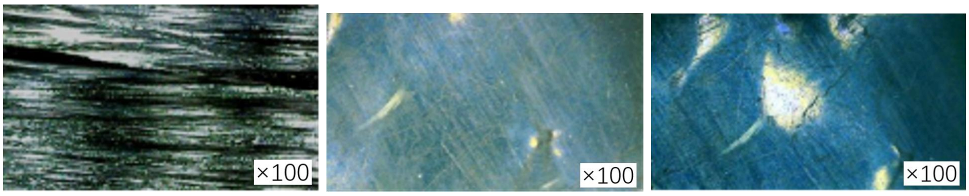

CSN-F0 CSAL-F50 CSAL-F100

(a)

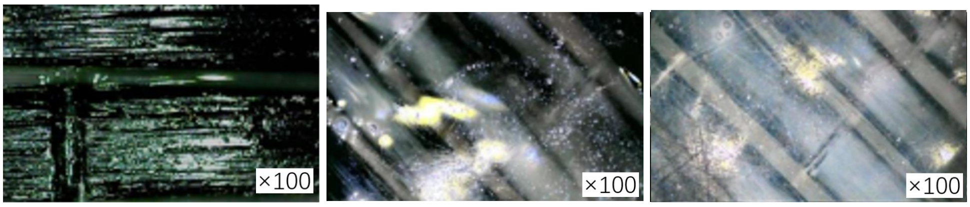

BSN-F0 BSAL-F50 BSAL-F100

(b)

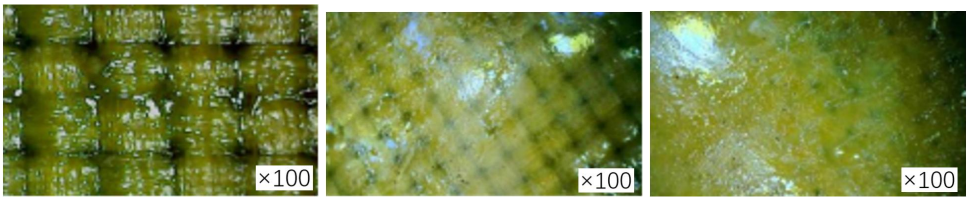

ASN-F0 ASAL-F50 ASAL-F100

(c)

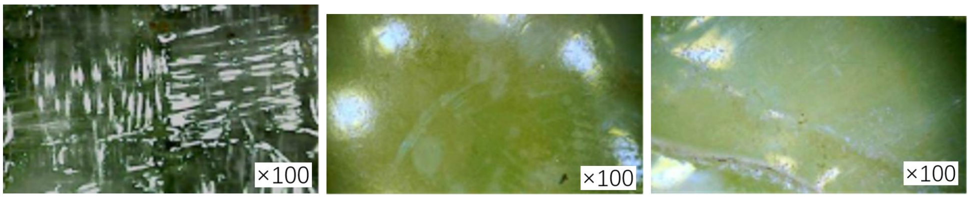

GSN-F0 GSAL-F50 GSAL-F100

(d)

Fig 14. Microscopic imaging of alkali-freeze erosion FRP sheets: (a) CFRP, (b) BFRP, (c) GFRP, (d) AFRP

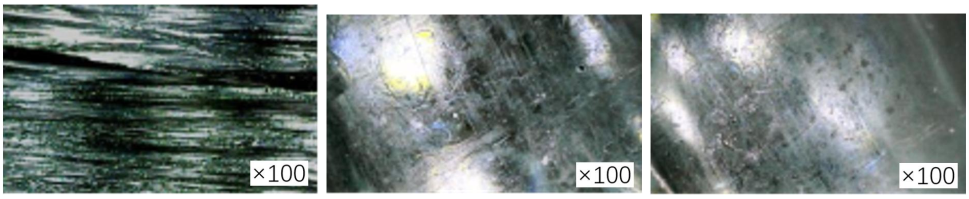

CSN-F0-100 CSS-F50-100 CSS-F100-100

（a）

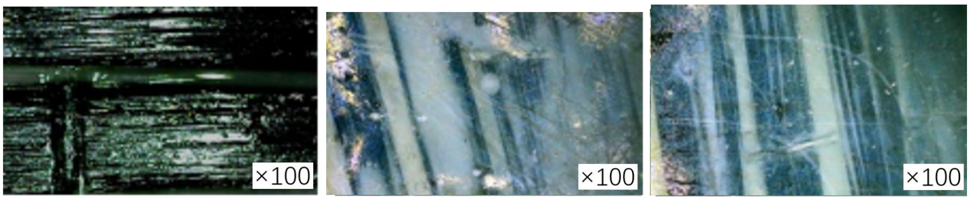

BSN-F0-100 BSS-F50-100 BSS-F100-100

（b）

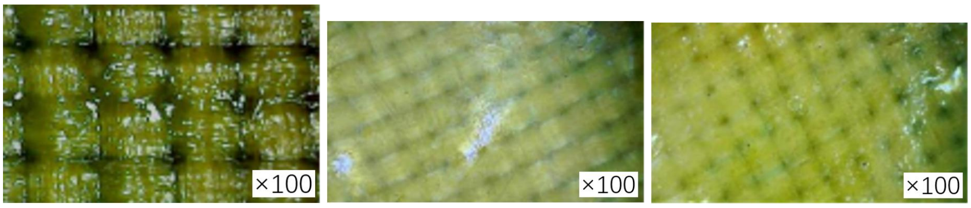

ASN-F0-100 ASS-F50-100 ASS-F100-100

（c）

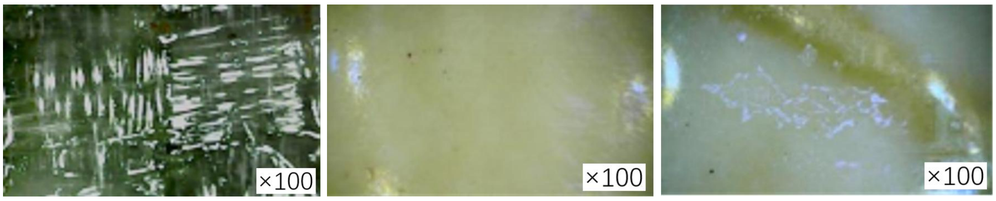

GSN-F0-100 GSS-F50-100 GSS-F100-100

（d）

Fig 15. Microscopic imaging of salt-freeze erosion FRP sheets: (a) CFRP, (b) BFRP, (c) GFRP, (d) AFRP

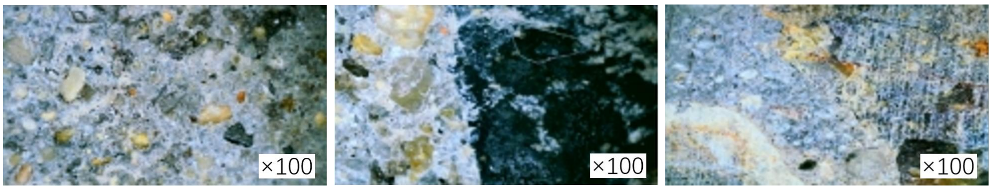

PAC1-F50 PAC2-F50 PAC3-F50

（a）

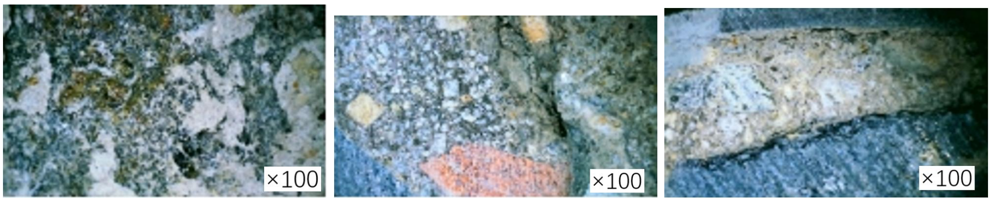

PAC1-F100 PAC2-F100 PAC3-F100

（b）

Fig 16. Microstructure of cross section of unreinforced specimen(a) Under 50 cycles of acid freeze-thaw erosion conditions, (b) Under 100 cycles of acid freeze-thaw erosion conditions

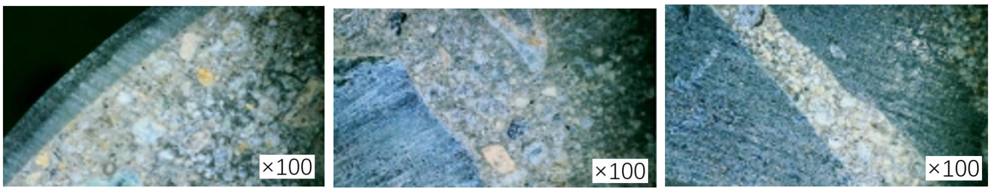

CAC1-F50 CAC2-F50 CAC3-F50

（a）

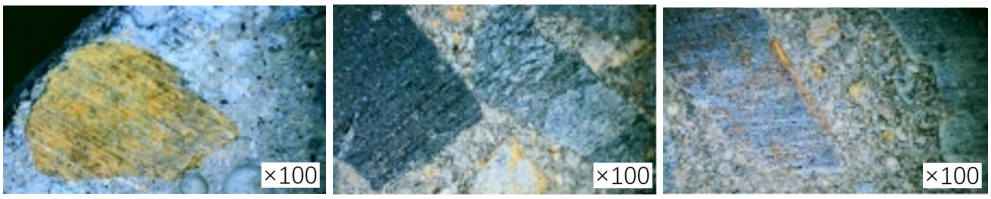

CAC1-F100 CAC2-F100 CAC3-F100

（b）

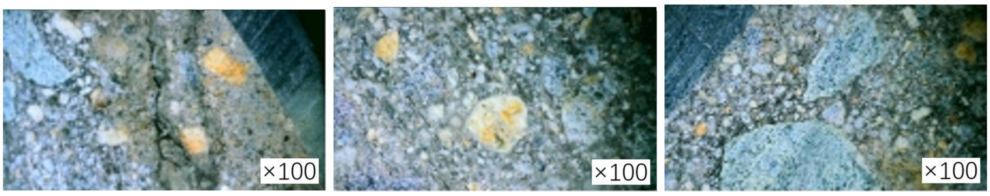

BAC1-F50 BAC2-F50 BAC3-5F0

（c）

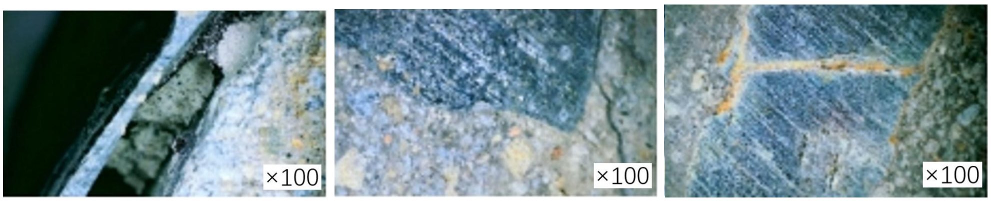

BAC1-F100 BAC2-F100 BAC3-F100

（d）

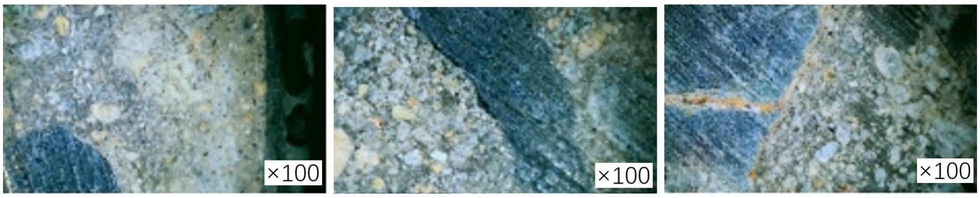

GAC1-F50 GAC2-F50 GAC3-F50

（e）

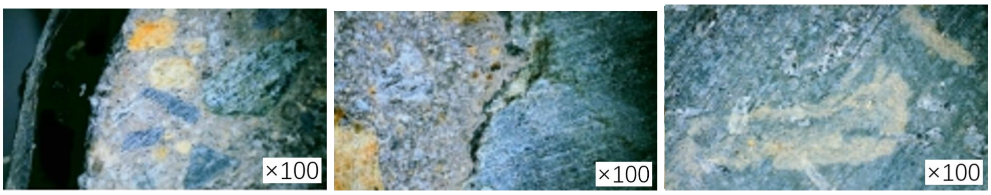

GAC1-F100 GAC2-F100 GAC3-F100

（f）

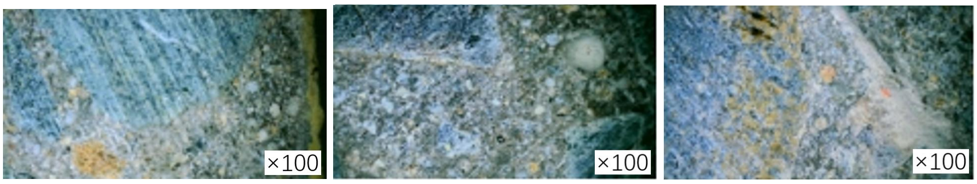

AAC1-F50 AAC2-F50 AAC3-F50

（g）

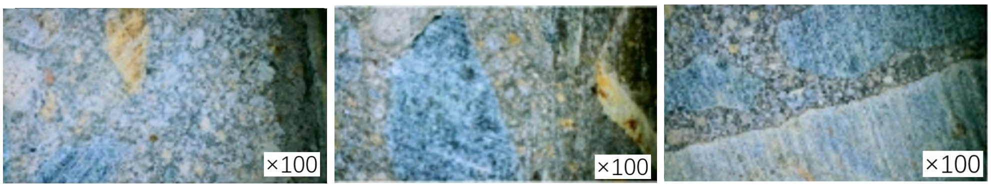

AAC1-F100 AAC2-F100 AAC3-F100

（h）

Fig 17. Microstructure of FRP reinforced specimens: (a) CFRP reinforced specimens subjected to 50 cycles of acid freeze cycles, (b) CFRP specimens subjected to 100 cycles of acid freeze cycles; (c) BFRP specimens subjected to 50 cycles of acid freeze cycles, (d) BFRP specimens subjected to 100 cycles of acid freeze cycles, (e) GFRP specimens subjected to 50 cycles of acid freeze cycles, (f) GFRP specimens subjected to 100 cycles of acid freeze cycles, (g) AFRP specimens subjected to 50 cycles of acid freeze cycles, (h) AFRP specimens subjected to 100 cycles of acid freeze cycles

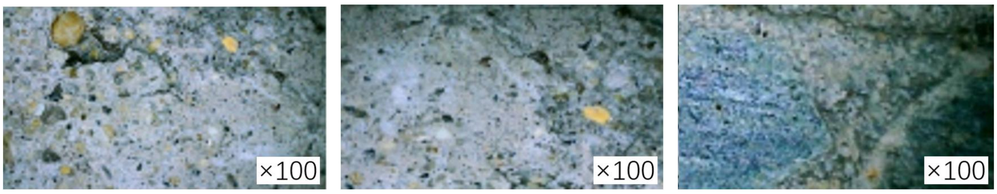

PAL1-F50 PAL2-F50 PAL3-F50

（a）

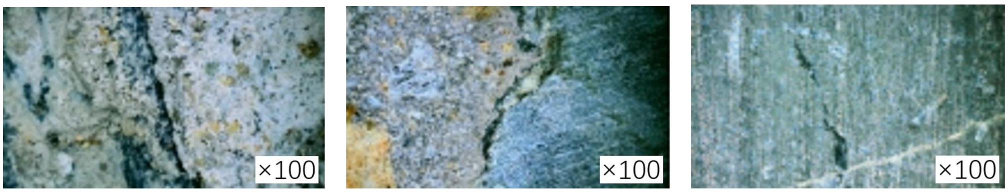

PAL1-F100 PAL2-F100 PAL3-F100

（b）

Fig 18. Microstructure of cross section of unreinforced specimen:(a) Under 50 cycles of alkaline freeze-thaw erosion conditions, (b) Under 100 cycles of alkaline freeze-thaw erosion conditions

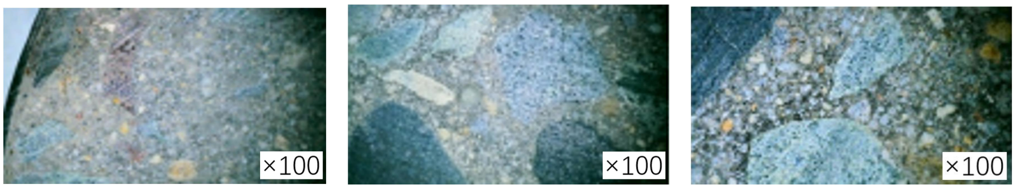

CCAL1-F50 CCAL2-F50 CCAL3-F50

（a）

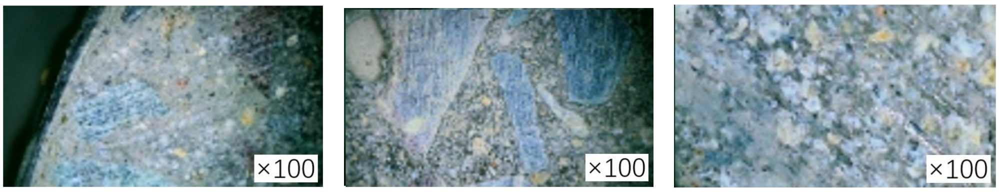

CCAL1-F100 CCAL2-F100 CCAL3-F100

（b）

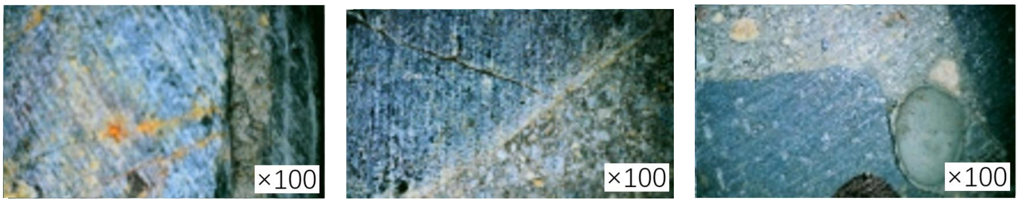

BCAL1-F50 BCAL2-F50 BCAL3-F50

（c）

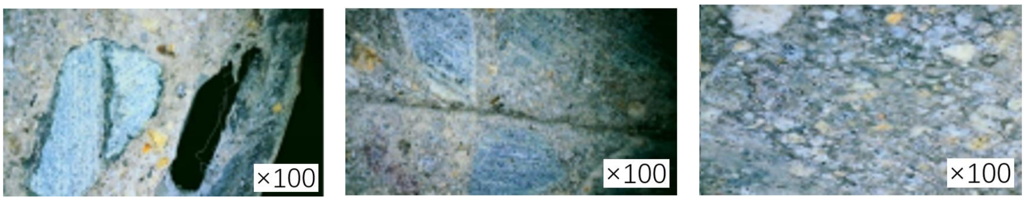

BCAL1-F100 BCAL2-F100 BCAL3-F100

（d）

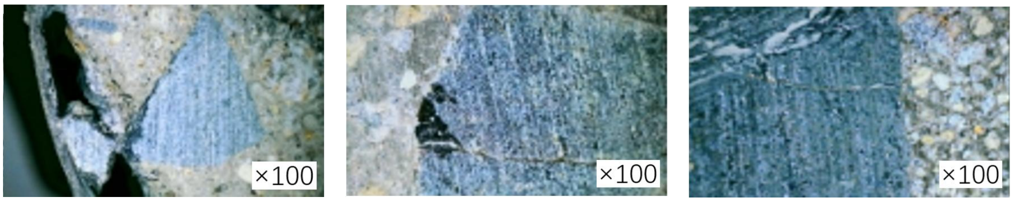

GCAL1-F50 GCAL2-F50 GCAL3-F50

（e）

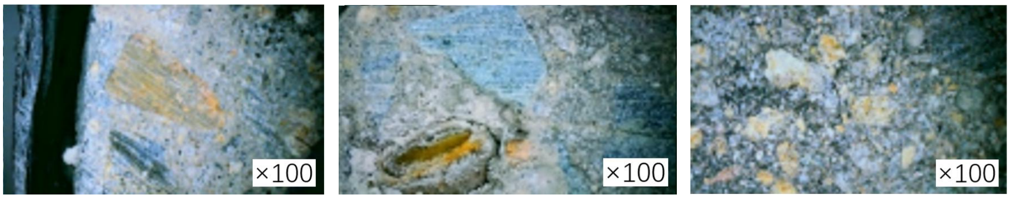

GCAL1-F100 GCAL2-F100 GCAL3-F100

（f）

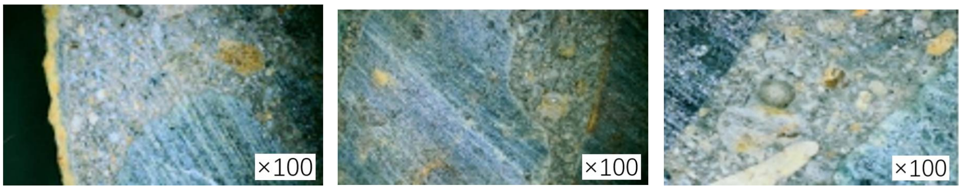

ACAL1-F50 ACAL2-F50 ACAL3-F50

（g）

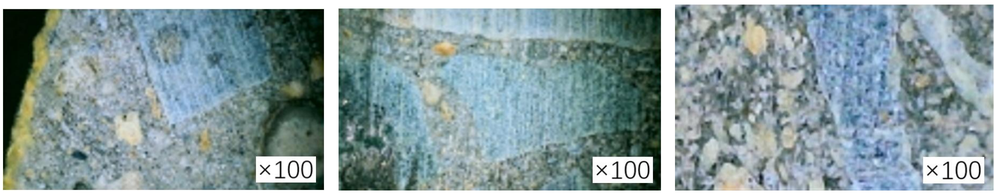

ACAL1-F100 ACAL2-F100 ACAL3-F100

（h）

Fig 19. Microstructure of the FRP reinforced specimen: (a) CFRP reinforced specimens subjected to 50 cycles of alkali freeze cycles, (b) CFRP specimens subjected to 100 cycles of alkali freeze cycles; (c) BFRP specimens subjected to 50 cycles of alkali freeze cycles, (d) BFRP specimens subjected to 100 cycles of alkali freeze cycles, (e) GFRP specimens subjected to 50 cycles of alkali freeze cycles, (f) GFRP specimens subjected to 100 cycles of alkali freeze cycles, (g) AFRP specimens subjected to 50 cycles of alkali freeze cycles, (h) AFRP specimens subjected to 100 cycles of alkali freeze cycles

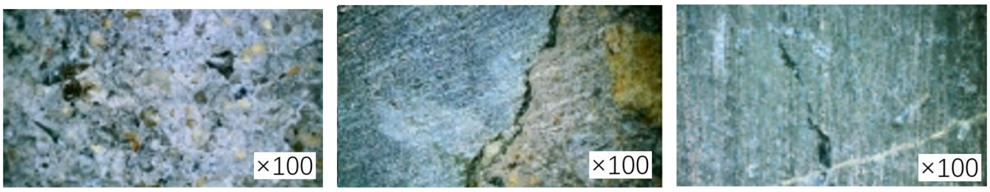

P1S-F100 P2S-F100 P3S-F100

Figure 20 Microstructure of cross section of unreinforced specimen

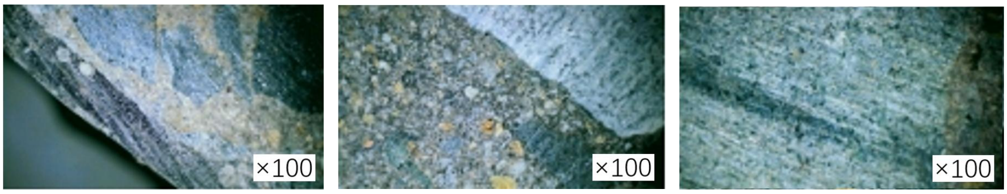

C1S-F50 C2S-F50 C3S-F50

（a）

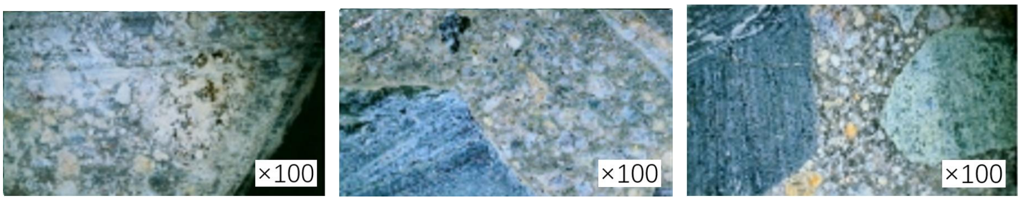

C1S-F100 C2S-F100 C3S-F100

（b）

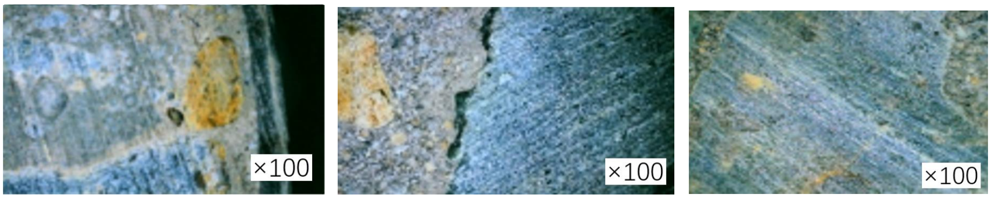

B1S-F50 B2S-F50 B3S-F50

（c）

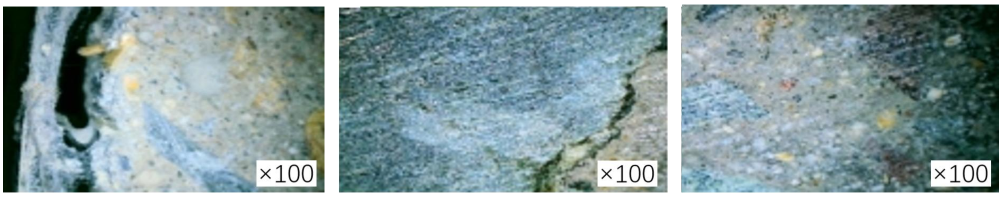

B1S-F100 B2S-F100 B3S-F100

（d）

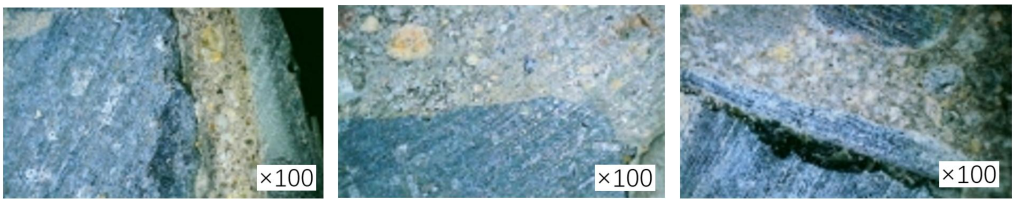

G1S-F50 G2S-F50 G3S-F50

（e）

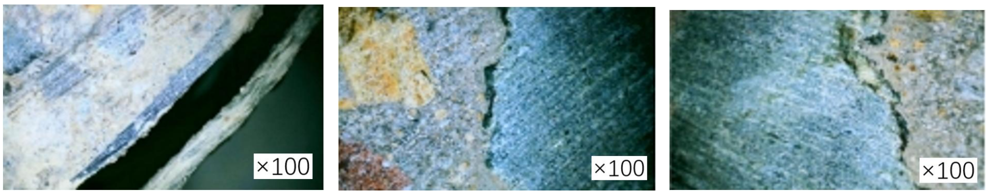

G1S-F100 G2S-F100 G3S-F100

（f）

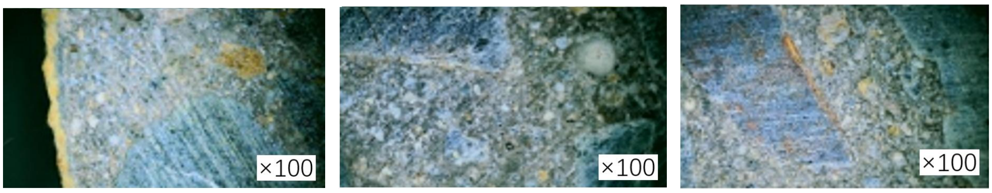

A1S-F50 A2S-F50 A3S-F50

（g）

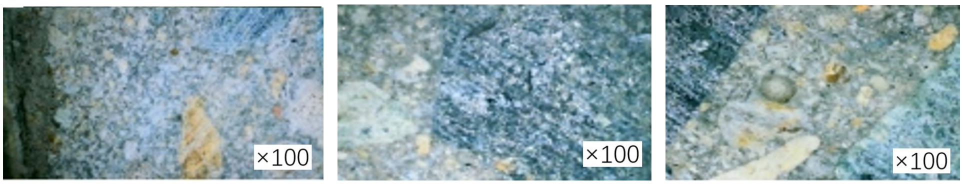

A1S-F100 A2S-F100 A3S-F100

(h)

Fig 21. Microstructure of the FRP reinforced specimen: (a) CFRP reinforced specimens subjected to 50 cycles of salt freeze cycles, (b) CFRP specimens subjected to 100 cycles of salt freeze cycles; (c) BFRP specimens subjected to 50 cycles of salt freeze cycles, (d) BFRP specimens subjected to 100 cycles of salt freeze cycles, (e) GFRP specimens subjected to 50 cycles of salt freeze cycles, (f) GFRP specimens subjected to 100 cycles of salt freeze cycles, (g) AFRP specimens subjected to 50 cycles of salt freeze cycles, (h) AFRP specimens subjected to 100 cycles of salt freeze cycles

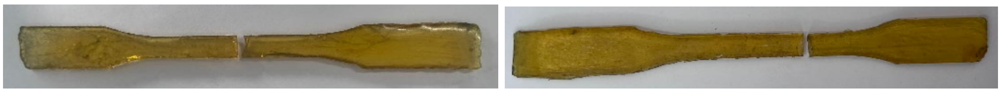

(a) (b)

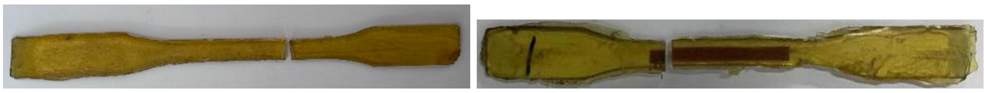

(c) (d)

Fig 22. Damage pattern of epoxy resin:(a) EPN-F0, (b) EPAC-F100, (c)EPAL-F100, (d) EPSA-F100

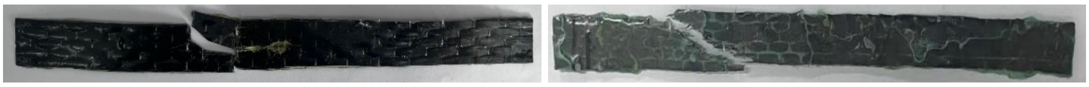

CSN-F0 CSAC-F100

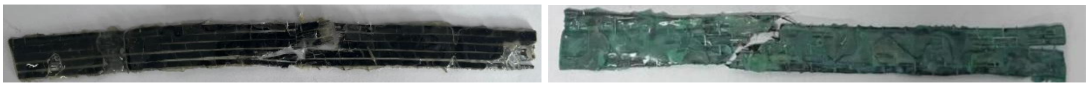

BSN-F0 BSAC-F100

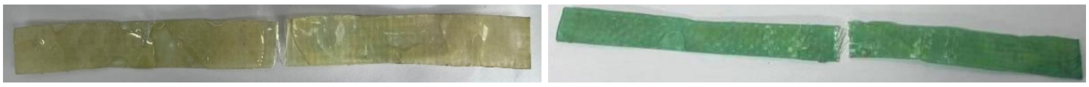

GSN-F0 GSAC-F100

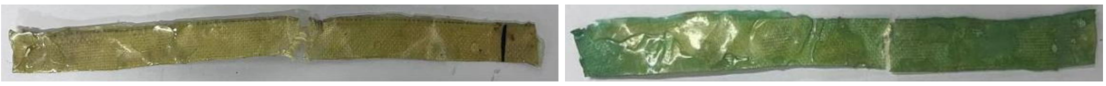

ASN-F0 ASAC-F100

Fig 23. Damage patterns of FRP sheets under acid-freeze coupling erosion

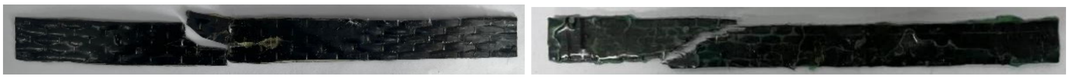

CSN CSAL-F100

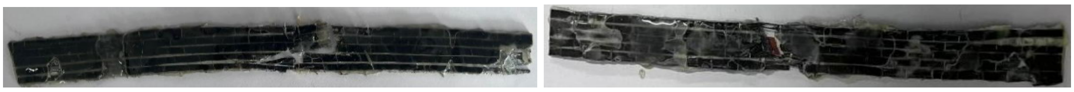

BSN BSAL-F100

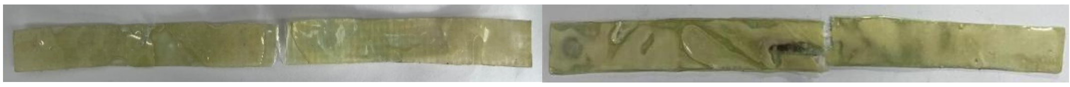

GSN GSAL-F100

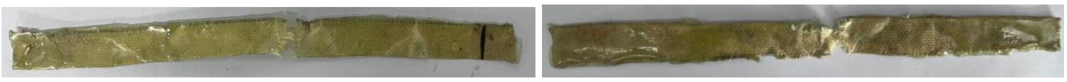

ASN ASAL-F100

Fig 24. Damage patterns of FRP sheets under alkali-freeze coupling erosion

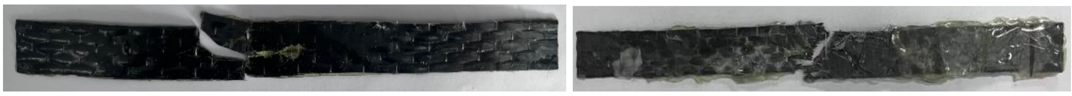

CSN CSSA-F100

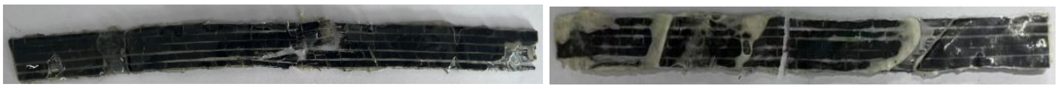

BSN BSSA-F100

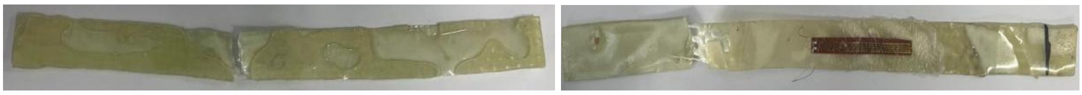

GSN GSSA-F100

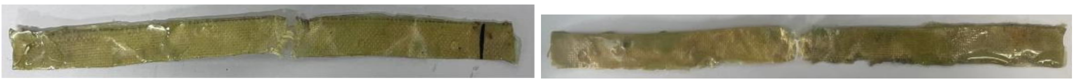

ASN ASSA-F100

Fig 25. Damage patterns of FRP sheets under salt-freeze coupling erosion

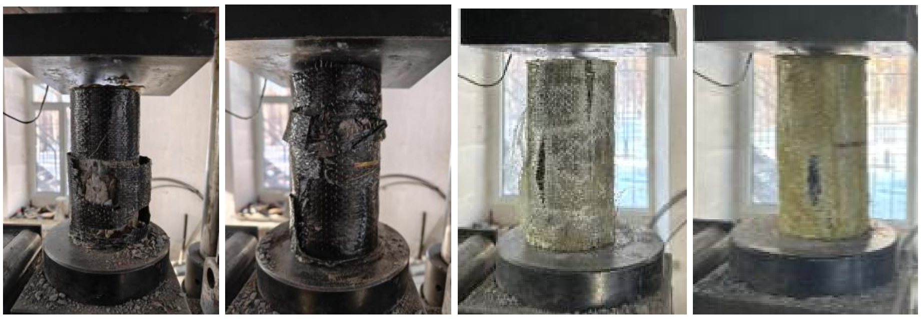

CCAC-F100 BCAC-F100 GCAC-F100 ACAC-F100

（a）

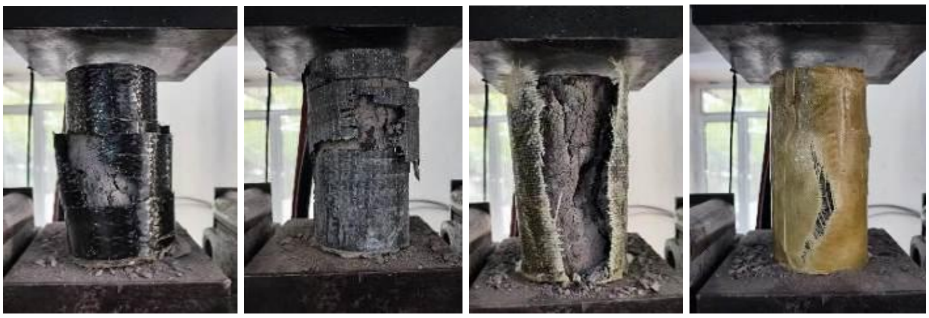

CCAL-F100 BCAL-F100 GCAL-F100 ACAL-F100

（b）

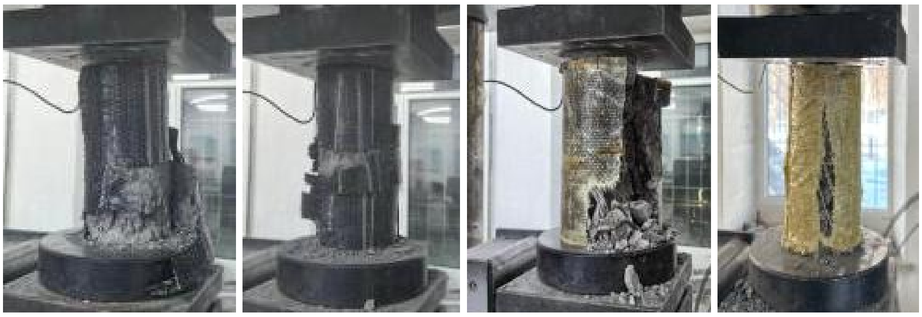

CCSA-F100 BCSA-F100 GCSA-F100 ACSA-F100

（c）

Fig 26. compressive failure modes of FRP reinforced specimens and unreinforced specimens: （a）Under acid freezing coupled erosion conditions, （b）Under alkaline freezing coupled erosion conditions,（c）Under salt freezing coupled erosion conditions

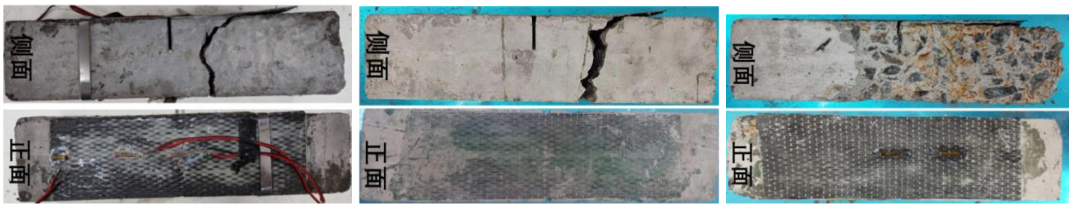

CPN-F0 CPAC-F50 CPAC-F100

（a）

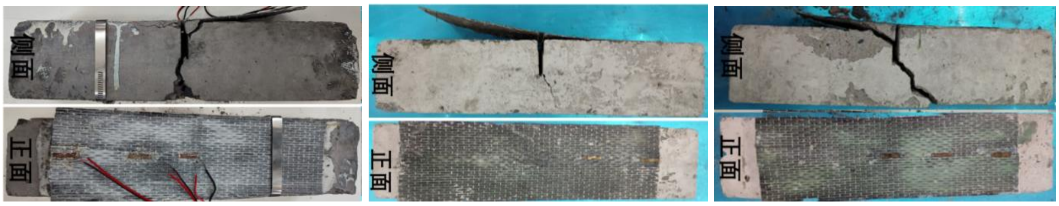

BPN-F0 BPAC-F50 BPAC-F100

（b）

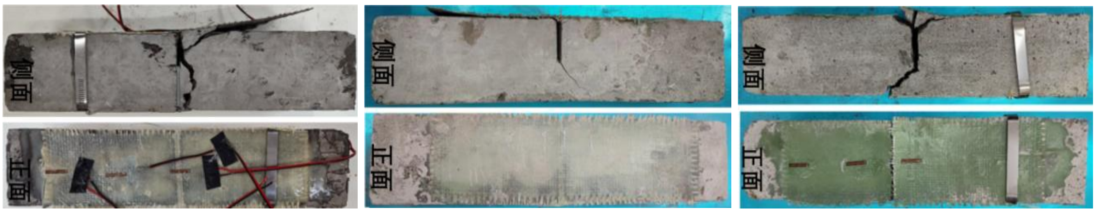

GPN-F0 GPAC-F50 GPAC-F100

（c）

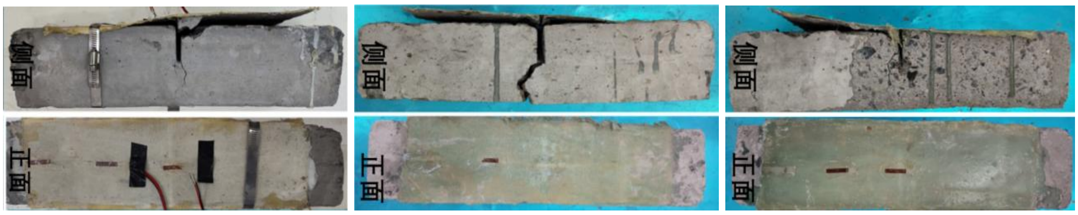

APN-F0 APAC-F50 APAC-F100

（d）

Fig. 27 Failure modes of prismatic specimen under acid-freeze coupling erosion: （a）Failure modes of Carbon fiber reinforced prismatic specimen; （b）Failure modes of basalt fiber reinforced prismatic specimen; （c）Failure modes of glass fiber reinforced prismatic specimen; （d）Failure modes of aramid fiber reinforced prismatic specimen

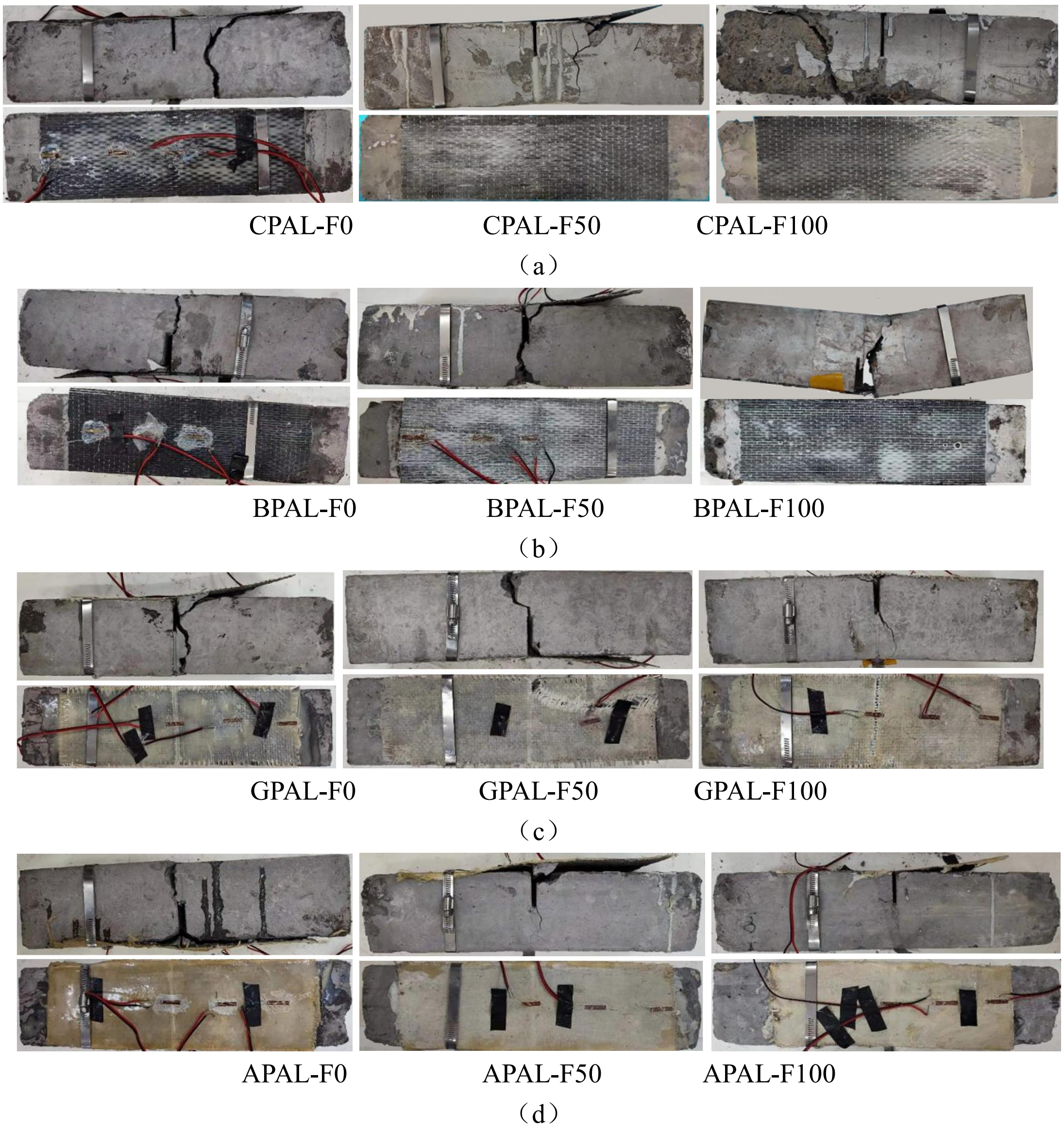

Fig. 28 Flexural failure patterns of FRP reinforced specimens and unreinforced specimens under alkali-freeze coupling erosion （a）Failure modes of Carbon fiber reinforced prismatic specimen; （b）Failure

modes of basalt fiber reinforced prismatic specimen; （c）Failure modes of glass fiber reinforced prismatic specimen; （d）Failure modes of aramid fiber reinforced prismatic specimen

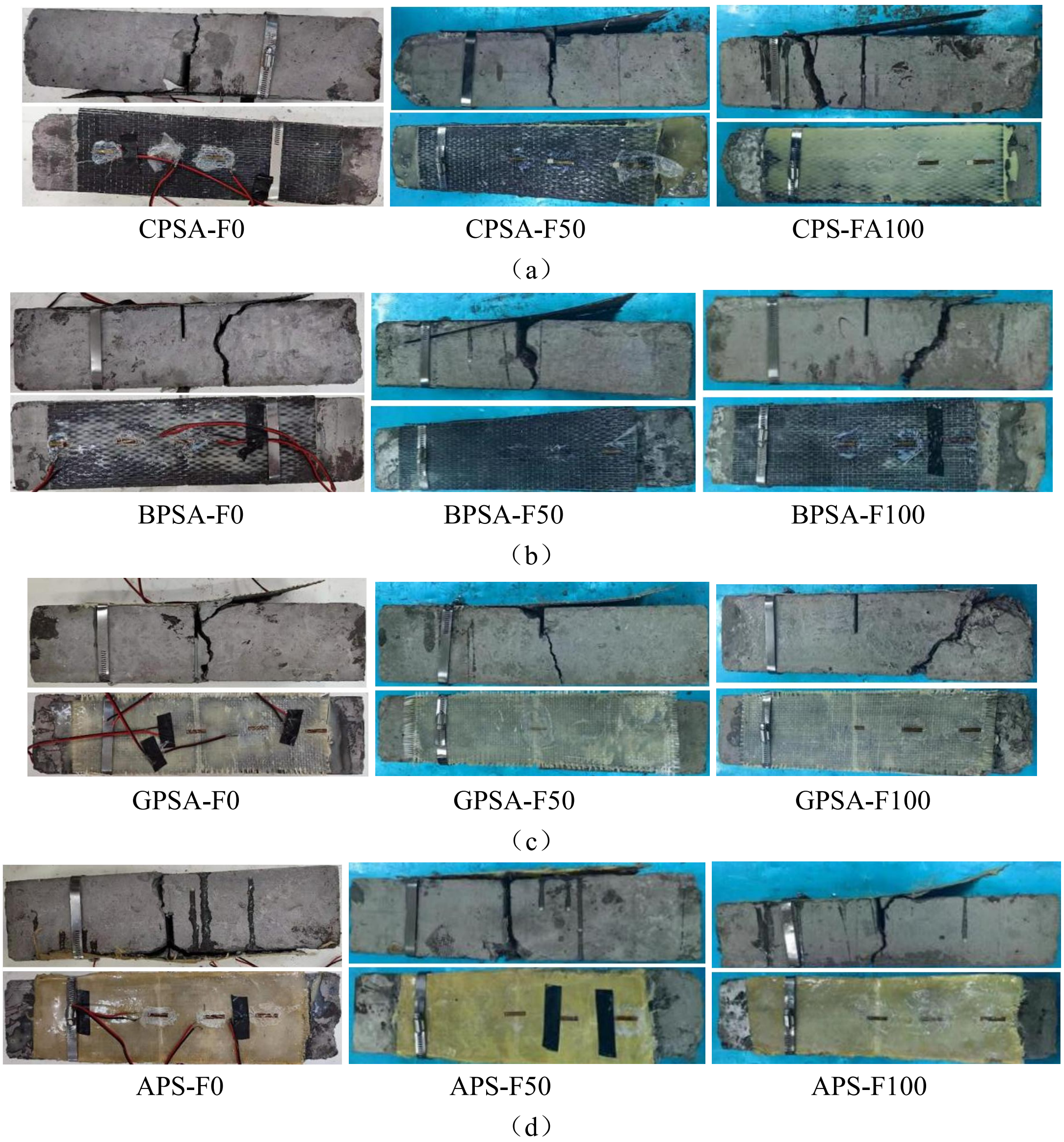

Fig. 29 Flexural failure patterns of FRP reinforced specimens and unreinforced specimens under salt-freeze coupling erosion （a）Failure modes of Carbon fiber reinforced prismatic specimen; （b）Failure modes of basalt fiber reinforced prismatic specimen; （c）Failure modes of glass fiber reinforced prismatic specimen; （d）Failure modes of aramid fiber reinforced prismatic specimen

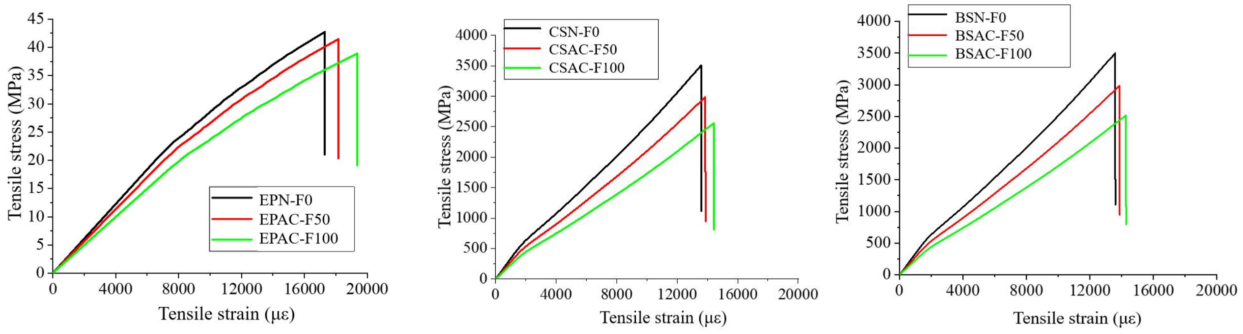

(a) (b) (c)

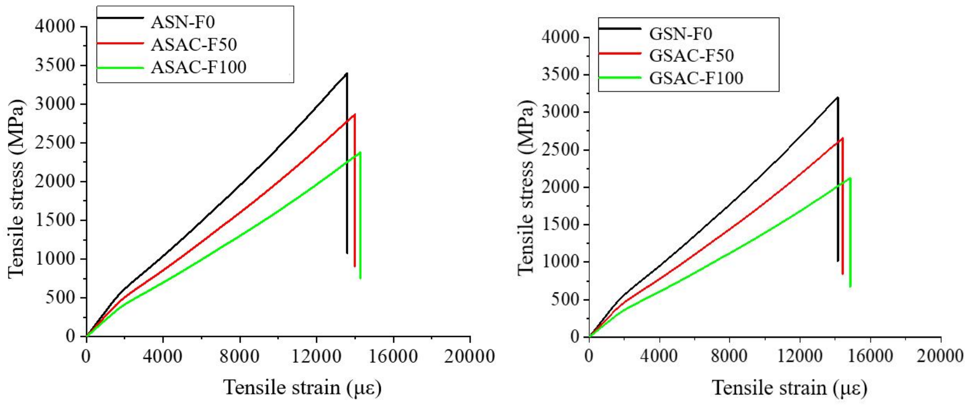

(d) (e)

Fig 30. Stress-strain curve of fiber sheet in coupled acid-freeze-thaw cycle environment: (a) epoxy resin, (b) CFRP sheet, (c)BFRP sheet, (d) AFRP sheet, (e) GFRP sheet

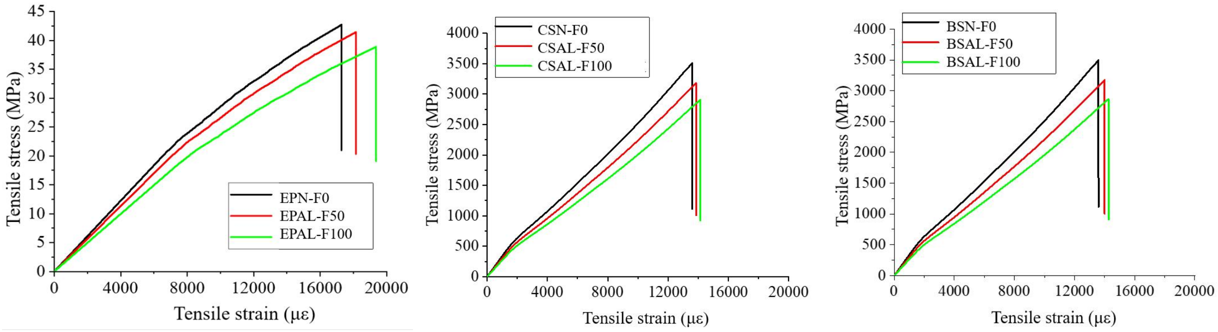

(a)epoxy resin (b) CFRP sheet (c)BFRP sheet

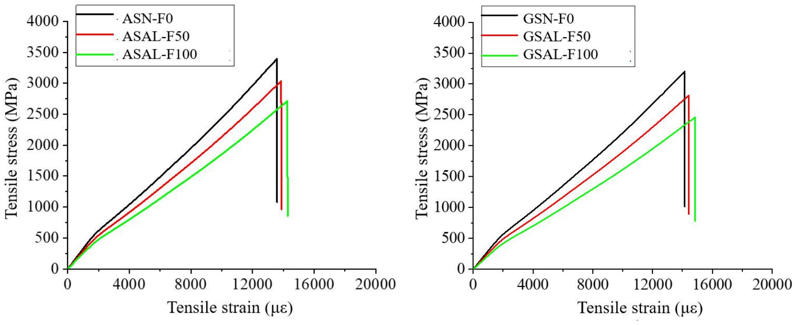

(d) AFRP sheet (e) GFRP sheet

Fig 31. Stress-strain curve of sheet under alkali-freeze-thaw cyclic coupling environment: (a) epoxy resin, (b) CFRP sheet, (c)BFRP sheet, (d) AFRP sheet, (e) GFRP sheet

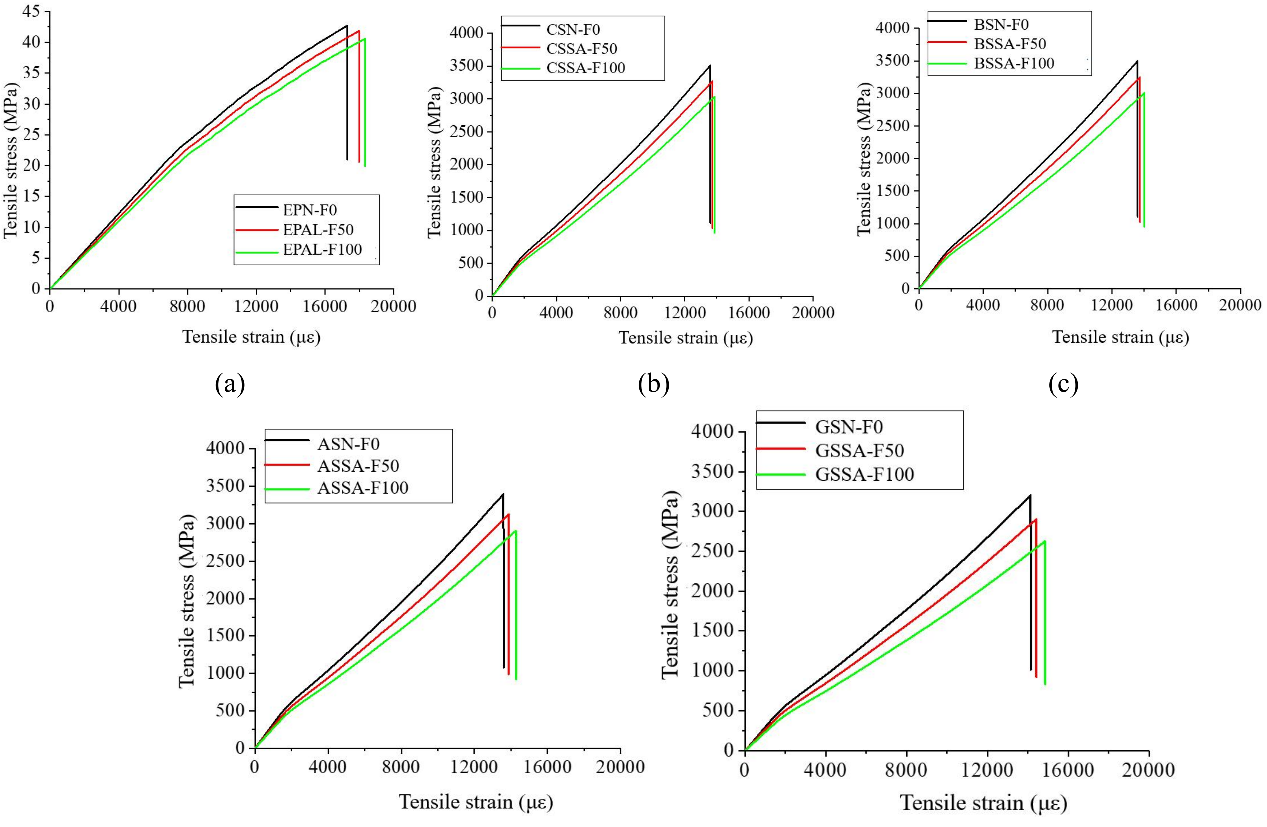

(d) (e)

Fig 32. Stress-strain curves of epoxy resin and FRP sheet in coupled salt-freeze-thaw cycle environment: (a) epoxy resin, (b) CFRP sheet, (c)BFRP sheet, (d) AFRP sheet, (e) GFRP sheet

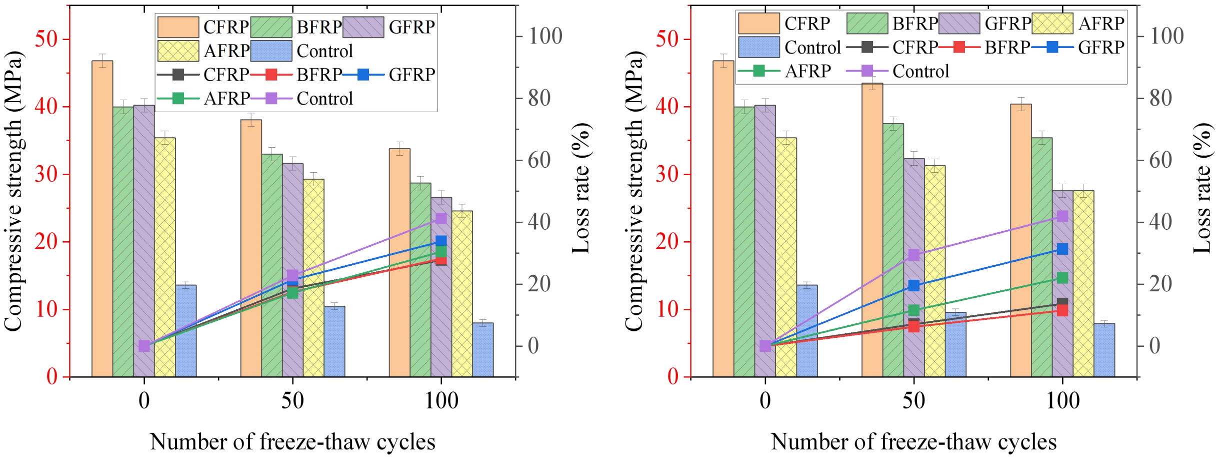

(a) (b)

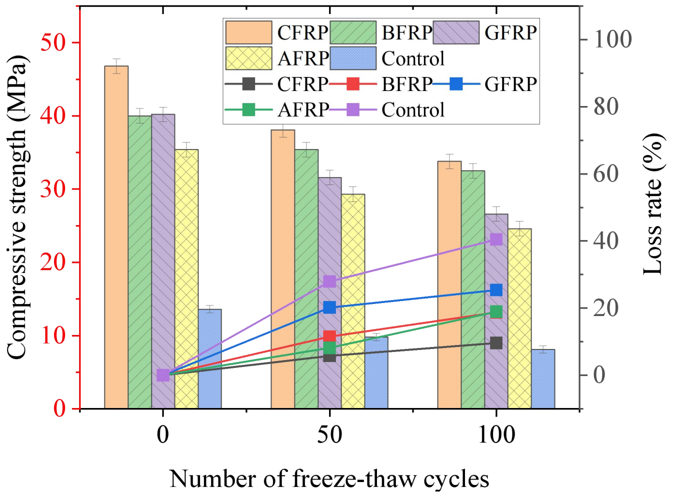

(c)

Fig 33. Compressive strength and strength loss rate of cylindrical specimens under different freeze-thaw cycles:(a) acid-freeze cycle erosion, (b) alkali-freeze cycle erosion, (c) salt-freeze cycle erosion

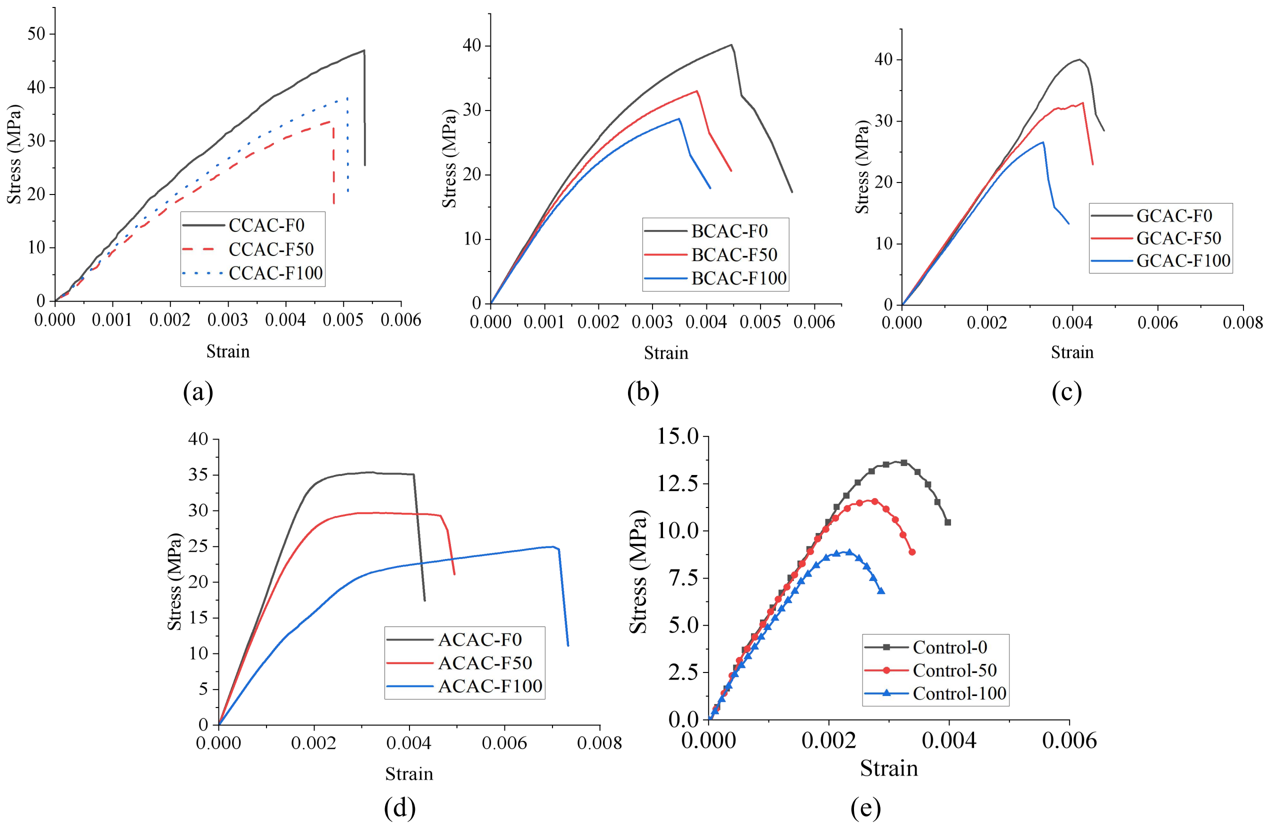

Fig 34. Uniaxial compressive stress-strain curve of cylindrical specimen under coupled acid-freeze erosion: (a)CFRP reinforced specimen, (b) BFRP reinforced specimen, (c) GFRP reinforced specimen, (d) AFRP reinforced specimen, (e)Control group specimen

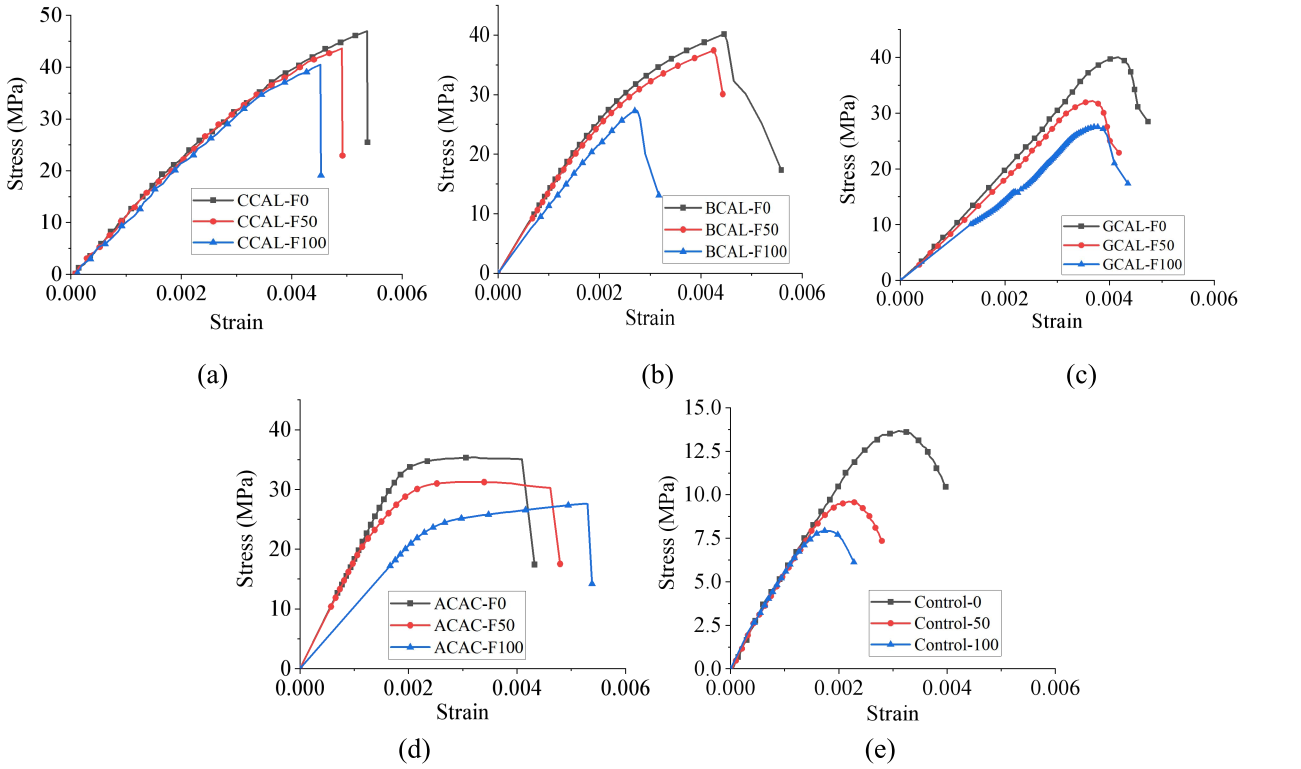

Fig 35. Uniaxial compressive stress-strain curve of cylindrical specimen under coupled alkali-freeze erosion: (a)CFRP reinforced specimen, (b) BFRP reinforced specimen, (c) GFRP reinforced specimen, (d) AFRP reinforced specimen, (e)Control group specimen

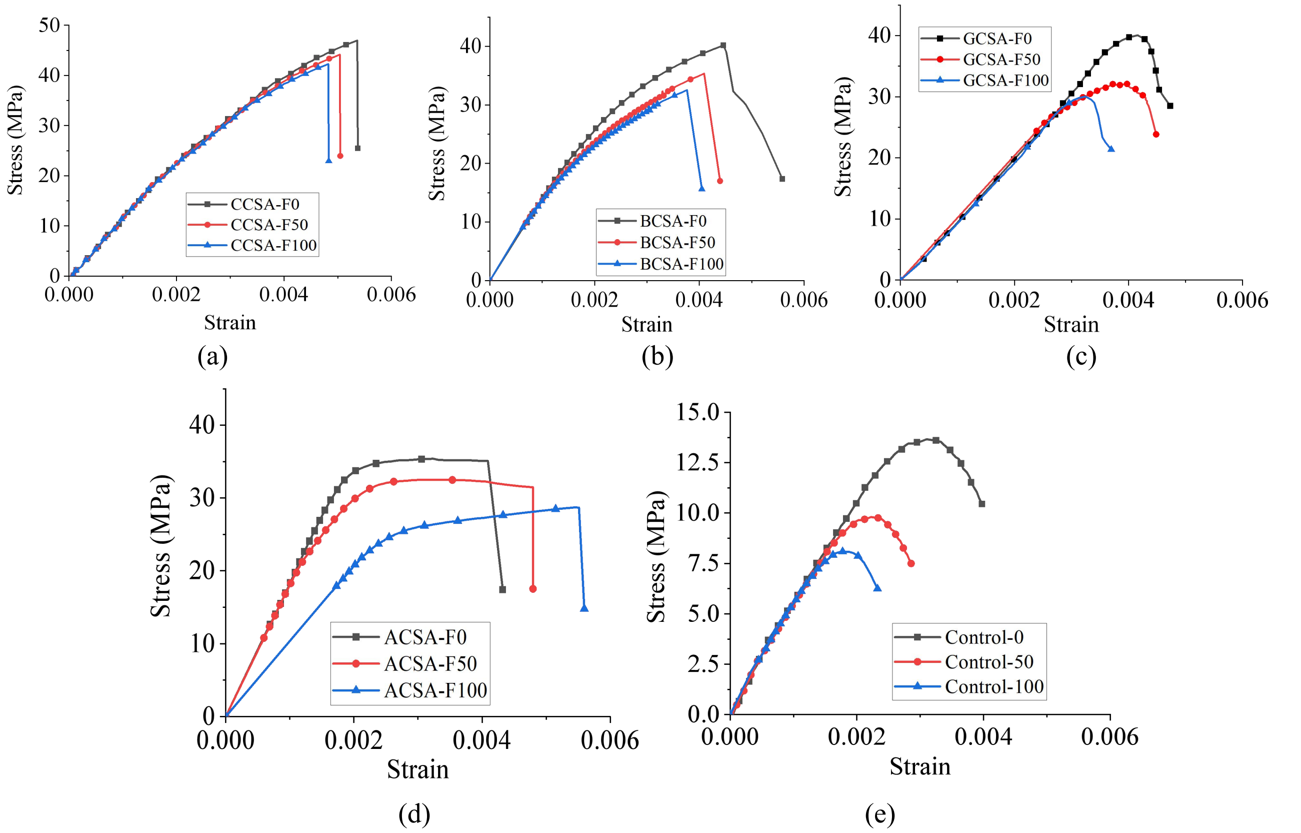

Fig 36. Uniaxial compressive stress-strain curve of cylindrical specimen under coupled salt-freeze erosion: (a)CFRP reinforced specimen, (b) BFRP reinforced specimen, (c) GFRP reinforced specimen, (d) AFRP reinforced specimen, (e)Control group specimen

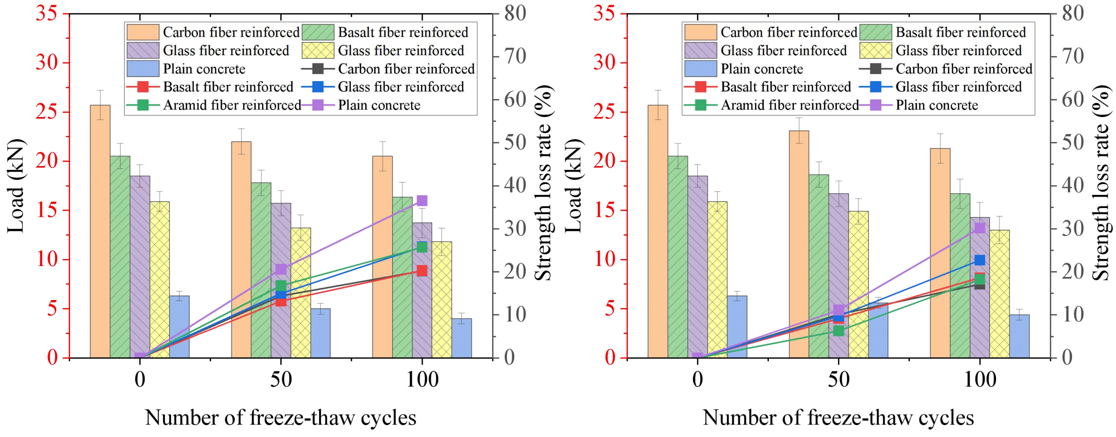

(a) (b)

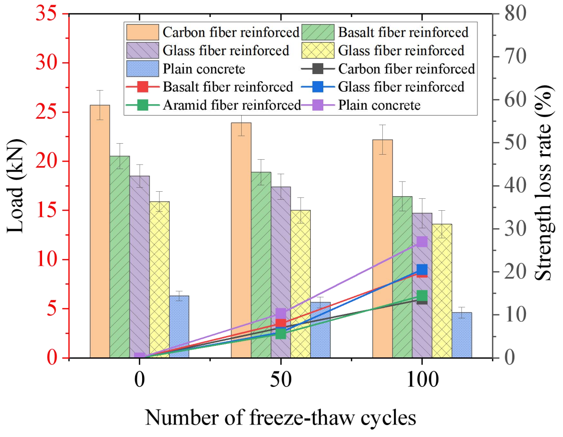

(c)

Fig 37. Flexural strength and loss rate: (a) coupled acid-freeze-thaw cyclic erosion; (b) coupled alkali-freeze-thaw cyclic erosion; (c) coupled salt-freeze-thaw cyclic erosion.

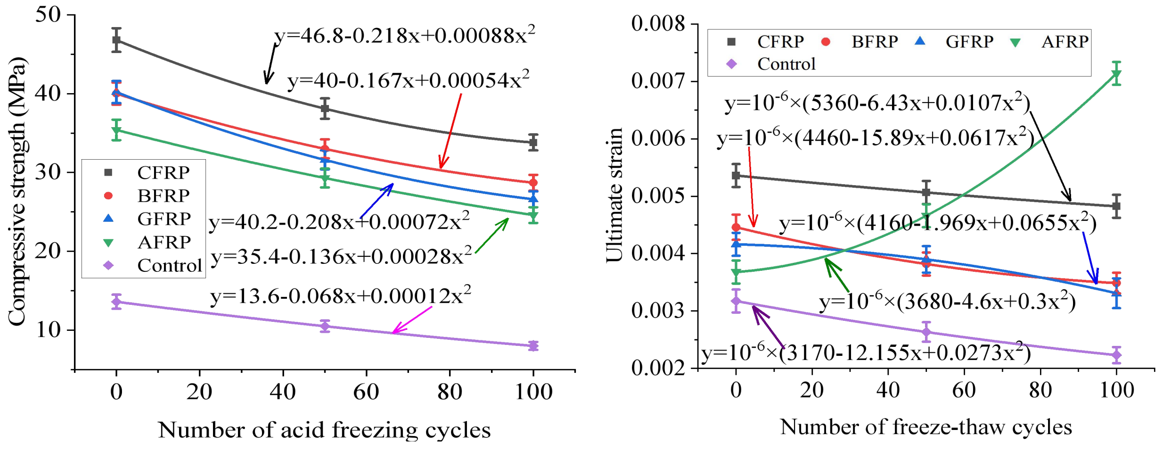

（a） （b）

Fig. 38 The fitting formula of compressive strength and ultimate strain of acid-freeze-thaw cyclic coupled erosion group:(a) compressive strength, (b) ultimate strain

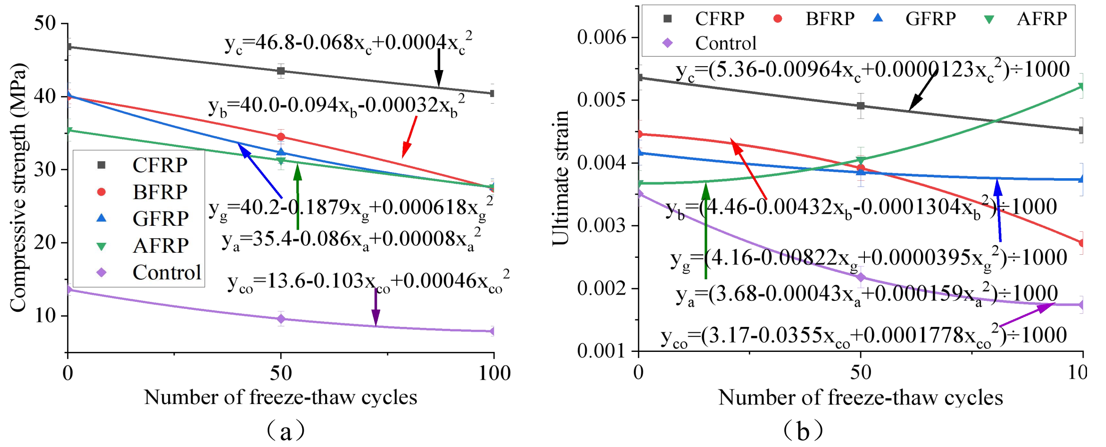

Fig 39. The fitting formula of compressive strength and ultimate strain of alkali-freeze-thaw cyclic coupled erosion specimen:(a) compressive strength, (b) ultimate strain

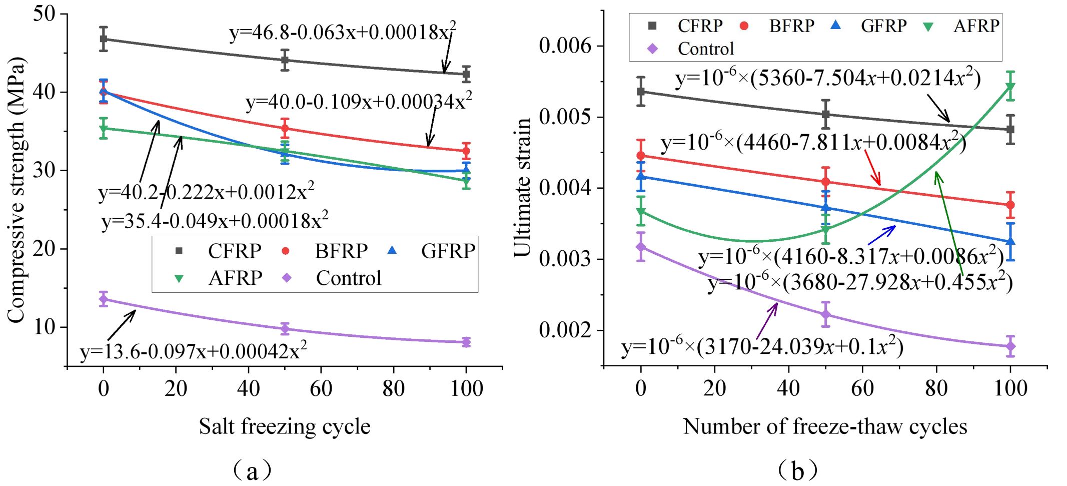

Fig 40. The fitting formula of compressive strength and ultimate strain of salt-freeze-thaw cyclic coupled erosion specimen

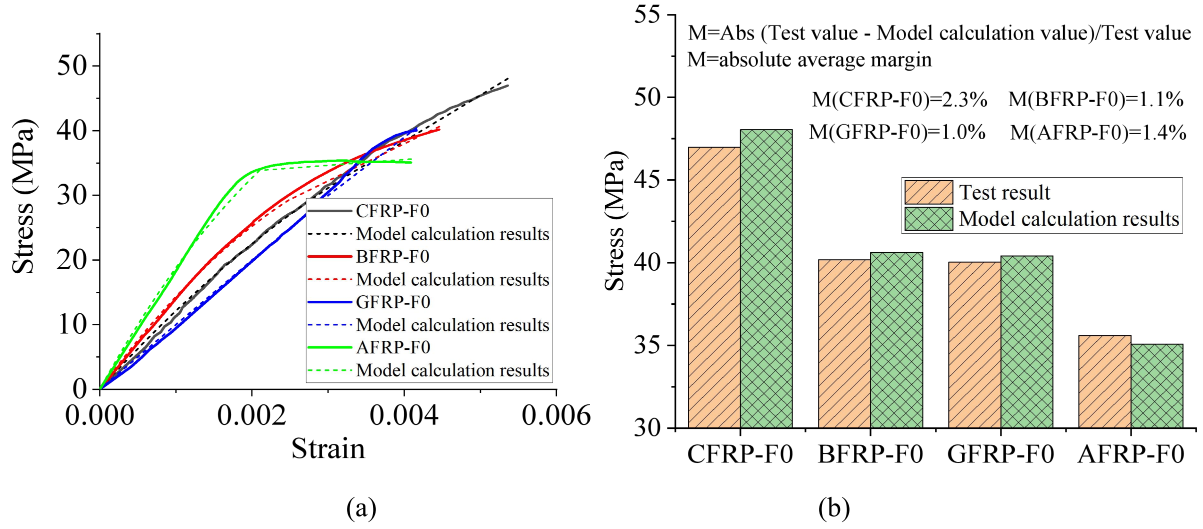

Fig 41. Comparison of calculated and experimental, (a) stress-strain curves, (b)maximum stress
